# The photochemical thiol–ene reaction as a versatile method for the synthesis of glutathione *S*-conjugates targeting the bacterial potassium efflux system Kef†
†Electronic supplementary information (ESI) available: Further experimental details and NMR spectra. See DOI: 10.1039/c5qo00436e



**DOI:** 10.1039/c5qo00436e

**Published:** 2016-02-26

**Authors:** Jess Healy, Tim Rasmussen, Samantha Miller, Ian R. Booth, Stuart J. Conway

**Affiliations:** a Department of Chemistry , Chemistry Research Laboratory , University of Oxford , Mansfield Road , Oxford , OX1 3TA , UK . Email: stuart.conway@chem.ox.ac.uk ; Email: jess.healy@ucl.ac.uk; b Department of Pharmaceutical and Biological Chemistry , UCL School of Pharmacy , University College London , 29/39 Brunswick Square , WC1N, 1AX , UK; c Institute of Medical Sciences , University of Aberdeen , Foresterhill , Aberdeen , AB25 2ZD , UK

## Abstract

The thiol–ene coupling reaction is emerging as an important conjugation reaction that is suitable for use in a biological setting. Here, we explore the utility of this reaction for the synthesis of glutathione-*S*-conjugates (GSX) and present a general, operationally simple, protocol with a wide substrate scope. The GSX afforded are an important class of compounds and provide invaluable molecular tools to study glutathione-binding proteins. In this study we apply the diverse library of GSX synthesised to further our understanding of the structural requirements for binding to the glutathione-binding protein, Kef, a bacterial K^+^ efflux system, found in many bacterial pathogens. This system is vital to the survival of bacteria upon exposure to electrophiles, and plays an essential role in the maintenance of intracellular pH and K^+^ homeostasis. Consequently, Kef is an appealing target for the development of novel antibacterial drugs.

## Abstract 2

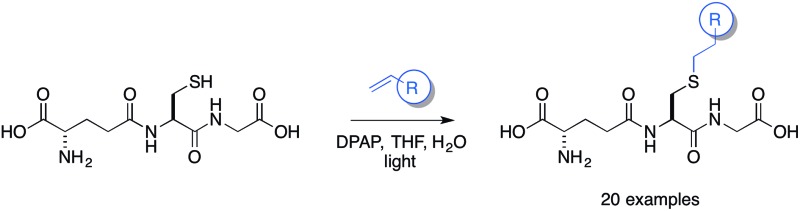
The thiol–ene coupling reaction is emerging as an important conjugation reaction that is suitable for use in a biological setting.

## Introduction

The rapid and continual rise of bacterial resistance to many frontline antibiotic treatments necessitates the urgent identification of novel antibiotic targets.^1^ Consequently, the development of small molecule ligands that modulate the activity of such targets will be essential in the validation of their therapeutic potential. The bacterial potassium (K^+^) efflux system, Kef, is a K^+^/H^+^ antiporter that plays a vital role in promoting cell survival by modulating cytoplasmic pH during electrophilic insult. Kef-like systems are found across the spectrum of bacteria, including in the ESKAPE pathogens, which are responsible for the majority of hospital acquired infections.^2^

**Fig. 1:**
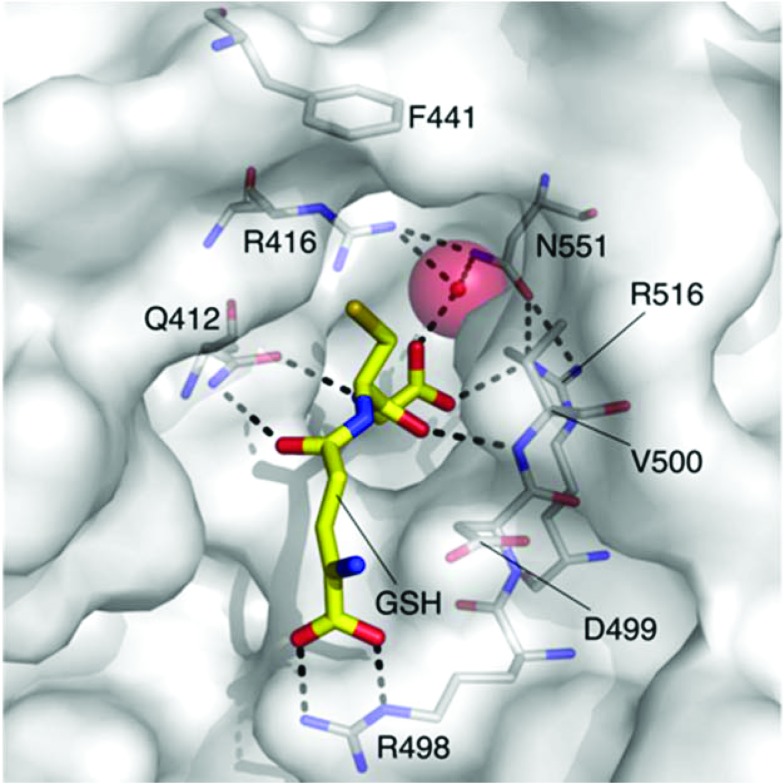
The GSH (carbon = yellow) binding site of *E. coli* KefC, highlighting key residues. This figure was generated with PyMOL using the GSH bound *E. coli* KefC structure (PDB: ; 3L9W).

The Kef system integrates detoxification of electrophiles *via* GSH metabolites with activation of K^+^ efflux. Kef's protective effect stems from the associated drop in the cytoplasmic pH on activation of K^+^ efflux, which is thought to result in protonation of the nucleophilic groups on DNA and proteins – preventing damage caused by exposure to electrophiles.^3^ Previous work has shown the magnitude and rate of activation of Kef to be a vital determinant in cell survival on exposure to toxic electrophiles. Kef thus presents an appealing target for antibiotic drug development.^4,5^ The archetypal *E. coli* Kef system is a complex multi-domain membrane protein, which contains a cytosolic ligand-binding KTN domain. It is inactive in the presence of GSH, and activated by GSH conjugates (GSX) formed on exposure of the cell to electrophiles. Evidence garnered through a combination of classical mutagenesis and crystallographic studies led to the proposal of a model for regulation of K^+^ efflux by the negative and positive effectors, GSH and GSX, respectively.^3^ The peptide core is tethered at either end by a number of key basic residues, R416, R516 and N551. Mutagenesis had previously identified these residues as vital for GSH/GSX recognition,^6^ and crystallography confirmed their importance as key contacts for the peptide backbone (Fig. 1). Crystallography also suggested a conformational change on binding of GSX, particularly around residue F441 that caps the thiol-binding site in what is proposed to be a ‘closed’ conformation. This hypothesis was tested by site directed mutagenesis, where conservative changes (*e.g.* F441W) had little effect on gating but more disruptive changes (*e.g.* F441L or F441D) resulted in significantly impaired activation of K^+^ efflux.^3^ Thus F441 was proposed to be involved in activation of K^+^ efflux. ‘Chemical mutagenesis’, *i.e.* investigation of the effect of varying the nature of the thiol substituent on both binding and gating, was consistent with the proposed model for channel activation.^7^ In our previous study, a number of ESG (*S-N*-ethylsuccinimidyl glutathione) analogues were examined and it was found that larger substituents on the nitrogen atom of the succinimidyl ring including ^*t*^Bu (*K*_D_ = 400 nM), Bn and Cy had a higher affinity for Kef than the parent compound. Additionally, open chain analogues containing 5, 6 or 8 (*K*_D_ = 4.4 μM) carbons bound with similar affinity to ESG, indicating that an increase in hydrophobicity and size corresponded to an increase in affinity for the target. However, examination of the effect of these GSX on the rate of K^+^ efflux, revealed a differentiation between affinity and gating. The structurally rigid succinimidyl analogues were efficient activators, whereas the more flexible analogues resulted in a lower rate of K^+^ efflux, despite similar affinities. These data suggest that a degree of structural rigidity in the S-substituent is required for efficient gating of Kef.

The ligands employed in this work were exclusively formed by conjugate addition of GSH to a range of enones.^7^ While this approach was informative in revealing that both steric bulk and structural rigidity are required for Kef activation, the range of sulfur substituents that we could employ were necessarily limited by the requirement for electrophilic reactivity. Therefore, we sought to employ alternative chemistry to conjugate the thiol group of GSH to a range of structurally diverse moieties. Recent advances in the photochemical thiol–ene coupling (TEC) reaction led us to investigate the use of this chemistry to produce derivatives of GSH.

The TEC reaction^8–10^ is emerging as a useful tool in biologically-important conjugation reactions.^11^ This transformation, which occurs between thiols and alkenes, has many attractive features as it often fulfils the ‘click’ criteria as defined by Sharpless *et al.* ^12^ The biocompatibility of the reaction conditions also provides a viable alternative to the Cu-catalysed Huisgen cycloaddition for the ligation of peptides and proteins.^12–14^ Alternatives to the Cu-catalysed Huisgen cycloaddition are important as the use of this reaction can be complicated by the presence of the Cu catalyst.^15,16^ Until recently, the TEC had been predominantly employed in polymer and materials chemistry, but recent work has firmly established this reaction in the field of bioconjugation, where it has found applications in glycobiology, the synthesis of thioglycosides,^17–21^ the detection of thio-phosphorylated proteins,^22^ protein spin labelling,^23^ tandem application with native chemical ligation for the synthesis of S-modified peptides,^24^ lipidated peptides,^25^ the development of lipophilic amino sugar libraries,^26^ and the synthesis of stapled peptides.^27^ Waldmann *et al.* have utilised this methodology in the synthesis of S-lipidated cysteine derivatives, and more recently to the immobilisation of proteins in microarrays.^28–31^ The TEC has also found wide application in polymer chemistry,^32^ and biopolymer applications, for example the synthesis of glycol-microspheres.^33^ Despite these successes, Dondoni^11^ noted that TEC conditions are not always general and that significant optimisation can be required for each individual system. Additionally, considering the importance of glutathione (GSH, **4**) in biological processes, there have been limited examples of the application TEC to this ubiquitous peptide and in each case GSH modification was not the main focus of these studies.^17–19,34^ Here, we explore the utility of the TEC reaction to generate a diverse library of GSX. This library was subsequently employed to investigate the requirements for high affinity to the ligand-binding domain of the Kef system from a model organism, *Shewanella denitrificans* (*Sd*KefQCTD).^7^

## Results and discussion

### Optimisation of thiol–ene coupling conditions

**Scheme 1:**
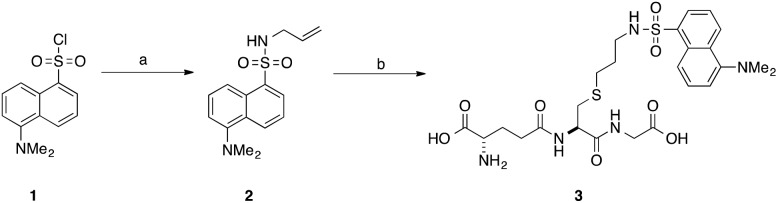
Synthesis of *S*-dansylglutathione (DNGSH) **3**. Reagents and conditions: a. allylamine, DIPEA, CH_2_Cl_2_, 100% b. GSH, DPAP, TCEP·HCl, *hν*, THF/H_2_O, 40%.^7^

Our interest in the TEC reaction was motivated by our desire to synthesise a stable *S*-dansyl-labelled GSH derivative (**3**, Scheme 1). This molecule was required as a fluorescent probe to detect binding of small molecules to Kef.^7^ Direct reaction of dansyl chloride with the thiol of GSH results in the formation of a scissile S–S bond, which rearranges to the more stable *N*-acyl compound. Based on our structure–activity relationship (SAR) studies, we wished to produce a GSX derivative with the fluorophore attached to the thiol. Therefore, we adopted a strategy reported by Waldmann *et al.* ^31^ and synthesised *N*-allyl-5-(dimethylamino)naphthalene sulfonamide (**2**) from dansyl chloride (**1**). Installation of an allyl group provided a synthetic handle for linkage of the fluorophore to GSH *via* the TEC, forming a stable thioether bond. Using conditions similar to those reported by Fiore *et al.* ^35^ and Staderini *et al.*,^36^ a household UV lamp (4 × 15 W, 365 nm) was used as the light source and 2,2-dimethoxyphenyl acetophenone (DPAP) as the initiator. Due to the disparate solubilites of the reagents, a mixture of THF and water (1 : 2) were used as solvents, allowing for partial solubilisation of the alkene substrate. Initial attempts provided the desired compound in yields of 8–14% (Table 1, entries 1–3). The primary product, however, was the disulfide (GSSG). Addition of a reducing agent tris(2-carboxyethyl)phosphine (TCEP·HCl) resulted in a significant improvement in yield to 40% (Table 1, entry 4). Thorough degassing of solvents was found to have no effect on the observed yield (Table 1, entries 5 and 6).

**Table 1:** Optimisation of reaction conditions for the synthesis of **3a** ^*a*^

Entry	GSH : dansyl	*t* (h)	Yield^*b*^ (%)
1	2 : 1	17	14
2	2 : 1	34	8
3	4 : 1	5	13
4^*c*^	4 : 1	5	40
5^*c*^	4 : 1	5	32
6	4 : 1	5	18

^*a*^0.5 eq. DPAP used in all cases. Entry 5: degassed THF/H_2_O, entry 6: degassed DMF/H_2_O.

^*b*^Yields quoted are isolated yields following purification by RP C-18 silica gel column chromatography.

^*c*^Denotes addition of TCEP·HCl.

**Fig. 2:**
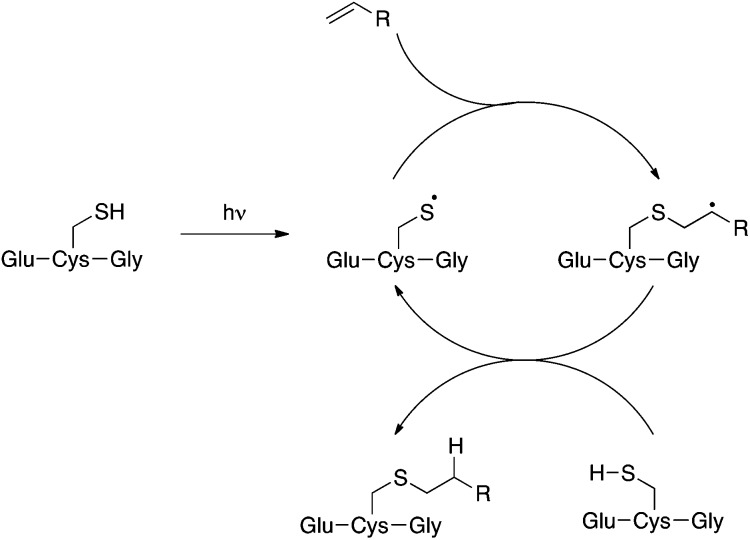
The thiol–ene coupling reaction.

Compound **2** is a challenging substrate for the TEC. The thiyl radical reacts more readily with electron-rich enes due to its electrophilic nature, thus the initial rate of addition is expected to be slow for this compound.^14,32,37^ Additionally, compound **2** contains a heteroatom in the allylic position making it sensitive to elimination to the allyl sulfide after the addition step (Fig. 2).^37,38^ Given our encouraging results with this challenging ene we were keen to further investigate the scope of this reaction for the generation of structurally distinct GSX as probe compounds for Kef.

**Scheme 2:**
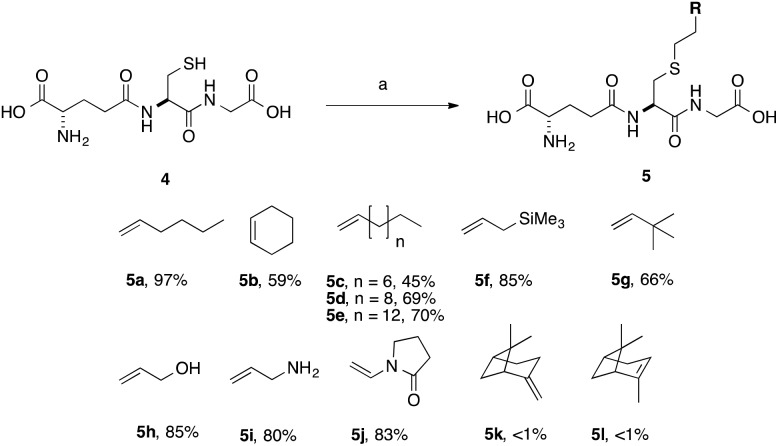
Scope of thiol–ene coupling to alkyl and heteroalkyl substrates. Reagents and conditions: a. alkene (1 eq.), DPAP, THF/H_2_O, *hν*. % yields quoted are isolated yields.

We optimised the reaction conditions using the simple terminal alkene, decene, as a model system (Scheme 2, **5c**). Using the conditions that we had previously developed a moderate yield of 45% was obtained (Table 2, entry 1). To simplify the purification of the product we excluded the reducing agent, which had no significant effect on the yield of S-labeled peptide obtained with this simple substrate. This result indicates that the GSSG during the formation of **3** resulted from the slower reaction rate of **2** in the TEC. Shortening the irradiation time was found to decrease the yield (Table 2, entry 2, 15%), while an increase in the irradiation time gave no improvement (Table 2, entry 6). We found, however, that the amount of initiator could be reduced to 20 mol% without any impact on the isolated yield (Table 2, entry 5), when using decene. With standard conditions established using a simple substrate, a series of alkenes were selected to probe the scope of the reaction, and which would provide a diverse SAR profile for Kef.

**Table 2:** Reaction optimisation for simple alkene system (**5c**)^*a*^

Entry	DPAP	*t* (h)	Yield^*b*^ (%)
1	0.5	5	45
2	0.5	1	15
3	0.2	2	5
4	0.2	3	18
5	0.2	5	45
6	0.2	8	31

^*a*^Ratio of GSH : decene (4 : 1), and solvent system THF/H_2_O (1 : 2) in all cases.

^*b*^Yields represent isolated yields following purification by crystallisation from EtOH/H_2_O.

With simple, terminal and unfunctionalised alkenes the yields ranged from 45% to 97% (Scheme 2, **5a**, **c**, **d**, **e**, **g**). Isolation in all cases was by filtration followed by crystallisation from boiling ethanol and water. With hexene as the ene substrate the resulting labeled peptide was obtained in very high yields (97%, **5a**). By comparison, reaction with cyclohexene resulted in a significantly lower yield (59%, **5b**), as expected due to increased reversibility of the initial thiyl radical addition to the internal alkene (Fig. 2).^37^

(–)-β-Pinene (**5l**) and (+)-α-pinene (**5k**) were both very poor TEC substrates, with less than 1% of the desired adduct isolated in both cases. These low yields could result from a number of factors: (1) slow rate of hydrogen radical abstraction by the stable carbon radical, formed by both **5k** and **5l** could mask the expected differences in the rate of reaction with the internal *vs.* external alkene; or (2) rearrangement or fragmentation of the radical intermediate might result in unwanted products. Reaction with heteroatom-containing allylic substrates (**5f**, **h**, **i**) proceeded well in all cases. However, due to the increased solubility of the hydroxyl- and amino-containing adducts (**5h** and **5i**), purification by reverse phase C-18 silica gel column chromatography was required. These compounds are interesting, however, as they provide handles for further synthetic elaboration. The heterocyclic pyrrolidinone (**5j**) was also well tolerated.

**Scheme 3:**
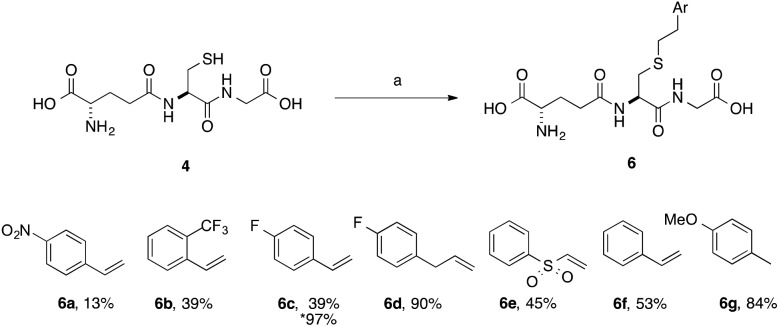
Scope of thiol–ene coupling to aryl substrates. Reagents and conditions: a. alkene (1 eq.), DPAP, THF/H_2_O, *hν*. % yields quoted are isolated yields. * denotes the use of TCEP·HCl.

A series of aryl-substituted, vinyl and allyl alkenes were next investigated (Scheme 3). Styrene (Scheme 3, **6f**) is a poor TEC substrate,^14^ and in our hands yielded the desired adduct in moderate yield (53%). Considering the reaction mechanism (Fig. 2), modification of the electronic properties of the aryl ring by substitution was predicted to affect the overall reaction yield, and this was indeed found to be the case. Addition of electron-withdrawing groups in the *para*-(**6a** and **6c**) or *ortho*-(**6b**) positions of the ring resulted in a decreased yield of the adduct. The electron poor vinyl sulfone (**6e**) also resulted in a low yield of the labelled peptide. The reduced yields in these cases can be rationalised by either the low reactivity of the electrophilic thiyl radical with these electron-poor alkenes, or a slower hydrogen radical abstraction step. For the *para*-nitro analogue, competing reduction to the aniline was also observed, and consequently the purity of **6a** was lower that of the other GSX in this series. Addition of a methylene unit to the 4-fluoro derivative improved the observed yield (**6d**, 90% *cf.* **6c**, 39%), due to the loss of conjugation with the aromatic system. Conversely, addition of a *para*-methoxy substituent resulted in an increase in yield of the desired adduct (**6g**, 84%), due to the increased electron density of the alkene in this case. It can be seen from the data presented here that electron-rich alkenes are better substrates for this reaction due to their greater reactivity with the electrophilic thiyl radical. This observation is consistent with the reactivities trends observed in polymer chemistry.^14^

Electron-poor alkenes provide lower yields in this reaction as they react less readily with the electrophilic thiyl radical. Additionally, we found that for particularly challenging substrates the yield may be increased by modification of the electronic properties of the ene *via* appropriate substitution, or by the addition of TCEP·HCl as a reducing agent (Table 1, entry 4 and Scheme 3, **6c**). This second observation is contrary to a report by Scanlan *et al.*, in which an increase in competing radical desulfurisation was observed in the presence of ^*t*^Bu_3_P as reducing agent, this was not observed in our hands with TCEP as the phosphine reducing agent.^24^

### Investigation of the effect of the nature of the S-substituent on Kef binding

The structurally diverse GSX that we synthesised were evaluated for their ability to bind Kef from *S. denitrificans*. A soluble construct of Kef from *S. denitrificans* (*Sd*KefQCTD), containing the ligand-binding KTN domain, the Q-linker (which links the cytosolic and membrane domains) and a 10-residue regulatory loop from the membrane domain (GHELEVDIEP), was employed for all biophysical analyses and was purified as previously described.^7^ Kef from *S. denitrificans* was used as a model system as it lacks the ancillary cytoplasmic protein subunit KefF, which is required for full activation in *E. coli*, and could complicate biophysical analysis of ligand binding.

Two complementary methods were employed to examine ligand binding to *Sd*KefQCTD: differential scanning fluorimetry (DSF)^39^ and a fluorescence competition assay using DNGSH (**3**). DSF relies on an increase in fluorescence that results from SYPRO orange binding to hydrophobic regions of a soluble protein that are exposed by thermally-induced protein unfolding, giving a melting temperature for the protein (*T*_m_). Repeating the process in the presence of a ligand (that binds to a folded state of the protein) usually results in an increase in *T*_m_. The change in *T*_m_ between the free and ligand-bound protein (Δ*T*_m_) correlates with ligand's affinity for the protein.^40–42^

In depth biophysical evaluation of compound **3**, which is employed in the fluorescence competition assay, has been described previously.^7^ Briefly, the dansyl chromophore is a solvatochromic probe, which is sensitive to the nature of its environment. On binding to *Sd*KefQCTD, *i.e.* a transition from a hydrophilic to a hydrophobic environment, both an increase in the quantum yield of fluorescence of **3** and a hypsochromic shift in the *λ*_max_ of emission are observed. Displacement of the probe by a competing ligand results in a drop in fluorescence intensity that can be used to identify new ligands for *Sd*KefQCTD. Quantitative data can be obtained by titration of *Sd*KefQCTD and **3** with increasing concentrations of the competing ligand, and fitting of the resulting binding isotherm. Good correlation of the data obtained using both methods was observed, as previously reported. Additionally *F*_B_/*F*_L_ values (where *F*_B_ = fluorescence intensity **3** + *Sd*KefQCTD and *F*_L_ = fluorescence intensity **3** + SdKefQCTD + GSX and an *F*_B_/*F*_L_ > 1 indicates binding), have shown good correlation with the affinities obtained using quantitative methods.^7^

**Fig. 3:**
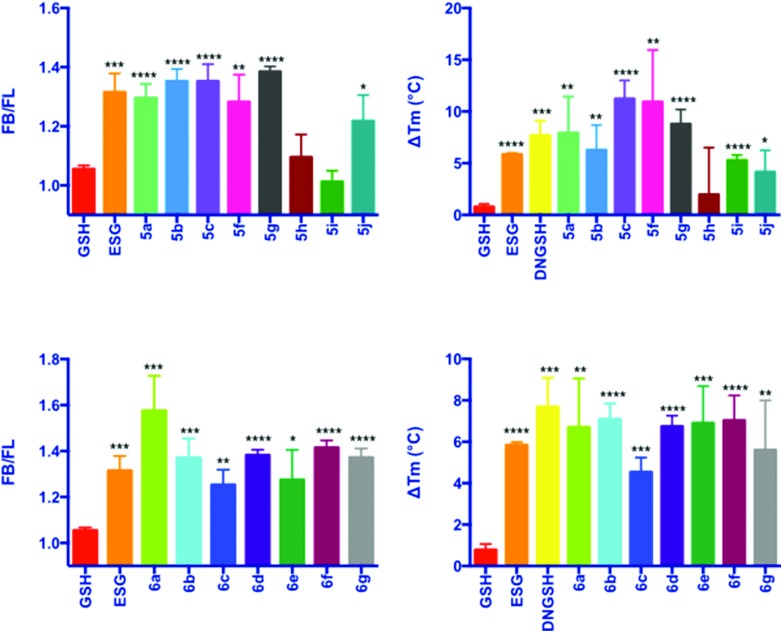
Fluorescence competition (A and C) and DSF (B and D) data for GSX. Error bars represent the standard deviation (*n* = 3). Significance of changes evaluated by Student's *t*-test relative to GSH (where ****p ≤ 0.0001, ***p ≤ 0.001, **p ≤ 0.01. *p ≤ 0.05). *F*_B_/*F*_L_ = FI(Kef + 3)/FI(Kef + 3 + GS-X) and Δ*T*_M_ is the difference in *T*_M_ in the presence of ligand relative to that of the protein alone.

Evaluation of the aliphatic and heteroatom containing GSX (Scheme 2, **5a–5j**) demonstrated that compounds with simple aliphatic substituents bound with a similar affinity to the known activator ESG (*K*_D_ = 12 μM) (Fig. 3A and B).^7^ The smaller, more polar, analogues **5h** and **5i** were found to bind to *Sd*KefQCTD with a lower affinity, similar to that observed for the native ligand GSH (*K*_D_ = 900 μM) (Fig. 3A and B), and a known weak activator of *E. coli* KefC, *S*-lactoyl glutathione (SLG, *K*_D_ = 900 μM). A small recovery in binding efficiency was observed for the larger pyrrolidinone analogue **5j**.

For the aromatic analogues (Scheme 3, **6a–6g**), *Sd*KefQCTD binding of a similar magnitude to the positive control ESG was observed in all cases (Fig. 3C and D). The analogue containing the *para*-nitro (**6a**) substituent and the unsubstituted derivative **6f** were the only cases for which a statistically significant increase in binding affinity relative to ESG was observed in the fluorescence assay (*p* ≤ 0.05 in both cases). Also of note, a reduction in *Sd*KefQCTD affinity was observed for the 4-F derivative (**6c**), which could be recovered through the introduction of an additional methylene unit between the sulfur atom and the aromatic ring (**6d**).

## Conclusions

In conclusion, we have shown that the TEC is a simple, versatile and reliable method for the synthesis of novel GSH derivatives. The conditions that we have developed are operationally simple and high yielding. It is noteworthy that in all cases, no effort was made to exclude oxygen. For most substrates the product could be isolated by simple filtration and purified by crystallisation from ethanol and water. Most significantly, for more challenging substrates the yield can be improved through the addition of a reducing agent (TCEP·HCl).

The diverse library of GSX synthesised using this methodology contained analogues with simple aliphatic (cyclic and acyclic) substituents, small polar heteroatom containing functional groups and a series of substituted styrenes. This set of probe compounds was tested for their ability to bind to *Sd*KefQCTD, and it was found that those ligands containing bulky, hydrophobic substituents bound with greater affinity than those with small polar groups. These data enhance our understanding of Kef SAR, and is invaluable for our continued understanding of the Kef system and attempts to determine whether this protein is a therapeutically useful target for antibacterial drugs. The compounds will also potentially find use in the investigation of other GSH-binding proteins.

## Experimental section

### Strains and plasmids

The strains and plasmids used in this study were described previously.^7^ Briefly, the strain used for protein expression and purification was MJF373 (derived from MJF276 ΔkefFC:kan, Δcrp kefB:Tn10). A soluble construct, pTrc*Sd*KefQCTD, of Kef from *S. denitrificans* was used for protein expression. The construct was derived from pTrc*Sd*KefH_6_ of the Kef gene from *S. denitrificans* OS217 (accession number NC007954.1) starting at K391 with a further 10 amino acid sequence (GHELEVDIEP) fused at the 5′ end (corresponds to a suspected regulatory loop).

### Protein expression and purification

pTrc*Sd*KefQCTD was transformed into the *E. coli* strain MJF373 for expression. The cell culture was grown in LB media (0.5 L, containing 0.1% glucose) at 37 °C until an OD_650_ of 0.8 was reached. After this time the culture was cooled to 30 °C, followed by induction with IPTG (0.8 mM). The cells were incubated at this temperature for a further 3 h, harvested and stored at –80 °C until required. Cell lysis was achieved using a French Press (SLM Aminco) at a pressure of 18 k psi. The lysate was centrifuged (4700 rpm, 30 min, 4 °C) and the supernatant collected and filtered (Sartorius Minisart single use filter, 0.45 μm pore size). The lysate was loaded into a glass column containing His-Select Nickel affinity gel (under gravity at 4 °C), washed with wash buffer (40 mL containing: 50 mM Na–phosphate buffer pH 7.5, 150 mM NaCl, 10% glycerol, 20 mM imidazole). Bound protein was subsequently eluted (50 mM Na–phosphate buffer pH 7.5, 150 mM NaCl, 10% glycerol, 300 mM imidazole). Buffer exchange was achieved using PD-10 columns (GE Healthcare). The purity of resulting protein was verified by SDS-PAGE gel electrophoresis. Protein concentration was determined by UV/VIS spectrophotometry (*ε* = 17 420 M^–1^ cm^–1^).

### Differential scanning fluorimetry

Assays were performed using a Stratagene Mx3005P qPCR (Sybr filter, ex. 492 nm, em. 516 nm). The initial temperature was set to 25 °C, increasing in increments of 1 °C. The *T*_m_ (melting temperature) was identified by fitting to the Boltzmann equation (Prism 5).^39^ The change in unfolding temperature (Δ*T*_m_) was calculated as the shift in *T*_m_ relative to the *T*_m_ of the protein in the absence of any ligand. A student *T*-test was performed to ensure that the changes were statistically significant. Solutions of the ligands under examination were prepared in DMSO or dH_2_O at a final concentration of 100 mM. These stock solutions were subsequently diluted to a concentration of 10 mM in buffer containing 50 mM Na–phosphate, 150 mM NaCl, pH 7.5. A protein master mix was prepared containing *Sd*KefQCTD (40 μM) and Sypro Orange (1 : 1000 dilution, Invitrogen). Ninety-six well plates (Axygen) were prepared using the protein master mix (22.5 μL, 12 μM) and the appropriate ligand (2.5 μL). Controls were performed with dye alone, ligand and dye, and the protein alone.

### Fluorescence measurements

For qualitative competition fluorescence measurements a Perkin Elmer Luminescence Spectrometer was used. Samples were excited at 340 nm and the emission spectra measured between 400 & 600 nm. Samples were prepared containing 6 μM *Sd*KefQCTD and 5 μM DNGSH. A decrease in fluorescence intensity after the addition of the desired competing ligand, at a final concentration of 1 mM, was interpreted as an indication of binding. The data are reported as the ratio of the fluorescence intensity before and after the addition of the ligand under examination at 525 nm (*F*_B_/*F*_L_). A student's *T*-test was performed to determine whether the changes were statistically significant.

### Synthetic chemistry general information

All reagents were purchased from Sigma Aldrich or Alfa Aesar and were used without further purification. The UV light source was provided by a Philips HB175 Facial Solarium (UVA, 365 nm, *P* = 4 × 15 W). Reverse phase column chromatography was carried out on Fluka Ltd silica gel 100 C18-reversed phase, under a positive pressure of compressed air. Analytical TLC analysis was performed using Merck 60 RP-18 F_254_S aluminium-supported thin layer chromatography sheets and visualised using ninhydrin. ^1^H and ^13^C NMR spectra were recorded on a Bruker Avance 400 (400 MHz, 100 MHz, respectively) or Bruker Avance III (500 MHz, 125 MHz, respectively). ^1^H and ^13^C spectra were assigned using 2D NMR experiments including COSY, HSQC and HMBC. Melting points were performed on a Kofler Hotstage microscope and are uncorrected; the crystallisation solvent is shown in parentheses. IR spectra were recorded using a Bruker Tensor 27 spectrometer. Elemental analyses were submitted to the Elemental Analysis Service, London Metropolitan University. Optical rotations were recorded at 20 °C at the sodium D line (589 nm). ESG was prepared as previously reported.^3^ Selected examples of synthetic procedures are included below. Further details are available in the ESI.†

#### *N*-Allyl-5-(dimethylamino)naphthalene-1-sulfonamide (**2**)

To allylamine (21 mg, 28 μL, 0.37 mmol, 1 eq.), and diisopropylethylamine (239 mg, 1.85 mmol, 5 eq.) in CH_2_Cl_2_ (5 mL) a solution of dansyl chloride (100 mg, 0.37 mmol, 1 eq.) in CH_2_Cl_2_ (3 mL) was added. The reaction was stirred at RT overnight. After 18 h the reaction was adjudged to be complete by TLC analysis, concentrated *in vacuo* and purified by silica gel column chromatography eluting with ethyl acetate and petroleum ether (20 : 80), furnishing *N*-allyl-5-(dimethylamino)naphthalene-1-sulfonamide (109 mg, 100%) as a fluorescent yellow crystalline solid: *R*_f_ 0.15 (ethyl acetate/petroleum ether 20 : 80); m.p. 62–66 °C (CH_2_Cl_2_); *ν*_max_ (thin film)/cm^–1^; 1644, (s), 1316 (m); ^1^H NMR (400 MHz, CDCl_3_): *δ* 8.56 (d, *J* = 8.6 Hz, 1H,), 8.30 (d, *J* = 8.6 Hz, 1H), 8.26 (d, *J* = 8.6 Hz, 1H), 7.58 (dd, *J* = 7.8, 1.1 Hz, 1H), 7.53 (dd, *J* = 7.8, 1.1 Hz, 1H), 7.2 (d, *J* = 7.6 Hz, 1H), 5.69–5.57 (m, 1H), 5.09 (ddt, *J* = 17.0, 1.2 Hz, 1H), 5.01 (ddt, *J* = 10.2, 1.2 Hz, 1H), 4.75 (t, *J* = 6.2 Hz, 1H), 3.54 (ddd, *J* = 12.2, 6.2, 1.2 Hz, 2H), 2.90 (s, 6H); ^13^C NMR (100 MHz, CDCl_3_): *δ* 151.9, 134.7, 133.0, 130.5, 129.8, 129.6, 129.6, 128.4, 123.2, 118.8, 117.5, 115.3, 45.8, 45.4; HRMS *m*/*z* (ES^+^) [Found; (M + Na)^+^ 313.0891 C_15_H_18_N_2_NaO_2_S requires M^+^, 313.0987]; *m*/*z* 289.10 ([M – H]^–^, 100%); Anal. Calcd for C_15_H_18_N_2_O_2_S: C, 62.0; H, 6.2; N, 9.6. Found: C, 62.0; H, 6.3; N, 9.6.^7^

#### *S*-((5-(Dimethylamino)naphthalen-1-yl)sulfonylaminopropyl) glutathione (**3**)

*N*-Allyl-5-(dimethylamino)naphthalene-1-sulfonamide (100 mg, 0.34 mmol, 1 eq.), l-glutathione (420 mg, 1.36 mmol, 4 eq.), TCEP·HCl (194 mg, 0.68 mmol, 2 eq.) and 2,2-dimethoxyphenyl acetophenone (17 mg, 0.07 mmol, 0.2 eq.) were stirred at RT in THF/H_2_O (1 : 2, 3 mL) in the presence of light (365 nm, 4 × 15 W) for 5 h. After which time the reaction was extracted with CH_2_Cl_2_ (2 × 5 mL). The aqueous layer was lyophilised and the crude material purified by RP C-18 silica gel column chromatography (MeOH/H_2_O 0 : 100, 50 : 50), furnishing *S*-((5-(dimethylamino)naphthalen-1-yl)sulfonylaminopropyl) glutathione 3 (88 mg, 40%) as a hygroscopic yellow solid: *R*_f_ 0.35 (MeOH : H_2_O 50 : 50); [*α*]25D –19.2 (*c* 0.25, H_2_O); *ν*_max_ (PTFE card)/cm^–1^; 3057 (w), 1719 (m), 1647 (m), 1527 (m), 1153 (m); ^1^H NMR (500 MHz, D_2_O): *δ* 8.35 (d, *J* = 8.5 Hz, 1H), 8.15 (d, *J* = 8.5 Hz, 1H), 8.11 (d, *J* = 7.5 Hz, 1H), 7.88–7.55 (m, 2H), 7.26 (d, *J* = 7.5 Hz, 1H), 4.20 (dd, *J* = 8.5, 5.1 Hz, 1H), 3.67–3.52 (m, 3H), 2.85 (t, *J* = 6.8 Hz, 2H), 2.73 (s, 6H), 2.43 (dd, *J* = 13.9, 5.1 Hz, 1H), 2.39–2.28 (m, 3H), 2.07 (t, *J* = 6.8 Hz, 2H), 2.04–1.96 (m, 2H), 1.34 (qn, *J* = 6.8 Hz, 2H); ^13^C NMR (125 MHz; D_2_O): *δ* 176.0, 174.7, 173.9, 171.6, 151.3, 133.9, 130.1, 129.9, 128.9, 128.8, 128.7, 128.3, 123.9, 119.0, 115.9, 54.1, 52.8, 44.8, 43.2, 40.7, 32.5, 31.4, 28.2, 27.6, 26.2; HRMS *m*/*z* (ES^–^) [Found; (M – H)^–^ 596.1852 C_25_H_34_N_5_O_8_S_2_ requires M^–^, 596.1854]; *m*/*z* (ES^–^) 596.2 ([M – H]^–^, 100%); Anal. Calcd for C_25_H_35_N_5_O_8_S_2_: C, 50.2; H, 5.9; N, 11.7. Found 50.1; H, 5.7; N, 11.7.^7^

### General procedure for the synthesis of GSX

The desired alkene (0.34 mmol, 1 eq.), l-glutathione (420 mg, 1.36 mmol, 4 eq.) and 2,2-dimethoxyphenyl acetophenone (17 mg, 0.07 mmol, 0.2 eq.) were stirred at RT in THF/H_2_O (1 : 2, 3 mL) in the presence of light (365 nm, 4 × 15 W) for 5 h. After this time the reaction mixture was filtered, and the solid washed with ethanol and water. The crude solid was further purified by crystallisation from boiling H_2_O and ethanol (×2) unless otherwise stated. [Note: where no precipitate formed, the reaction was washed with dichloromethane (×2) and the aqueous layer lyophilized. In this case, the crude material was purified by RP C-18 silica gel column chromatography]. Data shown for selected examples see ESI† for further details.

***S*-Propyl-3-trimethylsilylglutathione** (**5f**) was isolated as a colourless solid (120 mg, 85%): *R*_f_ 0.16 (MeOH/H_2_O 50 : 50); [*α*]20D –29.0 (*c* 0.5, 2 M NaOH); m.p. 228 °C (dec.) (EtOH/H_2_O); *ν*_max_ (thin film)/cm^–1^; 3372 (m), 3345 (m), 2954 (m), 1672 (s), 1645 (s), 1513 (s) 1432 (m); ^1^H NMR (400 MHz, D_2_O/NaOD, pH 12): *δ* 4.83 (dd, *J* = 9.2, 4.9 Hz, 1H), 3.65 (d, *J*_*AB*_ = 17.3 Hz, 1H), 3.58 (d, *J*_*BA*_ = 17.3 Hz, 1H), 3.11 (dd, *J* = 7.2, 6.0 Hz, 1H), 2.93 (dd, *J* = 14.2, 4.9 Hz, 1H), 2.69 (dd, *J* = 14.2, 9.2 Hz, 1H), 2.50–2.39 (m, 2H), 2.30–2.15 (m, 2H), 1.87–1.63 (m, 2H), 1.50–1.38 (m, 2H), 0.44 (t, *J* = 8.1 Hz, 2H), –0.16 (s, 9H); ^13^C NMR (125 MHz, D_2_O/NaOD, pH 12): *δ* 182.8, 176.6, 176.5, 172.4, 55.9, 53.6, 43.8, 35.7, 32.6, 31.3, 31.2, 24.2, 15.8, –1.8; HRMS *m*/*z* (ES^–^) [Found; (M – H)^–^ 420.1638. C_16_H_30_N_3_O_6_SSi requires M^–^, 420.1360.]; *m*/*z* (ES^–^) 420.1 ([M – H]^–^, 100%); Anal. Calcd for C_16_H_31_N_3_O_6_SSi: C, 45.6; H, 7.4; N, 9.9. Found C, 45.6; H, 7.4; N, 10.1.

**2-(2-Oxopyrolid-1-yl)-*S*-ethyl glutathione** (**5j**) was isolated as a hygroscopic colourless solid (118 mg, 83%): *R*_f_ 0.6 (H_2_O); [*α*]20D –27.4 (*c* 0.5, H_2_O); *ν*_max_ (thin film)/cm^–1^; 3340 (w), 2360 (s), 1740 (s), 1652 (m), 1558 (m); 1540 (m), 1291 (w); ^1^H NMR (400 MHz, D_2_O): *δ* 4.44 (dd, *J* = 8.6, 5.1 Hz, 1H), 3.84 (s, 2H), 3.69 (dd, *J* = 6.4, 6.4 Hz, 1H), 3.42–3.33 (m, 4H), 2.94 (dd, *J* = 14.2, 5.1 Hz, 1H), 2.76 (dd, *J* = 14.2, 8.6 Hz, 1H), 2.67 (t, *J* = 6.5 Hz, 2H), 2.47–2.36 (m, 2H), 2.31 (t, *J* = 7.5 Hz, 2H), 2.09–1.99 (m, 2H), 1.91 (qn, *J* = 7.5 Hz, 2H); ^13^C NMR (100 MHz, D_2_O): *δ* 178.9, 175.0, 173.8, 173.7, 172.9, 54.0, 53.4, 48.2, 41.9, 41.8, 32.9, 31.5, 31.3, 29.2, 26.3, 17.6; HRMS *m*/*z* (ES^–^) [Found; (M – H)^–^ 417.1440. C_16_H_25_N_4_O_7_SNa requires M^–^, 417.1444.]; *m*/*z* (ES^–^) 417.1 ([M – H]^–^, 100%); Anal. Calcd for C_16_H_26_N_4_O_7_S: C, 45.9; H, 6.3; N, 13.4. Found: C, 45.8; H, 6.1; N, 13.3.

**2-(2-Trifluoromethylphenyl)-*S*-ethyl glutathione** (**6b**) was isolated as a colourless solid (64 mg, 39%): *R*_f_ 0.4 (H_2_O/MeOH 50 : 50); [*α*]20D –16.6 (*c* 0.5, 2 M NaOH); *ν*_max_ (thin film)/cm^–1^; 3434 (w), 1746 (m), 1674 (m), 1645 (m), 1514 (s), 1314 (s), 1233 (m), 1114 (m); ^1^H NMR (400 MHz, D_2_O/NaOD, pH 12): *δ* 7.60 (d, *J* = 7.8 Hz, 1H), 7.47 (t, *J* = 7.8 Hz, 1H), 7.37 (d, *J* = 7.8 Hz, 1H), 7.30 (t, *J* = 7.8 Hz, 1H), 4.44 (dd, *J* = 8.9, 4.7 Hz, 1H), 3.66 (d, *J*_*AB*_ = 17.2 Hz, 1H), 3.58 (d, *J*_*BA*_ = 17.2 Hz, 1H), 3.10 (dd, *J* = 7.1, 5.9 Hz, 1H), 3.06–2.94 (m, 3H), 2.83–2.68 (m, 3H), 2.28–2.18 (m, 2H), 1.82–1.62 (m, 2H); ^19^F NMR (125 MHz, D_2_O/NaOD, pH 12); *δ* –58.9; ^13^C NMR (125 MHz, D_2_O/NaOD, pH 12): δ 182.4, 176.3, 176.3, 171.9, 138.4, 132.2, 131.4, 127.8 (d, *J* = 29.3 Hz), 126.8, 126.8 (q, *J* = 5.7 Hz), 124.6 (d, *J* = 270 Hz), 55.5, 52.9, 43.3, 32.9, 32.4, 32.2, 31.9, 30.8; HRMS *m*/*z* (ES^–^) [Found; (M – H)^–^ 478.1273. C_19_H_23_F_3_N_3_O_6_S requires M^–^, 478.1265.]; *m*/*z* (ES^–^) 478.1 ([M – H]^–^, 100%); Anal. Calcd for C_19_H_24_F_3_N_3_O_6_S: C, 47.5; H, 5.0; N, 8.8. Found: C, 47.7; H, 4.9; N, 9.1.
