# Supplementary material for: The photochemical thiol–ene reaction as a versatile method for the synthesis of glutathione S-conjugates targeting the bacterial potassium efflux system Kef
Source: Org Chem Front. 2016 Feb 26;3(4):439–46. doi: 10.1039/c5qo00436e (PMC4819703; doi:10.1039/c5qo00436e)

Electronic Supplementary Material (ESI) for Organic Chemistry Frontiers.
This journal is © the Partner Organisations 2016

Supplementary Information

The photochemical thiol-ene reaction as a versatile method for the synthesis of glutathione S-conjugates targeting the bacterial potassium efflux system Kef

Jess Healy,[b,*] Tim Rasmussen,[c] Samantha Miller,[c] Ian R. Booth,[c] Stuart J. Conway[a,*]

[a]Chemistry Research Laboratory, Mansfield Road, University of Oxford, OX1 3TA, United Kingdom

[b]Department of Pharmaceutical and Biological Chemistry, UCL School of Pharmacy, University College London, 29/39 Brunswick Square, WC1N, 1AX

[c]Institute of Medical Sciences, University of Aberdeen, Foresterhill, Aberdeen, AB25 2ZD, United Kingdom

To whom correspondence should be addressed: *stuart.conway@chem.ox.ac.uk* and *jess.healy@ucl.ac.uk*

Table of Contents:

General experimental……………………………………………….....S2

Experimental procedures…………………………………………….....S3

References…………………………………………………………..…..S8

^{1}H and ^{13}C NMR spectra……………………………………………...S9

General experimental. All reagents were purchased from Sigma Aldrich or Alfa Aesar and were used without further purification. The UV light source was a Philips HB175 Facial Solarium (UVA, 365 nm, P = 4 × 15 W). Reverse phase column chromatography was carried out on Fluka Ltd silica gel 100 C18-reversed phase, under a positive pressure of compressed air. Analytical TLC analysis was performed using Merck 60 RP-18 $F_{254}S$ aluminium-supported thin layer chromatography sheets and visualized using ninhydrin. ^{1}H, ^{13}C and ^{19}F NMR spectra were recorded on a Bruker Avance 400 (400 MHz, 100 MHz) or Bruker Avance III (500 MHz, 125 MHz). ^{1}H and ^{13}C spectra were assigned using 2D NMR experiments including COSY, HSQC and HMBC. Mass spectra were recorded on either a Micromass LCT Premier spectrometer (low resolution) or Bruker MicroTOF spectrometer (high resolution) using electrospray ionization (positive or negative mode, from solutions of methanol, acetonitrile or water). *m/z* values are reported in Dalton and followed by their percentage abundance in parentheses. Melting points were determined using a Kofler hot stage microscope and are uncorrected. Infrared Spectra were obtained either as a thin film of solid or solution in $CHCl_3$. The spectra were recorded on a Bruker Tensor 27 spectrometer. Absorption maxima are reported in wavenumbers (cm^{-1}). Microanalyses were performed by the Elemental Analysis Service, London Metropolitan University. Specific optical rotations were measured using Perkin Elmer Model 241 and 341 polarimeters, in cells with a path length of 1 dm. The light source was maintained at 589 nm. The concentration (*c*) is expressed in g/100 mL (equivalent to g/0.1 dm^3). Specific rotations are denoted $[\alpha]_D^T$ and are given in implied units of 10^{-1} deg cm^2 g^{-1} (T = ambient temperature in °C).

General procedure for the synthesis of GS-X. The desired alkene (0.34 mmol, 1 eq), L-glutathione (420 mg, 1.36 mmol, 4 eq) and 2,2-dimethoxyphenyl acetophenone (17 mg, 0.07 mmol, 0.2 eq) were stirred at RT in THF/H_2O (1:2, 3 mL) in the presence of light (365 nm, 4 × 15 W) for 5 h. After this time the reaction was filtered, and the solid washed with ethanol and water. The crude solid was further purified by crystallization from boiling H_2O and ethanol (×2) unless otherwise stated. [Note: where no precipitate formed, the reaction solution was washed with dichloromethane (×2) and the aqueous layer lyophilized. In this case, the crude material was purified by RP C-18 silica gel column chromatography].

***S*-Hexylglutathione** (**5a**) was isolated as a colourless solid (130 mg, 97%): R_f 0.1 (MeOH/H_2O 50:50); $[\alpha]_D^{20}$ −23.8 (*c* 0.5, 2 M NaOH); m.p. 235 °C (dec.) (EtOH/H_2O); v_{max} (thin film)/cm^{-1}; 3371 (m), 3343 (m), 2954 (m), 1673 (s), 1646 (s), 1514 (s), 1432 (m); ^{1}H NMR (400 MHz, D_2O/NaOD, pH 12): δ 4.42 (dd, *J* = 9.2, 4.6 Hz, 1H), 3.65 (d, J_{AB} = 17.0 Hz, 1H), 3.58 (d, J_{BA} = 17.0 Hz, 1H), 3.11 (dd, *J* = 6.9, 5.9 Hz, 1H), 2.94 (dd, *J* = 14.9, 4.6 Hz, 1H), 2.70 (dd, *J* = 14.9, 9.2 Hz, 1H), 2.44 (td, *J* = 7.2, 1.9 Hz, 2H), 2.28-2.18 (m, 2H), 1.84-1.62 (m, 2H), 1.42 (qn, *J* = 7.2 Hz, 2H), 1.26-1.17 (m, 2H), 1.16-1.09 (m, 4H), 0.71 (t, *J* = 6.9 Hz, 3H); ^{13}C NMR (125 MHz, D_2O/NaOD, pH 12): δ 182.5, 176.3, 176.2, 172.1, 55.5, 53.0, 43.3, 32.9, 32.2, 31.5, 30.8, 30.5, 28.5, 27.5, 21.8, 13.3; HRMS *m/z* (ES$^-$) [Found; (M-H)$^-$ 390.1697. $C_{16}H_{28}N_3O_6S$ requires M$^-$, 390.1704.]; *m/z* (ES$^-$) 390.1 ([M-H]$^-$, 100%); Anal. Calcd. for $C_{16}H_{29}N_3O_6S$: C, 49.0; H, 7.4; N, 10.7. Found: C, 49.0; H, 7.5; N, 10.7. The data are in agreement with the available literature values.[1]

S-Cyclohexylglutathione (**5b**) was isolated as a colourless solid (79 mg, 59 %): R_f 0.36 (MeOH:H_2O 50:50); $[\alpha]_D^{20}$ −21.5 (*c* 0.5, 2 M NaOH); v_{max} (thin film)/cm^{-1}; 3353 (w), 2927 (w), 1675 (s), 1647 (s), 1515 (s), 1312 (m), 1231 (m); ^{1}H NMR (500 MHz, D_2O/NaOD, pH 12): δ 4.42 (dd, *J* = 9.0, 4.2 Hz, 1H), 3.66 (d, J_{AB} = 17.2 Hz, 1H), 3.59 (d, J_{BA} = 17.2 Hz, 1H), 3.12 (dd, *J* = 7.4, 6.0 Hz, 1H), 2.98 (dd, *J* = 14.1, 4.9 Hz, 1H), 2.73 (dd, *J* = 14.1, 9.0 Hz, 1H), 2.71-2.63 (m, 1H), 2.35-2.24 (m, 2H), 1.91-1.70 (m, 4H), 1.67-1.60 (m, 2H), 1.54-1.47 (m, 1H), 1.27-1.09 (m, 5H); ^{13}C NMR (125 MHz, D_2O/NaOD, pH 12): δ 182.5, 176.3, 176.2, 172.0, 55.5, 53.4, 43.4, 43.3, 33.1, 33.0, 32.3, 30.9, 30.8, 25.5, 25.4, 25.2; HRMS *m/z* (ES$^-$) [Found; (M-H)$^-$ 388.1547. $C_{16}H_{26}N_3O_6S$ requires M$^-$, 388.1548.]; *m/z* (ES$^-$) 388.1 ([M-H]$^-$, 100%); Anal. Calcd. for $C_{16}H_{27}N_3O_6S$: C, 49.3; H, 6.9; N, 10.7. Found: C, 49.4; H, 6.9; N, 10.7.

S-Decylglutathione (**5c**) was isolated as a colourless solid (69 mg, 45 %): R_f 0.1 (MeOH/H_2O 50:50); $[\alpha]_D^{20}$ −25.0 (*c* 0.5, 2 M NaOH); m.p. 225 °C (dec.) (EtOH/H_2O); v_{max} (PTFE card)/cm^{-1}; 3277 (m), 2923 (m), 2853 (m), 1582 (s), 1401 (w); ^{1}H NMR (400 MHz, D_2O/NaOD, pH 12): δ 4.37 (dd, *J* = 8.9, 5.0 Hz,

1H), 3.61 (d, J_{AB} = 17.2 Hz, 1H), 3.53 (d, J_{BA} = 17.2 Hz, 1H), 3.11 (dd, J = 6.5, 6.5 Hz, 1H), 2.91-2.82 (m, 1H), 2.68-2.59 (m, 1H), 2.40 (t, J = 6.5 Hz, 2H), 2.27-2.16 (m, 2H), 1.89-1.67 (m, 2H), 1.48-1.35 (m, 2H), 1.26-1.18 (m, 2H), 1.16-1.07 (m, 12H), 0.71 (t, J = 6.5 Hz, 3H); ^{13}C NMR (125 MHz, D_2O/NaOD, pH 12): δ 182.4, 176.2 , 176.0, 172.0, 55.5, 53.2, 43.4, 33.1, 32.2, 31.8, 31.5, 30.9, 29.1, 29.0, 28.95, 28.9, 28.7, 28.2, 22.3, 13.7; HRMS *m/z* (ES^-) [Found; $(M-H)^-$ 446.2343. $C_{20}H_{36}N_3O_6S$ requires M^-, 446.2330.]; *m/z* (ES^-) 446 ($[M-H]^-$, 100%); Anal. Calcd. for $C_{20}H_{37}N_3O_6S$: C, 53.7; H, 8.3; N, 9.4. Found: C, 53.6; H, 8.4; N, 9.4.

S-Dodecylglutathione (**5d**) was isolated as a colourless solid (111 mg, 69%): R_f 0.1 (MeOH/H_2O 50:50); $[\alpha]_D^{20}$ −6.8 (*c* 0.5, 2 M NaOH); m.p. 240 °C (dec.) (EtOH/H_2O); ν_{max} (PTFE card)/cm^{-1}; 3279 (m), 2980 (w), 2920 (w), 2850 (w), 1576 (w), 1556 (w), 1310 (s); ^{1}H NMR (400 MHz, D_2O /NaOD, pH 12): δ 4.31 (dd, J = 8.3, 5.8 Hz, 1H), 3.61 (d, J_{AB} = 17.1 Hz, 1H), 3.44 (d, J_{BA} = 17.1 Hz, 1H), 3.03 (dd, J = 6.8, 6.8 Hz, 1H), 2.77 (dd, J = 13.5, 5.8 Hz, 1H), 2.67-2.50 (m, 1H), 2.33 (q, J = 7.0 Hz, 2H), 2.19-2.08 (m, 2H), 1.75-1.56 (m, 2H), 1.38-1.27 (m, 2H), 1.19-1.10 (m, 2H), 1.08-0.97 (m, 16H), 0.62 (t, J = 7.0 Hz, 3H); ^{13}C NMR (125 MHz, D_2O/NaOD, pH 12): δ 182.3, 176.2, 175.7, 171.9, 55.6, 53.2, 43.5, 33.2, 32.0, 31.9, 30.9, 29.7, 29.7, 29.6, 29.4, 29.3, 29.3, 28.7, 22.6, 13.9; HRMS *m/z* (ES^-) [Found; $(M-H)^-$ 474.2642. $C_{22}H_{40}N_3O_7S$ requires M, 474.2643.]; *m/z* (ES^-) 474 ($[M-H]^-$, 100%).

S-Hexadecylglutathione (**5e**) was isolated as a colourless solid (123 mg, 70%): R_f 0.1 (MeOH/H_2O 50:50); $[\alpha]_D^{20}$ −10.0 (*c* 0.5, 2 M NaOH); m.p. 200 °C (dec.) (EtOH/H_2O); ν_{max} (PTFE card)/cm^{-1}; 3542 (m), 2960 (w), 2920 (w), 2851 (w), 1494 (w), 1309 (s); ^{1}H NMR (400 MHz, NaOD/D_2O, pH 12): δ 4.39 (dd, J = 7.0, 7.0 Hz, 1H), 3.69 (d, J_{AB} = 17.3 Hz, 1H), 3.52 (d, J_{BA} = 17.3 Hz, 1H), 3.11 (dd, J = 6.4, 6.4 Hz, 1H), 2.91-2.80 (m, 1H), 2.75-2.57 (m, 1H), 2.38 (t, J = 7.0 Hz, 2H), 2.27-2.16 (m, 2H), 1.83-1.63 (m, 2H), 1.48-1.35 (m, 2H), 1.26-1.18 (m, 2H), 1.16-1.07 (m, 24H), 0.71 (J = 6.7 Hz, 3H); ^{13}C NMR (125 MHz, D_2O/NaOD, pH 12): δ 182.6, 176.6, 176.0, 171.9, 56.03, 53.2, 43.80, 33.3, 32.2, 32.1, 31.9, 30.9, 29.9, 29.8, 29.5, 29.4, 28.8, 22.6, 13.9; HRMS *m/z* (ES^-) [Found; $(M-H)^-$ 530.3296. $C_{26}H_{48}N_3O_6S$ requires M^-, 530.3264.]; *m/z* (ES^-) 530 ($[M-H]^-$, 100%).

S-3, 3-Dimethylbutylglutathione (**5g**) was isolated as a colourless solid (88 mg, 66%): R_f 0.27 (MeOH/H_2O 50:50); $[\alpha]_D^{20}$ −30.0 (*c* 0.5, 2 M NaOH); m.p. 235 °C (dec.) (EtOH/H_2O); ν_{max} (thin film)/cm^{-1}; 3375 (m), 3348 (m), 2955 (m), 1673 (s), 1645 (s), 1513 (s), 1433 (m), 1248 (m); ^{1}H NMR (400 MHz, D_2O/NaOD, pH 12): δ 4.44 (dd, J = 9.1, 4.7 Hz, 1H), 3.67 (d, J_{AB} = 17.1 Hz, 1H), 3.61 (d, J_{BA} = 17.1 Hz, 1H), 3.13 (dd, J = 6.6, 6.6 Hz, 1H), 2.97 (dd, J = 14.2, 4.7 Hz, 1H), 2.73 (dd, J = 14.2, 9.1 Hz, 1H), 2.45-2.40 (m, 2H), 2.20-

2.31 (m, 2H), 1.85-1.63 (m, 2H), 1.39-1.24 (m, 2H), 0.76 (s, 9H); ^{13}C NMR (125 MHz, D_2O/NaOD, pH 12): δ 182.5, 176.3, 176.2, 172.1, 55.5, 52.9, 43.3, 43.1, 32.7, 32.2, 30.8, 29.8, 28.2, 27.2; HRMS *m/z* (ES^-) [Found; $(M-H)^-$ 390.1695. $C_{16}H_{28}N_3O_6S$ requires M^-, 390.1704.]; *m/z* (ES^-) 390.1 ($[M-H]^-$, 100%); Anal. Calcd. for $C_{16}H_{29}N_3O_6S$: C, 49.1; H, 7.5; N, 10.7. Found C, 49.1; H, 7.4; N, 10.7.

***S*-Propyl-3-hydroxyl glutathione** (**5h**) was purified by RP C-18 silica gel column chromatography (H_2O) furnishing a hygroscopic colourless solid (105 mg, 85%): R_f 0.7 (H_2O); $[\alpha]_D^{20}$ −47.8 (*c* 0.5, H_2O); ν_{max} (thin film)/cm^{-1}; 3613 (w), 3447 (w), 2365 (m), 1644 (s), 1525 (s), 1407 (m); ^{1}H NMR (400 MHz, D_2O): δ 4.44 (dd, *J* = 8.6, 4.9 Hz, 1H), 3.84 (s, 2H), 3.69 (dd, *J* = 6.4, 6.4 Hz, 1H), 3.52 (t, *J* = 6.3 Hz, 2H), 2.92 (dd, *J* = 14.2, 4.9 Hz, 1H), 2.74 (dd, *J* = 14.2, 8.7 Hz, 1H), 2.51 (t, *J* = 7.3 Hz, 2H), 2.45-2.33 (m, 2H), 2.03 (dd, *J* = 7.6, 6.4 Hz, 2H), 1.72-1.63 (m, 2H); ^{13}C NMR (100 MHz, D_2O): δ 175.1, 173.9, 173.8, 173.1, 60.4, 54.0, 53.4, 41.8, 33.0, 31.4, 31.3, 28.3, 26.3; HRMS *m/z* (ES^-) [Found; $(M-H)^-$ 364.1174. $C_{13}H_{22}N_3O_7S$ requires M^-, 364.1184.]; *m/z* (ES^-) 364.1 ($[M-H]^-$, 100%); Anal. Calcd. for $C_{13}H_{23}N_3O_7S$: C, 42.7; H, 6.3; N, 11.5. Found: C, 42.8; H, 6.3; N, 11.6. The data are in agreement with the available literature values.[2]

S-Propyl-3-amino glutathione (**5i**) was purified by RP C-18 silica gel column chromatography (H_2O) furnishing a hygroscopic colourless solid (101 mg, 80%): R_f 0.7 (H_2O); $[\alpha]_D^{20}$ −27.0 (*c* 0.5, 2 M NaOH); ν_{max} (thin film)/cm^{-1}; 2969 (w), 1738 (s), 1366 (s), 1217 (s); ^{1}H NMR (500 MHz, D_2O): δ 4.50 (dd, *J* = 8.7, 5.0 Hz, 1H), 3.70-3.66 (m, 3H), 3.03-2.97 (m, 3H), 2.80 (dd, *J* = 14.1, 8.7 Hz, 1H), 2.64-2.53 (m, 2H), 2.50-2.40 (m, 2H), 2.10-2.04 (m, 2H), 1.91-1.82 (m, 2H); ^{13}C NMR (125 MHz, D_2O): δ 176.1, 174.8, 174.1, 171.9, 54.0, 52.9, 43.3, 38.2, 32.6, 31.4, 28.1, 26.4, 26.1; HRMS *m/z* (ES^+) [Found; $(M+H)^+$ 365.1481. $C_{13}H_{25}N_4O_6S$ requires M^+, 365.1489.]; *m/z* (ES^+) 365.1 ($[M+H]^+$, 100%).

S-(6,6-Dimethylbicyclo[3.1.1]heptan-2-yl)methyl glutathione (**5k**) was purified by RP C-18 silica gel column chromatography (H_2O) furnishing a colourless solid (1 mg, 0.006%): R_f 0.25 (MeOH:H_2O 50:50); ν_{max} (thin film)/cm^{-1}; 3592 (w), 1738 (s), 1641 (m), 1437 (m), 1370 (s), 1229 (s), 1217 (s); ^{1}H NMR (400 MHz, D_2O): δ 4.43 (dd, *J* = 8.9, 5.0 Hz, 1H), 3.77 (s, 2H), 3.65 (dd, *J* = 6.3, 6.3 Hz, 1H), 2.92 (dd, *J* = 14.4, 5.0 Hz, 1H), 2.72 (dd, *J* = 14.4, 8.9 Hz, 1H), 2.62 (m, 2H), 2.46-2.34 (m, 2H), 2.26-2.19 (m, 1H), 2.11-1.92 (m, 3H), 1.92-1.72 (m, 4H), 1.42-1.29 (m, 1H), 1.05 (s, 3H), 0.85 (s, 3H); HRMS *m/z* (ES^+) [Found; $(M+Na)^+$ 466.1966. $C_{20}H_{33}N_3O_6SNa$ requires M^+, 466.1982.]; *m/z* (ES^+) 466.1 ($[M+Na]^+$, 100%).

2-(4-Nitrophenyl)-S-ethyl glutathione (**6a**) was isolated as a pale yellow solid (20 mg, 13%); R_f 0.4 (H_2O/MeOH 50:50); $[\alpha]_D^{20}$ −18.1 (*c* 0.5, H_2O); m.p. 200 °C (dec.) (EtOH/H_2O) (lit. 201-202 °C); ν_{max} (thin film)/cm^{-1}; 3546 (w), 1646 (s), 1518 (s), 1346 (s); ^{1}H NMR (400 MHz, D_2O): δ 8.04 (d, *J* = 8.5 Hz, 2H), 7.34 (d, *J* = 8.5 Hz, 2H), 4.36 (dd, *J* = 8.7, 5.2 Hz, 1H), 3.80 (s, 2H), 3.66 (dd, *J* = 6.4, 6.4 Hz, 1H), 2.94-2.75 (m, 5H), 2.68 (dd, *J* = 14.0, 8.7 Hz, 1H), 2.42-2.31 (m, 2H), 2.06-1.96 (m, 2H); HRMS *m/z* (ES^-) [Found; $(M-H)^-$ 455.1246. $C_{18}H_{23}N_4O_8S$ requires M^-, 455.1242.]; *m/z* (ES^-) 455.1 ($[M-H]^-$, 100%). The data are in agreement with the available literature values.[1]

2-(4-Fluorophenyl)-S-ethyl glutathione (**6c**) was isolated as a colourless solid (70 mg, 48%): R_f 0.36 (MeOH:H_2O 50:50); $[\alpha]_D^{20}$ −19.2 (*c* 0.5 in 2 M NaOH); m.p. 220 °C (dec.) (EtOH/H_2O); ν_{max} (thin film)/cm^{-1}; 3344 (w), 2945 (w), 1672 (m), 1645 (s), 1509 (s), 1432 (s), 1352 (w), 1312 (w), 1222 (m); ^{1}H NMR (500 MHz, D_2O/NaOD, pH 12): δ 7.18-7.11 (m, 2H), 6.98-6.90 (m, 2H), 4.39 (dd, *J* = 9.2, 4.8 Hz, 1H), 3.64 (d, J_{AB}= 17.3 Hz, 1H), 3.62 (d, J_{BA}= 17.3 Hz, 1H), 3.10 (dd, *J* = 7.3, 6.1 Hz, 1H), 2.90 (dd, *J* = 14.1, 4.8 Hz, 1H), 2.80-2.72 (m, 4H), 2.67 (dd, *J* = 14.1, 9.2 Hz, 1H), 2.22 (ddd, *J* = 10.0, 7.0, 2.5 Hz, 2H), 1.82-1.61 (m, 2H); ^{13}C NMR (125 MHz, D_2O/NaOD, pH 12): δ 182.5, 176.3, 176.2, 172.0, 161.5 (d, *J* = 235 Hz), 136.1 (d, *J* = 2.9 Hz), 130.2 (d, *J* = 8.5 Hz), 115.1 (d, *J* = 21.5), 55.6, 52.9, 43.3, 33.9, 33.0, 32.9, 32.2, 30.8; ^{19}F NMR (125 MHz, D_2O/NaOD, pH 12); δ −117.0; HRMS *m/z* (ES^-) [Found; $(M-H)^-$ 428.1296. $C_{18}H_{23}FN_3O_6SNa$ requires M^-, 428.1297.]; *m/z* (ES^-) 428.1 ($[M-H]^-$, 100%); Anal. Calcd. for $C_{18}H_{24}FN_3O_6S$: C, 50.3; H, 5.6; N, 9.8. Found: C, 50.4; H, 5.6; N, 9.8.

2-(4-Fluorophenyl)-S-propyl glutathione (**6d**) was isolated as a colourless crystalline solid (135 mg, 90%): R_f 0.28 (MeOH:H_2O 50:50); m.p. 170-173 °C (EtOH/H_2O); $[\alpha]_D^{20}$ −6.2 (*c* 0.5 in 2 M NaOH); ν_{max} (thin film)/cm^{-1}; 3368 (w), 3344 (w), 2941 (w), 2289 (m), 1672 (m), 1645 (s), 1509 (s), 1433 (m), 1349 (w), 1217 (s); ^{1}H NMR (500 MHz, D_2O/NaOD, pH12): δ 7.12 (d, *J* = 5.8 Hz, 1H), 7.10 (dd, *J* = 6.7, 2.1 Hz, 1H), 6.95 (dd, *J* = 6.7, 2.1 Hz, 1H), 6.93 (dd, *J* = 6.7, 2.1 Hz, 1H), 4.40 (dd, *J* = 9.0, 4.9 Hz, 1H), 3.67 (d, J_{AB} = 17.2 Hz, 1H), 3.59 (d, J_{BA} = 17.2 Hz, 1H), 3.20 (dd, *J* = 6.2, 6.2 Hz, 1H), 2.93 (dd, *J* = 14.4, 4.9 Hz, 1H), 2.71 (dd, *J* = 14.4, 9.0 Hz, 1H), 2.55 (t, *J* = 7.4 Hz, 2H), 2.41 (td, *J* = 7.2, 2.4 Hz, 2H), 2.27 (dd, *J* = 7.9, 7.9 Hz, 2H), 1.89-1.68 (m, 4H); ^{13}C NMR (125 MHz, D_2O/NaOD, pH 12): δ 181.8, 176.5, 176.4, 172.3, 161.5 (d, *J* = 240 Hz), 137.9, 130.4 (d, *J* = 8.0 Hz), 115.4 (d, *J* = 21 Hz), 55.7, 53.4, 43.7, 33.4, 33.2, 32.5, 31.1, 30.8, 30.6; ^{19}F NMR (100 MHz, D_2O/NaOD pH 12): δ −118.1; HRMS *m/z* (ES^+) [Found; $(M+Na)^+$ 466.1420. $C_{19}H_{26}FN_3NaO_6SNa$ requires M^+, 466.1419.]; *m/z* (ES^-) 442.1 ($[M-H]^-$, 100%); Anal. Calcd. for $C_{19}H_{26}FN_3O_6S$: C, 51.5; H, 5.9; N, 9.5. Found: C, 51.5; H, 5.9; N, 9.4.

2-(Phenylsulfonyl)-S-ethyl glutathione (**6e)** was purified by RP C-18 silica gel column chromatography (H_2O) furnishing a hygroscopic colourless solid (73 mg, 45%): R_f 0.54 (MeOH:H_2O 50:50); $[\alpha]_D^{20}$ −15.0 (*c* 0.5, H_2O); ν_{max} (thin film)/cm^{-1}; 2229 (m), 2180 (m), 1741 (s), 1367 (m), 1216 (m); ^{1}H NMR (500 MHz, D_2O): δ 7.82 (dd, *J* = 8.7, 1.5 Hz, 2H), 7.69 (tt, *J* = 7.4, 1.5 Hz, 1H), 7.64 (tt, *J* = 7.4, 1.5 Hz, 2H), 4.36 (dd, *J* = 8.3, 5.3 Hz, 1H), 3.81 (s, 2H), 3.61 (dd, *J* = 6.4, 6.4 Hz, 1H), 3.53 (dd, *J* = 9.4, 6.1 Hz, 2H), 2.22 (dd, *J* = 14.1, 5.3 Hz, 1H), 2.75-2.66 (m, 3H), 2.37 (ddd, *J* = 7.6, 7.6, 3.2 Hz, 2H), 2.09-2.01 (m, 2H); ^{13}C NMR (125 MHz, D_2O): δ 174.7, 173.7, 173.6, 172.2, 136.5, 135.0, 129.7, 127.8, 54.9, 53.8, 52.7, 41.6, 32.7, 31.2, 25.9, 24.0; HRMS *m/z* (ES$^-$) [Found; (M-H)$^-$ 474.1020. $C_{18}H_{24}N_3O_8S_2$ requires M$^-$, 474.1010.]; *m/z* (ES$^-$) 474.1 ([M-H]$^-$, 100%); Anal. Calcd. for $C_{18}H_{25}N_3O_8S_2$: C, 45.5; H, 5.3; N, 8.8. Found: C, 45.5; H, 5.2; N, 8.8. The data are in good agreement with the available literature data.[3]

2-(4-Methoxyphenyl)-S-ethyl glutathione (**6g**) was isolated as a colourless solid (125 mg, 84%): R_f 0.45 (MeOH:H_2O 50:50); $[\alpha]_D^{20}$ −22.6 (*c* 0.5, 2 M NaOH); ν_{max} (thin film)/cm^{-1}; 3349 (w), 1644 (m), 1512 (s), 1245 (m); ^{1}H NMR (400 MHz, D_2O/NaOD, pH 12): δ 7.12 (d, *J* = 8.3 Hz, 2H), 6.84 (d, *J* = 8.3 Hz, 2H), 4.39 (dd, *J* = 9.1, 4.9 Hz, 1H), 3.69 (s, 3H), 3.63 (d, J_{AB} = 17.2 Hz, 1H), 3.58 (d, J_{BA} = 17.2 Hz, 1H), 3.10 (dd, *J* = 7.1, 5.9 Hz, 1H), 2.90 (dd, *J* = 14.2, 4.9 Hz, 1H), 2.77-2.72 (m, 4H), 2.67 (dd, *J* = 14.2, 9.1 Hz, 1H), 2.27-2.19 (m, 2H), 1.82-1.62 (m, 2H); ^{13}C NMR (125 MHz, D_2O/NaOD, pH 12): δ 182.4, 176.3, 176.2, 157.3, 133.1, 129.9, 114.0, 55.5, 55.4, 52.9, 43.3, 33.9, 33.1, 32.9, 32.2, 30.8; HRMS *m/z* (ES$^-$) [Found; (M-H)$^-$ 440.1503. $C_{19}H_{26}N_3O_7S$ requires M$^-$, 440.1497.]; *m/z* (ES$^-$) 440.1 ([M-H]$^-$, 100%); Anal. Calcd. for $C_{19}H_{27}N_3O_7S$: C, 51.7; H, 6.2; N, 9.5. Found: C, 51.7; H, 6.1; N, 9.6.

2-(Phenyl)-S-ethyl glutathione (**6f**) was isolated as a colourless solid (75 mg, 53%); R_f 0.3 (MeOH/H_2O 50:50); $[\alpha]_D^{20}$ −21.0 (*c* 0.5, 2 M NaOH); m.p. 210 °C (dec.) (EtOH/H_2O) (lit. 199-200 °C); ν_{max} (thin film)/cm^{-1}; 3346 (m), 2951 (w), 1672 (s), 1645 (s), 1512 (s), 1432 (m); ^{1}H NMR (400 MHz, D_2O/NaOD, pH 12): δ 7.25-7.17 (m, 2H), 7.16-7.09 (m, 3H), 4.36 (dd, *J* = 9.0, 4.8 Hz, 1H), 3.62 (d, J_{AB} = 17.2 Hz, 1H), 3.54 (d, J_{BA} = 17.2 Hz, 1H), 3.06 (dd, *J* = 6.2, 6.2 Hz, 1H), 2.88 (dd, *J* = 14.2, 4.8 Hz, 1H), 2.78-2.70 (m, 4H), 2.64 (dd, *J* = 14.2, 9.0 Hz, 1H), 2.18 (dd, *J* = 7.3, 7.3 Hz, 2H), 1.79-1.57 (m, 2H); ^{13}C NMR (125 MHz, D_2O/NaOD, pH 12): δ 182.5, 176.3, 176.2, 172.0, 140.4, 128.7, 126.5, 55.5, 53.6, 43.4, 34.8, 32.9, 32.2, 30.8; HRMS *m/z* (ES$^-$) [Found; (M-H)$^-$ 410.1392. $C_{18}H_{24}N_3O_6S$ requires M$^-$, 410.1391.]; *m/z* (ES$^-$) 410.1 ([M-H]$^-$, 100%); Anal. Calcd. for $C_{18}H_{25}N_3O_6S$: C, 52.5; H, 6.1; N, 10.2. Found: C, 52.6; H, 6.1; N, 10.3. The data are in good agreement with the available literature data.[1]

(1) Vince, R.; Vince, R.; Daluge, S.; Daluge, S.; Wadd, W. B.; Wadd, W. B. *J. Med. Chem.* **1971**, *14*, 402–404.
(2) Jin, L.; Baillie, T. A. *Chem. Res. Toxicol.* **1997**, *10*, 318–327.
(3) Horner, L.; Lindel, H. *Phosphorous and Sulfur and the Related Elements* **1983**, *15*, 1–8.

N-Allyl-5-(dimethylamino)naphthalene sulfonamide (**2**) – ^{1}H NMR

Current Data Parameters
NAME Sep25–2009–19
EXPNO 1
PROCNO 1

F2 – Acquisition Parameters
Date_ 20090925
Time 16.33
INSTRUM av400
PROBHD 5 mm QNP 1H/13
PULPROG zg60
TD 65536
SOLVENT CDCl3
NS 16
DS 2
SWH 8278.146 Hz
FIDRES 0.126314 Hz
AQ 3.9584243 sec
RG 181
DW 60.400 usec
DE 7.50 usec
TE 300.0 K
D1 1.00000000 sec

======== CHANNEL f1 ========
NUC1 1H
P1 9.00 usec
PL1 0.00 dB
SFO1 400.2024714 MHz

F2 – Processing parameters
SI 32768
SF 400.2000028 MHz
WDW EM
SSB 0
LB 0.30 Hz
GB 0
PC 1.00

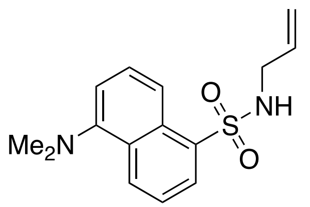

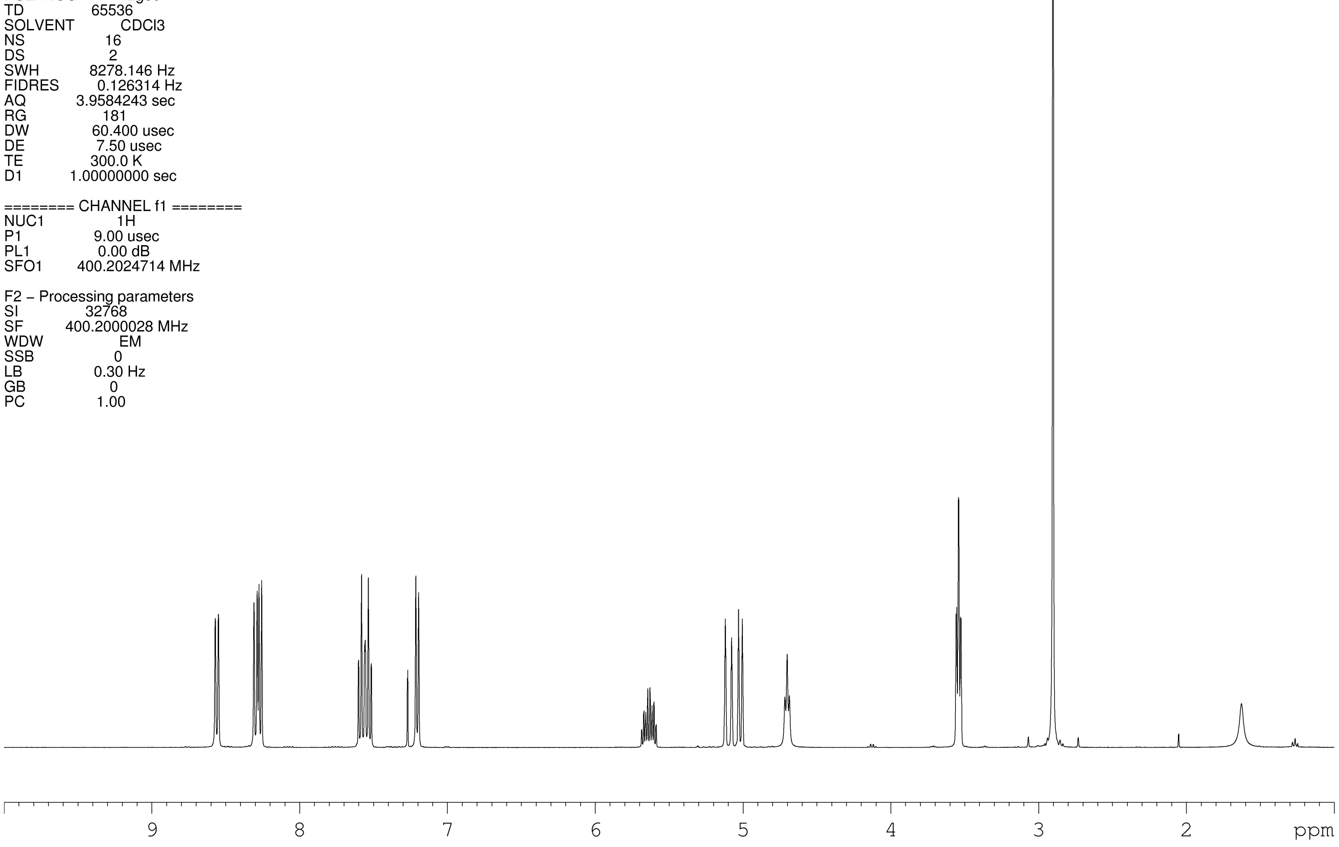

N-Allyl-5-(dimethylamino)naphthalene sulfonamide (**2**) – ^{13}C NMR

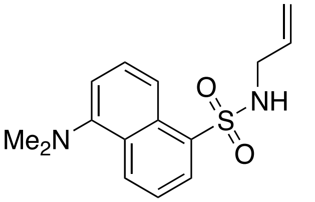

Current Data Parameters
NAME May29–2012–13
EXPNO 2
PROCNO 1

F2 – Acquisition Parameters
Date_ 20120530
Time 13.51
INSTRUM av400
PROBHD 5 mm QNP 1H/13
PULPROG zgpg30
TD 32768
SOLVENT CDCl3
NS 256
DS 4
SWH 26178.010 Hz
FIDRES 0.798889 Hz
AQ 0.6259188 sec
RG 32768
DW 19.100 usec
DE 7.50 usec
TE 300.0 K
D1 1.00000000 sec
D11 0.03000000 sec
TD0 1

======== CHANNEL f1 ========
NUC1 13C
P1 9.50 usec
PL1 0.00 dB
SFO1 100.6403931 MHz

======== CHANNEL f2 ========
CPDPRG2 waltz16
NUC2 1H
PCPD2 80.00 usec
PL2 0.00 dB
PL12 19.00 dB
PL13 25.00 dB
SFO2 400.2016008 MHz

F2 – Processing parameters
SI 32768
SF 100.6303718 MHz
WDW EM
SSB 0
LB 1.00 Hz
GB 0
PC 1.40

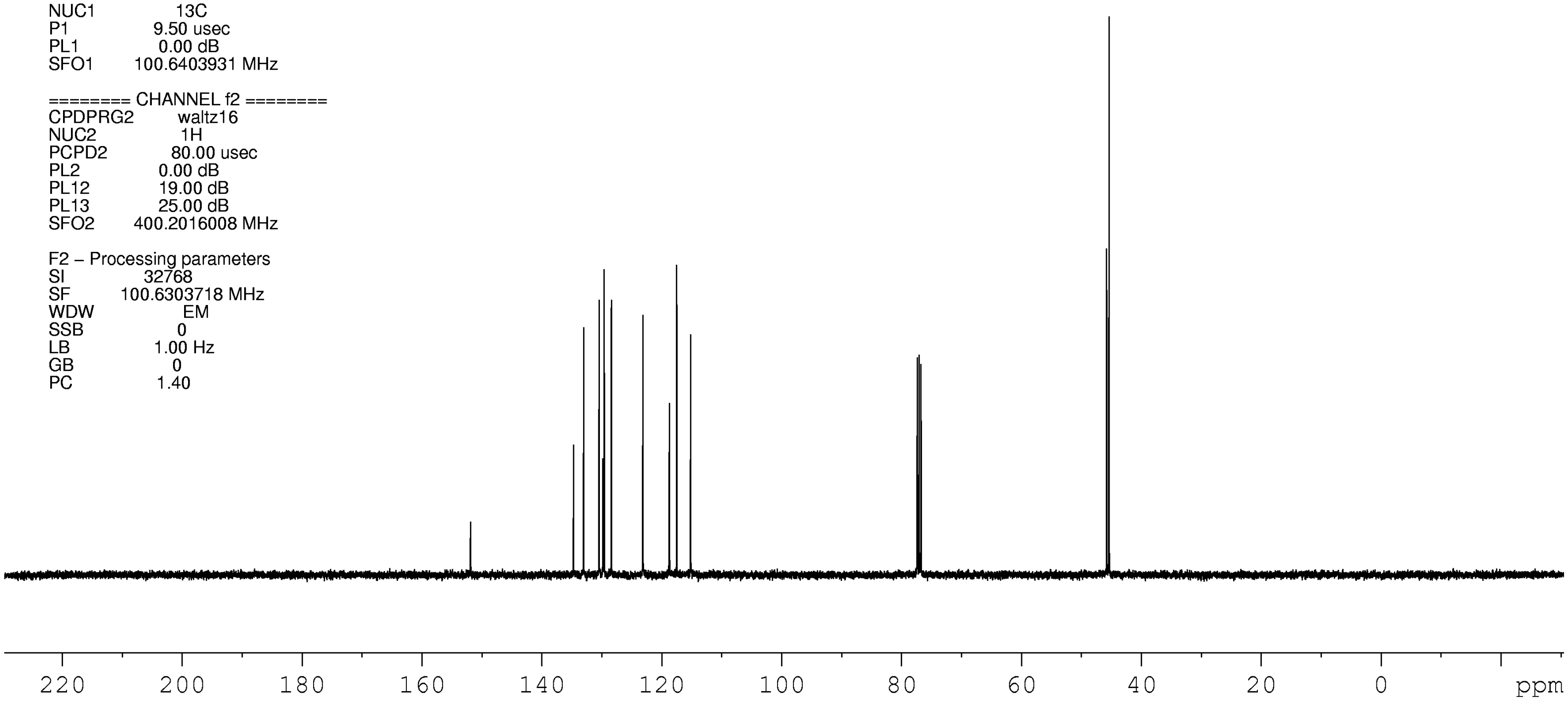

S-(*N*-Propyl-5-(dimethylamino)naphthalene-1-sulfonamide glutathione (**3**) – ^{1}H NMR

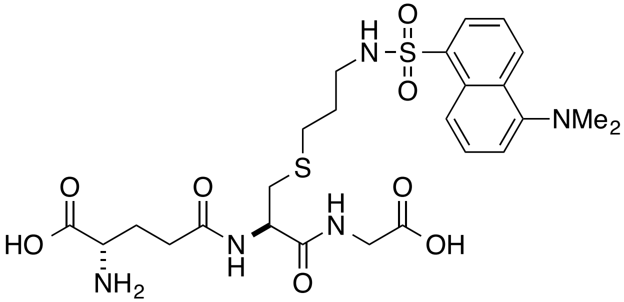

Current Data Parameters
NAME jh77191902
EXPNO 1
PROCNO 1

F2 - Acquisition Parameters
Date_ 20100219
Time 12.38
INSTRUM drx500
PROBHD 5 mm PABBO BB/
PULPROG zg30
TD 65536
SOLVENT D2O
NS 16
DS 2
SWH 10330.578 Hz
FIDRES 0.157632 Hz
AQ 3.1720407 sec
RG 181
DW 48.400 usec
DE 6.00 usec
TE 298.1 K
D1 1.00000000 sec
TD0 1

======== CHANNEL f1 ========
NUC1 1H
P1 12.50 usec
PL1 0.00 dB
SFO1 500.1330885 MHz

F2 - Processing parameters
SI 32768
SF 500.1300000 MHz
WDW EM
SSB 0
LB 0.30 Hz
GB 0
PC 1.00

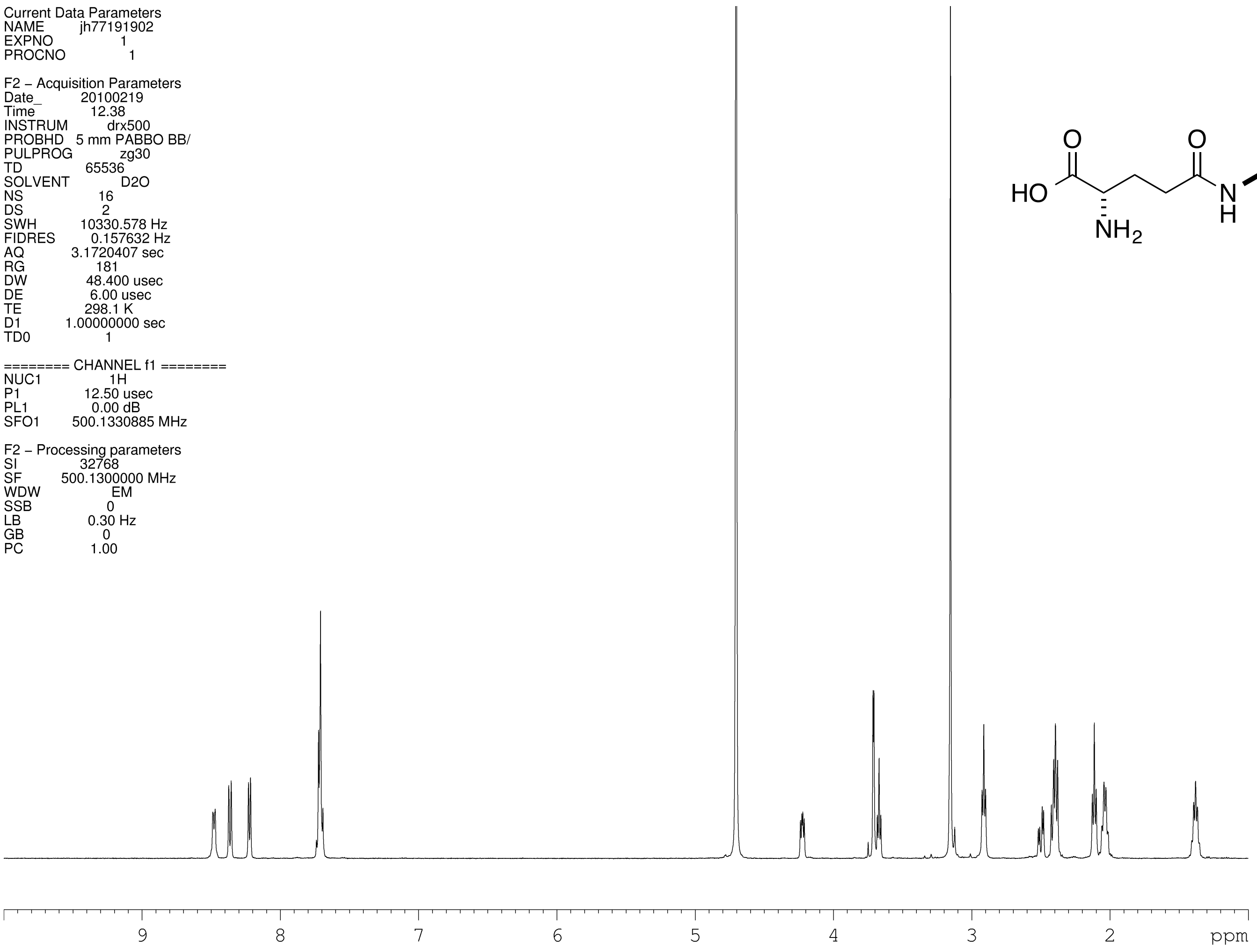

S-(*N*-Propyl-5-(dimethylamino)naphthalene-1-sulfonamide glutathione (**3**) – ^{13}C NMR

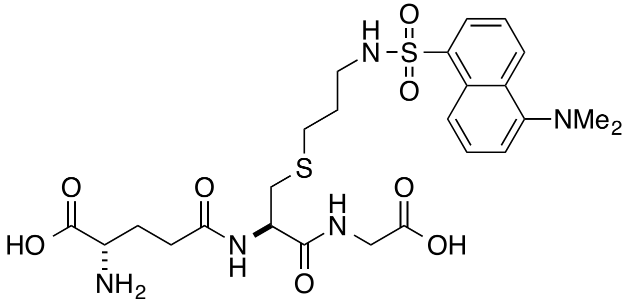

Current Data Parameters
NAME jh77191902
EXPNO 2
PROCNO 1

F2 – Acquisition Parameters
Date_ 20100219
Time 14.25
INSTRUM drx500
PROBHD 5 mm PABBO BB/
PULPROG zgpg30
TD 65536
SOLVENT D2O
NS 2000
DS 4
SWH 30030.029 Hz
FIDRES 0.458222 Hz
AQ 1.0912410 sec
RG 4597.6
DW 16.650 usec
DE 6.00 usec
TE 298.0 K
D1 2.00000000 sec
d11 0.03000000 sec
DELTA 1.89999998 sec
TD0 1

======== CHANNEL f1 ========
NUC1 13C
P1 9.70 usec
PL1 6.00 dB
SFO1 125.7703643 MHz

======== CHANNEL f2 ========
CPDPRG2 waltz16
NUC2 1H
PCPD2 100.00 usec
PL2 0.00 dB
PL12 19.50 dB
PL13 22.50 dB
SFO2 500.1320005 MHz

F2 – Processing parameters
SI 32768
SF 125.7577890 MHz
WDW EM
SSB 0
LB 1.00 Hz
GB 0
PC 1.40

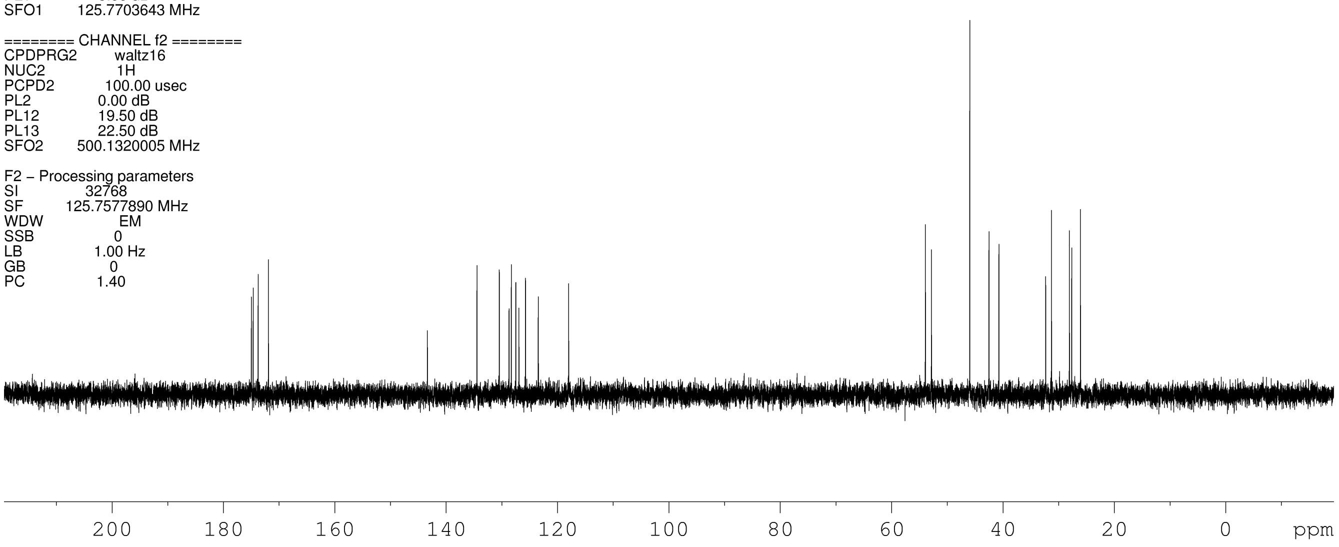

S-Hexylglutathione (**5a**) – ^{1}H NMR

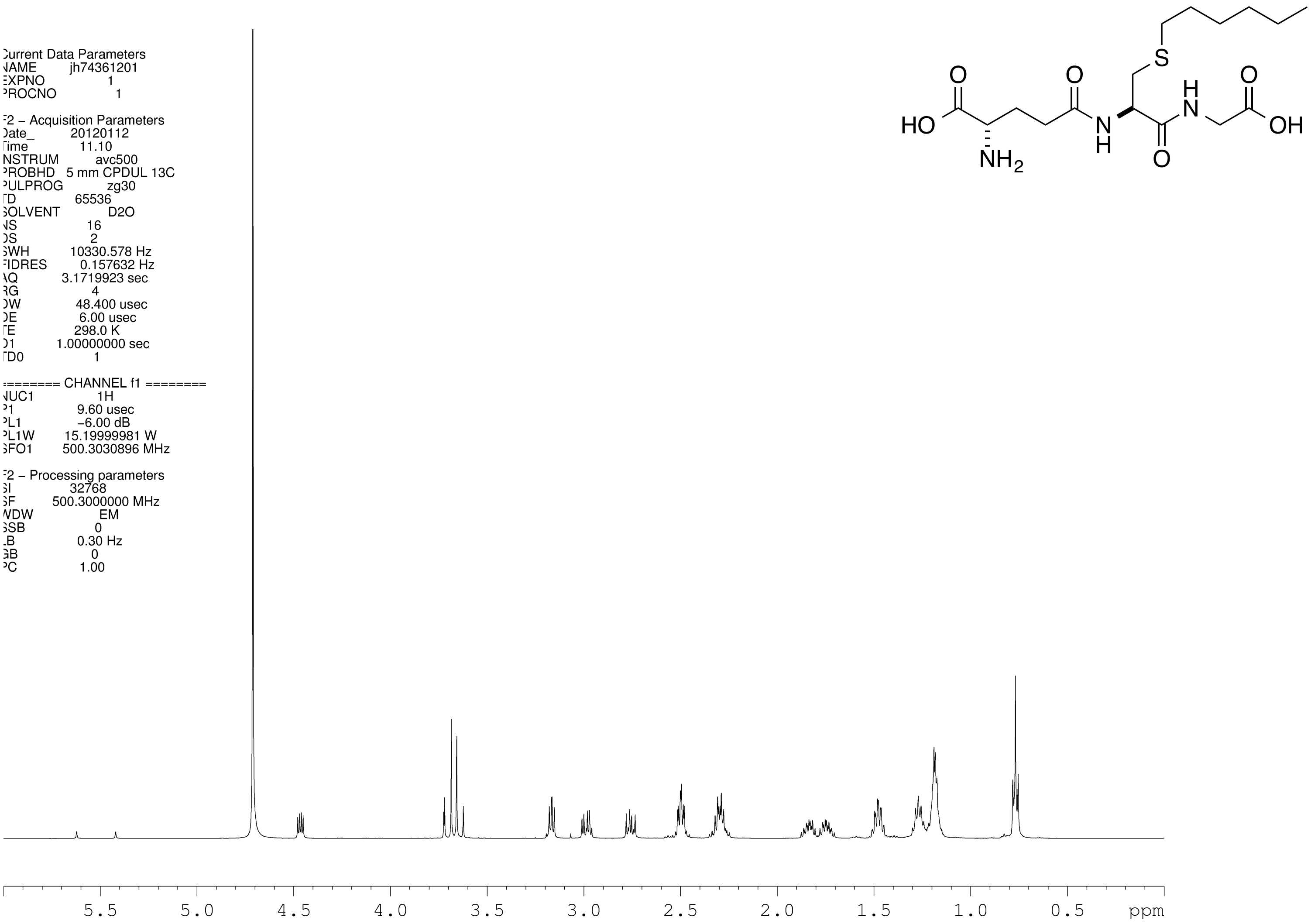

S-Hexylglutathione (**5a**) – ^{13}C NMR

Current Data Parameters
NAME jh74361201
EXPNO 5
PROCNO 1

F2 – Acquisition Parameters
Date_ 20120112
Time 15.18
INSTRUM avc500
PROBHD 5 mm CPDUL 13C
PULPROG zgpg30
TD 65536
SOLVENT D2O
NS 853
DS 2
SWH 31250.000 Hz
FIDRES 0.476837 Hz
AQ 1.0486259 sec
RG 1820
DW 16.000 usec
DE 20.00 usec
TE 298.0 K
D1 2.00000000 sec
D11 0.03000000 sec
TD0 1

======== CHANNEL f1 ========
NUC1 13C
P1 10.00 usec
PL1 –4.40 dB
PL1W 28.15752029 W
SFO1 125.8131151 MHz

======== CHANNEL f2 ========
CPDPRG2 waltz16
NUC2 1H
PCPD2 80.00 usec
PL2 –6.00 dB
PL12 12.42 dB
PL13 18.42 dB
PL2W 15.19999981 W
PL12W 0.21869738 W
PL13W 0.05493430 W
SFO2 500.3020012 MHz

F2 – Processing parameters
SI 32768
SF 125.8005350 MHz
WDW EM
SSB 0
LB 1.00 Hz
GB 0
PC 1.40

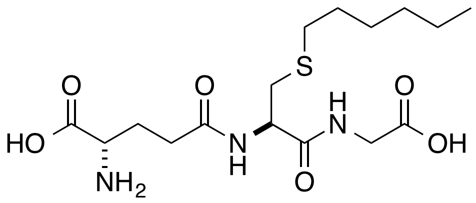

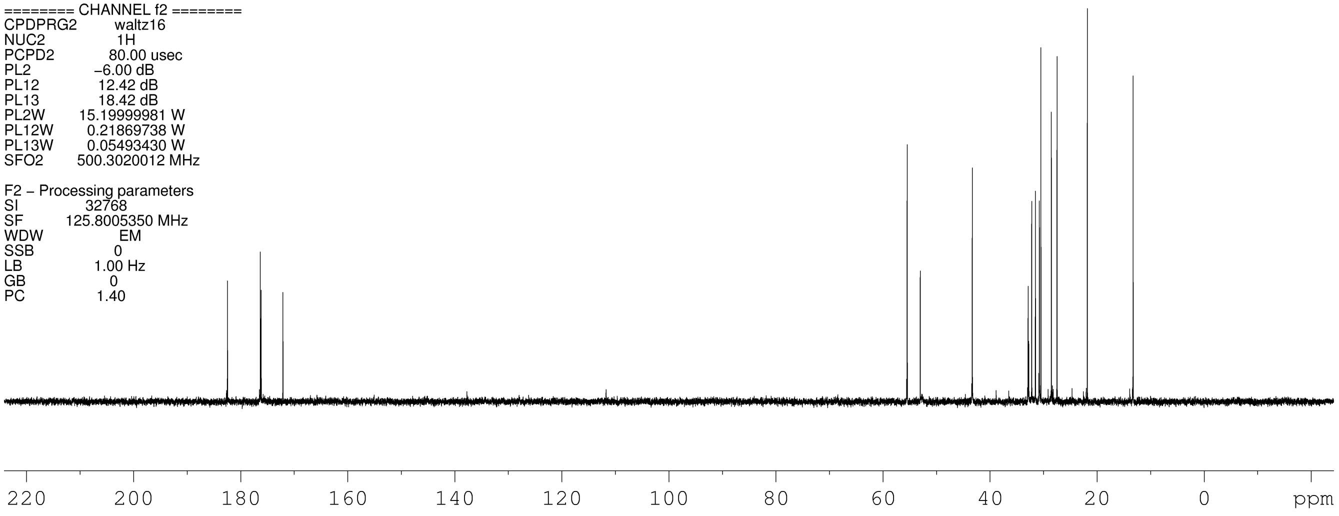

S-Cyclohexylglutathione (**5b**) – ^{1}H NMR

Current Data Parameters
NAME jh81302102
EXPNO 1
PROCNO 1

F2 – Acquisition Parameters
Date_ 20120221
Time 9.12
INSTRUM avc500
PROBHD 5 mm CPDUL 13C
PULPROG zg30
TD 65536
SOLVENT D2O
NS 16
DS 2
SWH 10330.578 Hz
FIDRES 0.157632 Hz
AQ 3.1719923 sec
RG 4
DW 48.400 usec
DE 6.00 usec
TE 298.0 K
D1 1.00000000 sec
TD0 1

======== CHANNEL f1 ========
NUC1 1H
P1 9.60 usec
PL1 –6.00 dB
PL1W 15.19999981 W
SFO1 500.3030896 MHz

F2 – Processing parameters
SI 32768
SF 500.3000000 MHz
WDW EM
SSB 0
LB 0.30 Hz
GB 0
PC 1.00

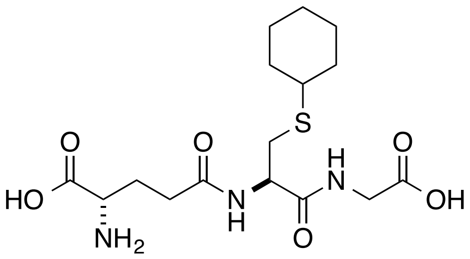

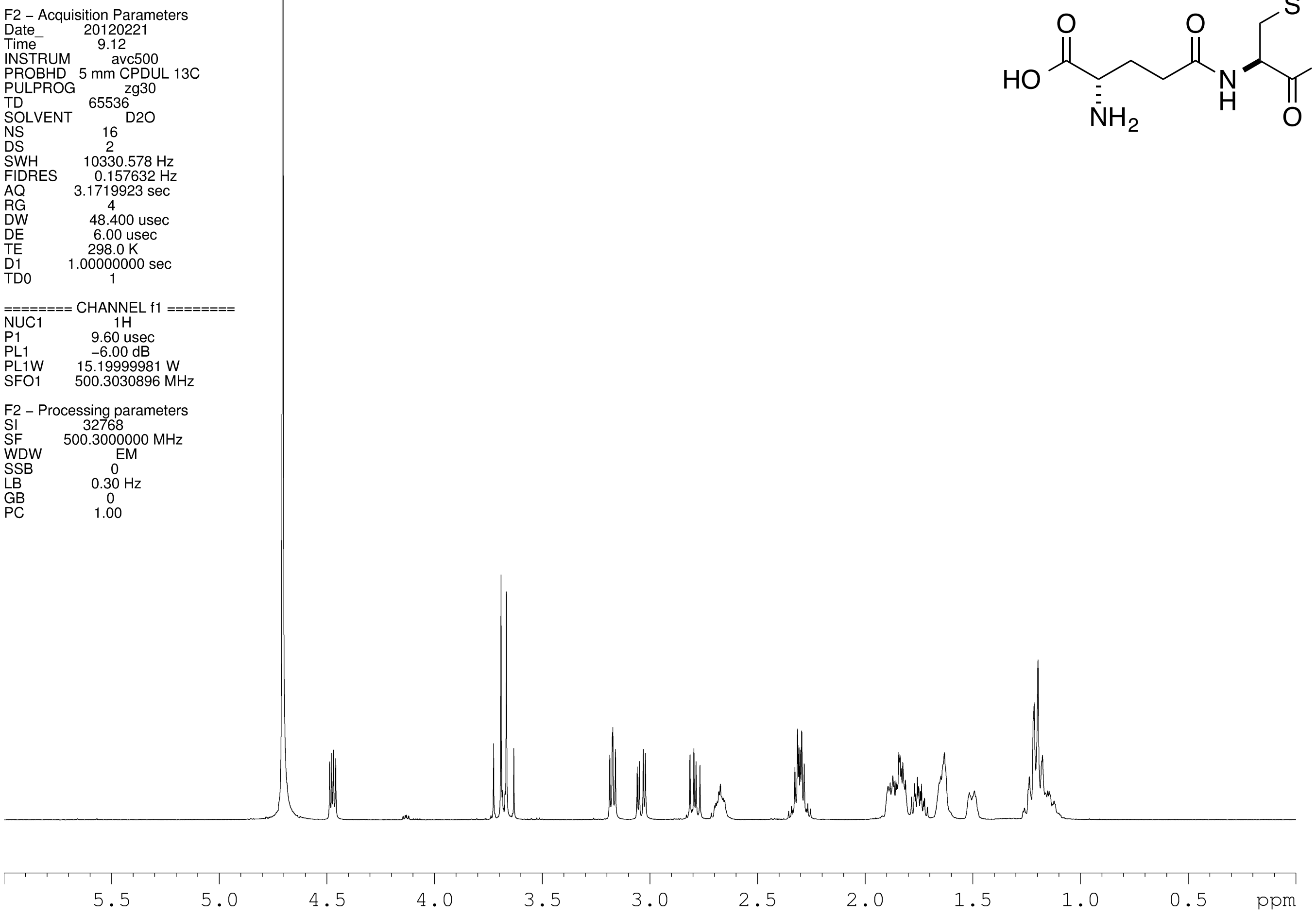

S-Cyclohexylglutathione (**5b**) – ^{13}C NMR

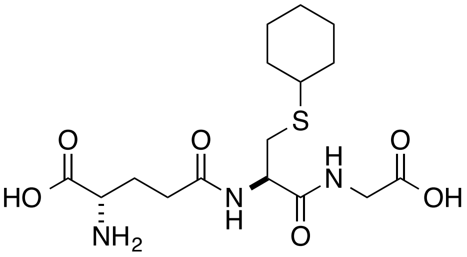

Current Data Parameters
NAME jh81302102
EXPNO 4
PROCNO 1

F2 – Acquisition Parameters
Date_ 20120221
Time 9.41
INSTRUM avc500
PROBHD 5 mm CPDUL 13C
PULPROG zgpg30
TD 65536
SOLVENT D2O
NS 1731
DS 2
SWH 31250.000 Hz
FIDRES 0.476837 Hz
AQ 1.0486259 sec
RG 1820
DW 16.000 usec
DE 20.00 usec
TE 298.0 K
D1 2.00000000 sec
D11 0.03000000 sec
TD0 1

======== CHANNEL f1 ========
NUC1 13C
P1 10.00 usec
PL1 −4.40 dB
PL1W 28.15752029 W
SFO1 125.8131151 MHz

======== CHANNEL f2 ========
CPDPRG2 waltz16
NUC2 1H
PCPD2 80.00 usec
PL2 −6.00 dB
PL12 12.42 dB
PL13 18.42 dB
PL2W 15.19999981 W
PL12W 0.21869738 W
PL13W 0.05493430 W
SFO2 500.3020012 MHz

F2 – Processing parameters
SI 32768
SF 125.8005350 MHz
WDW EM
SSB 0
LB 1.00 Hz
GB 0
PC 1.40

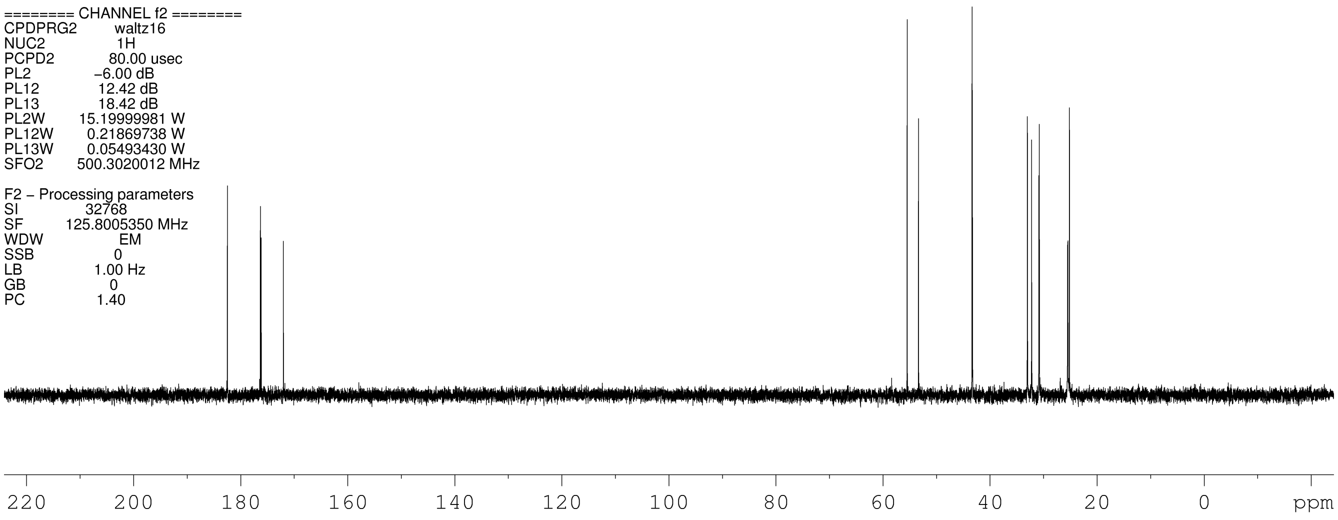

S-Decylglutathione (**5c**) – ^{1}H NMR

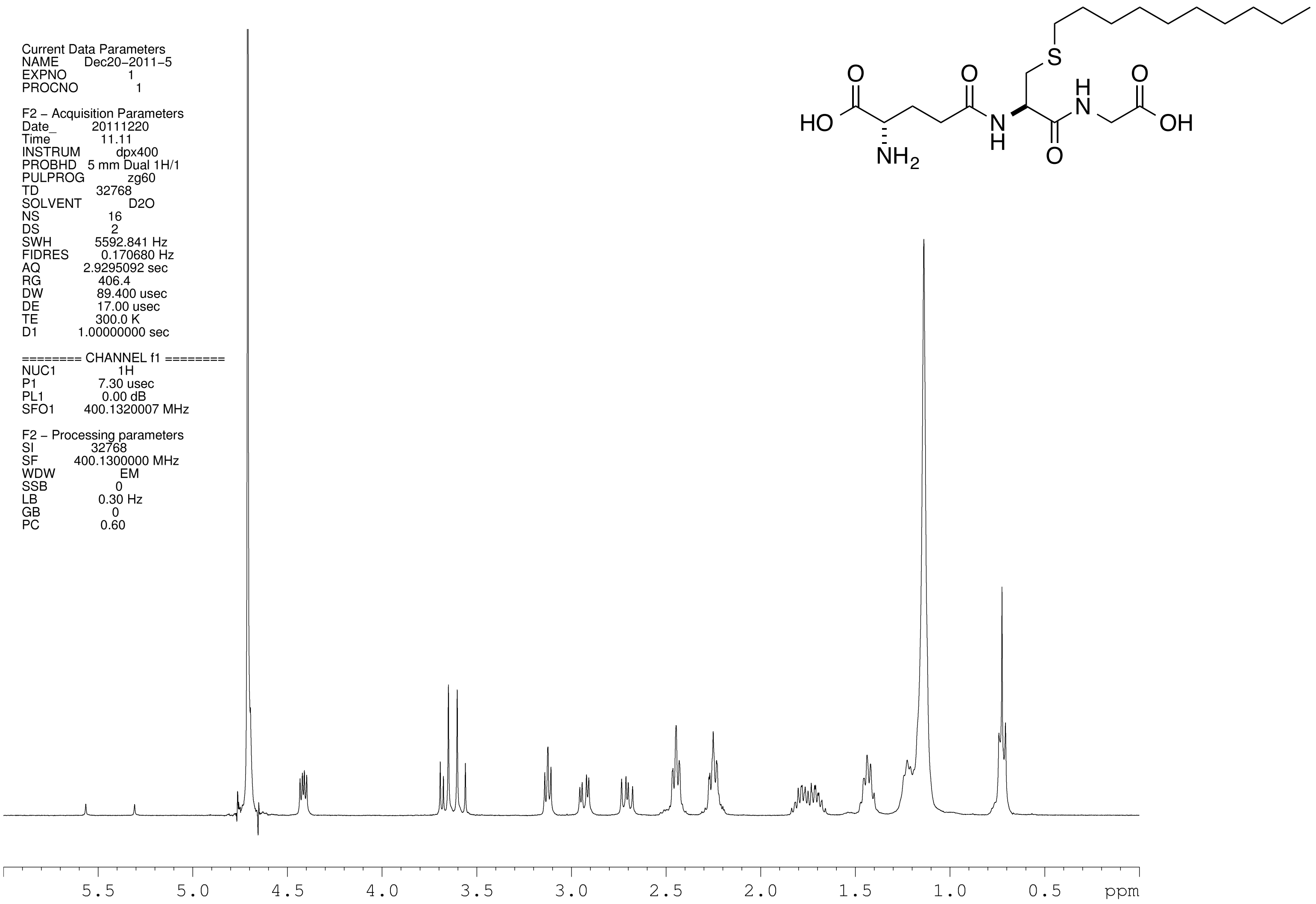

S-Dodecylglutathione (**5d**) – ^{1}H NMR

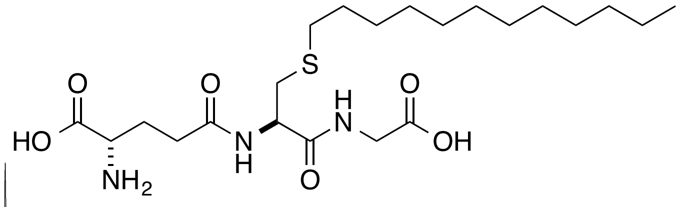

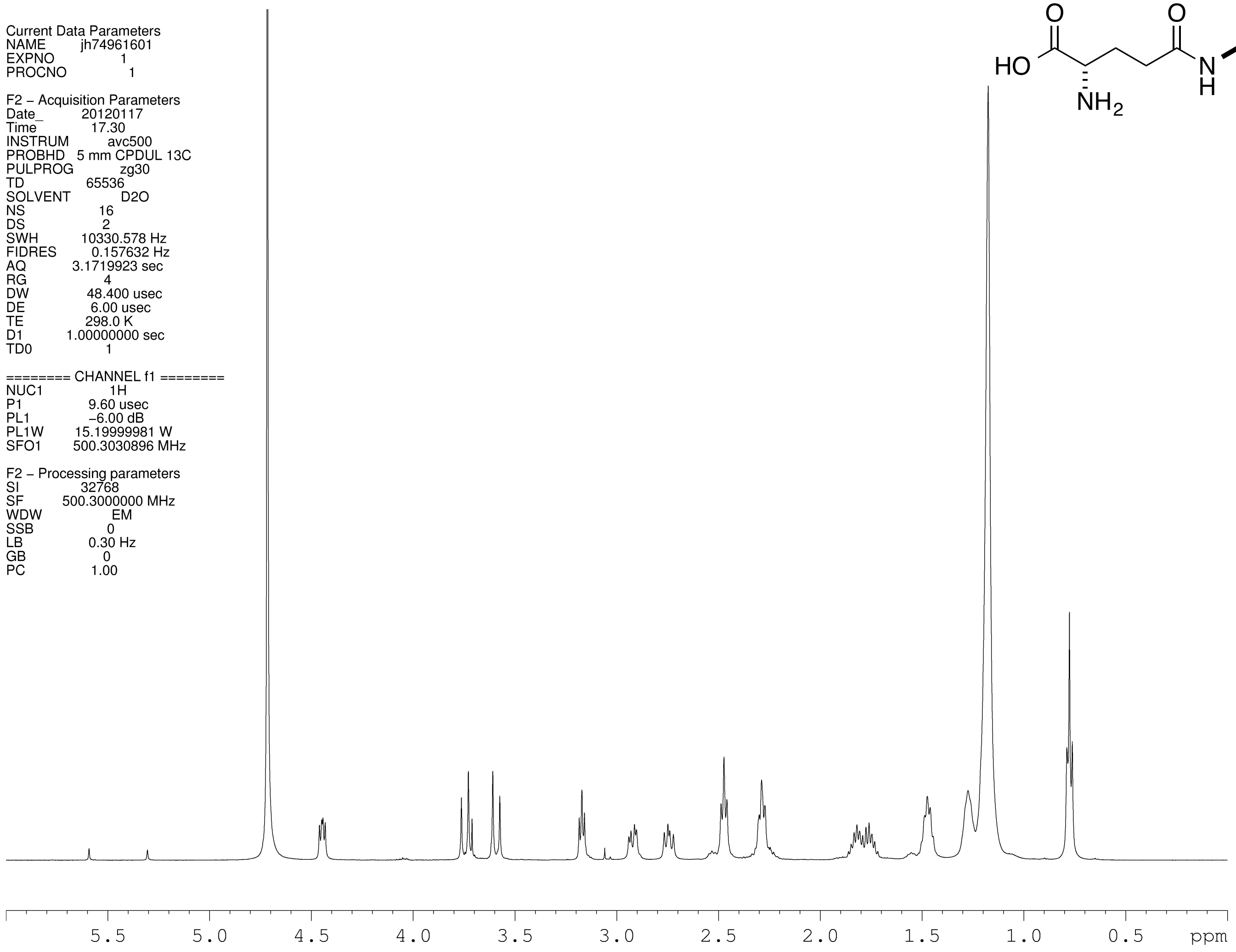

S-Dodecylglutathione (**5d**) – ^{13}C NMR

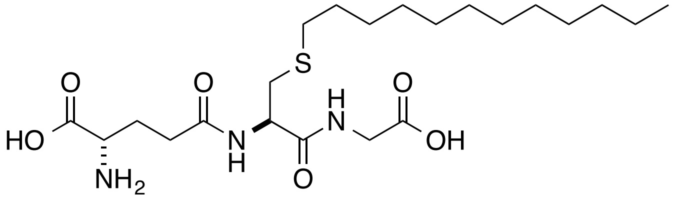

Current Data Parameters
NAME jh81302102
EXPNO 4
PROCNO 1

F2 – Acquisition Parameters
Date_ 20120221
Time 9.41
INSTRUM avc500
PROBHD 5 mm CPDUL 13C
PULPROG zgpg30
TD 65536
SOLVENT D2O
NS 1731
DS 2
SWH 31250.000 Hz
FIDRES 0.476837 Hz
AQ 1.0486259 sec
RG 1820
DW 16.000 usec
DE 20.00 usec
TE 298.0 K
D1 2.00000000 sec
D11 0.03000000 sec
TD0 1

======== CHANNEL f1 ========
NUC1 13C
P1 10.00 usec
PL1 –4.40 dB
PL1W 28.15752029 W
SFO1 125.8131151 MHz

======== CHANNEL f2 ========
CPDPRG2 waltz16
NUC2 1H
PCPD2 80.00 usec
PL2 –6.00 dB
PL12 12.42 dB
PL13 18.42 dB
PL2W 15.19999981 W
PL12W 0.21869738 W
PL13W 0.05493430 W
SFO2 500.3020012 MHz

F2 – Processing parameters
SI 32768
SF 125.8005350 MHz
WDW EM
SSB 0
LB 1.00 Hz
GB 0
PC 1.40

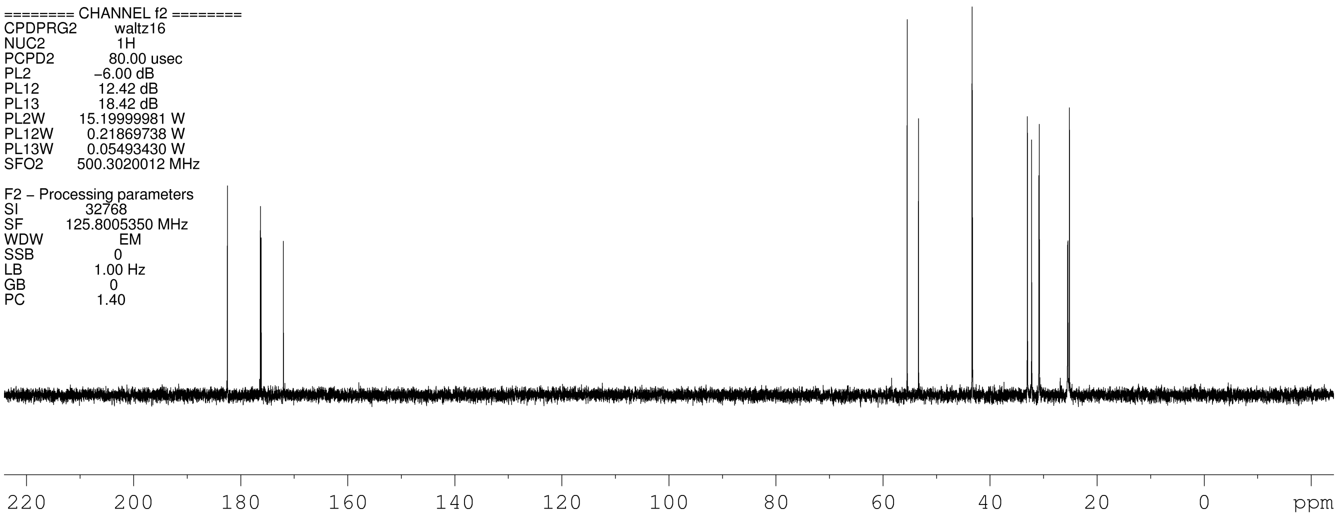

S-Hexadecylglutathione (**5e**) – ^{1}H NMR

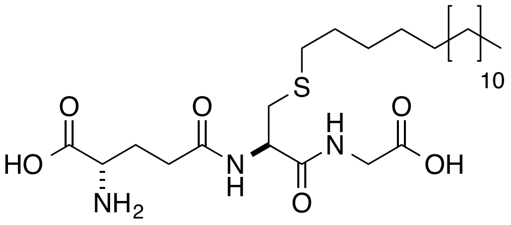

Current Data Parameters
NAME jh74951601
EXPNO 1
PROCNO 1

F2 – Acquisition Parameters
Date_ 20120117
Time 14.57
INSTRUM avc500
PROBHD 5 mm CPDUL 13C
PULPROG zg30
TD 65536
SOLVENT D2O
NS 16
DS 2
SWH 10330.578 Hz
FIDRES 0.157632 Hz
AQ 3.1719923 sec
RG 4
DW 48.400 usec
DE 6.00 usec
TE 298.0 K
D1 1.00000000 sec
TD0 1

======== CHANNEL f1 ========
NUC1 1H
P1 9.60 usec
PL1 –6.00 dB
PL1W 15.19999981 W
SFO1 500.3030896 MHz

F2 – Processing parameters
SI 32768
SF 500.3000000 MHz
WDW EM
SSB 0
LB 0.30 Hz
GB 0
PC 1.00

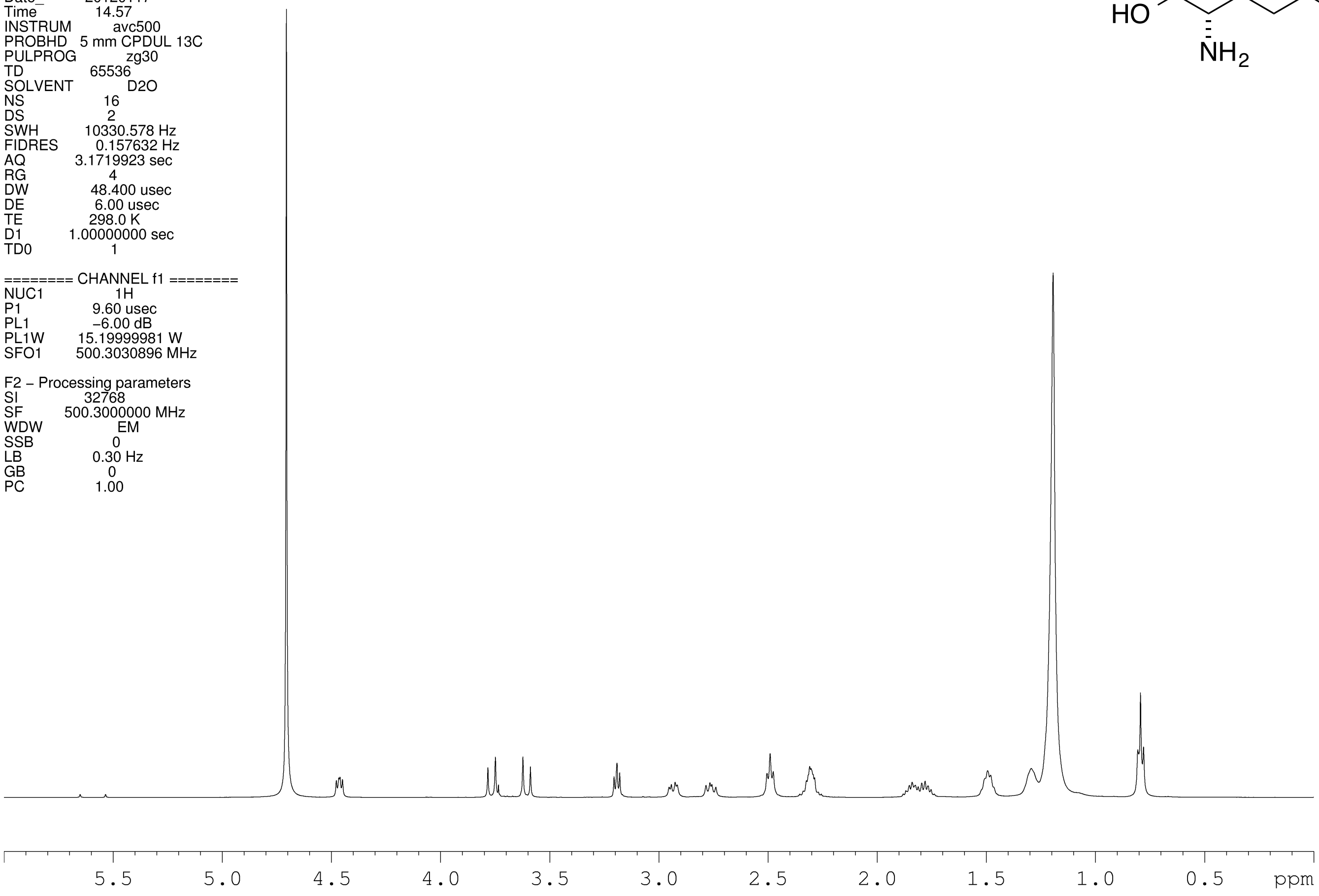

S-Hexadecylglutathione (**5e**) – ^{13}C NMR

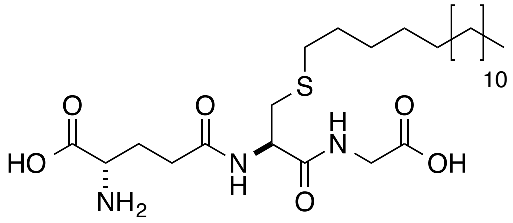

Current Data Parameters
NAME jh81302102
EXPNO 4
PROCNO 1

F2 - Acquisition Parameters
Date_ 20120221
Time 9.41
INSTRUM avc500
PROBHD 5 mm CPDUL 13C
PULPROG zgpg30
TD 65536
SOLVENT D2O
NS 1731
DS 2
SWH 31250.000 Hz
FIDRES 0.476837 Hz
AQ 1.0486259 sec
RG 1820
DW 16.000 usec
DE 20.00 usec
TE 298.0 K
D1 2.00000000 sec
D11 0.03000000 sec
TD0 1

======== CHANNEL f1 ========
NUC1 13C
P1 10.00 usec
PL1 -4.40 dB
PL1W 28.15752029 W
SFO1 125.8131151 MHz

======== CHANNEL f2 ========
CPDPRG2 waltz16
NUC2 1H
PCPD2 80.00 usec
PL2 -6.00 dB
PL12 12.42 dB
PL13 18.42 dB
PL2W 15.19999981 W
PL12W 0.21869738 W
PL13W 0.05493430 W
SFO2 500.3020012 MHz

F2 - Processing parameters
SI 32768
SF 125.8005350 MHz
WDW EM
SSB 0
LB 1.00 Hz
GB 0
PC 1.40

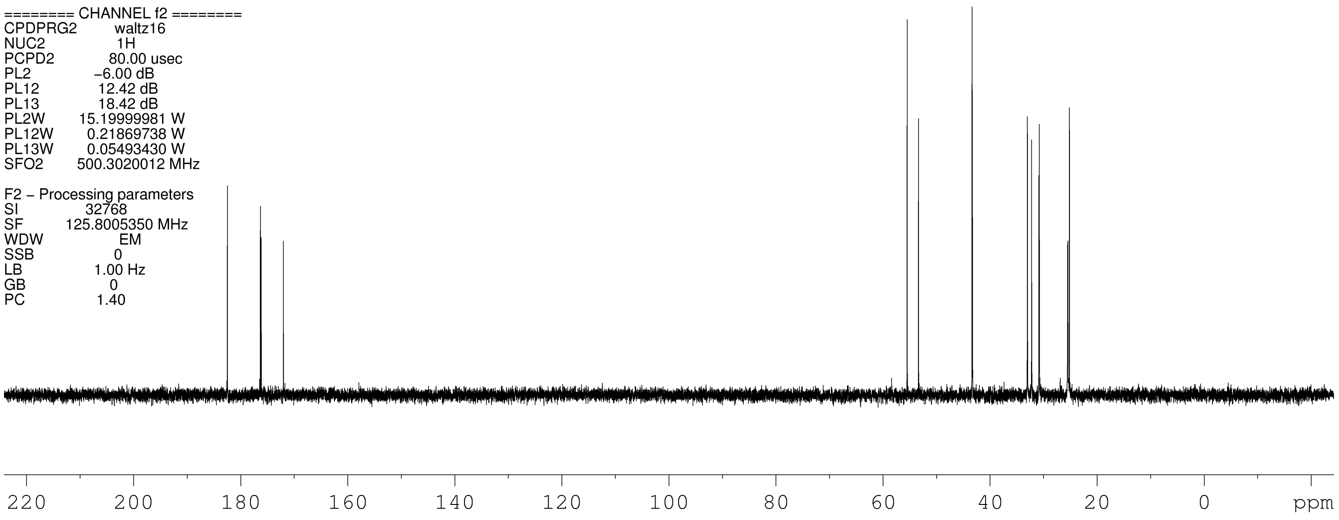

S-Propyltrimethylsilylglutathione (**5f**) – ^{1}H NMR

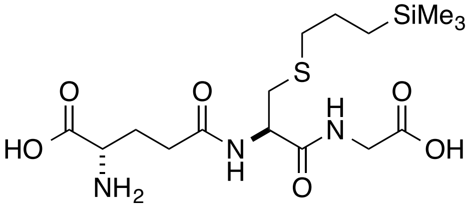

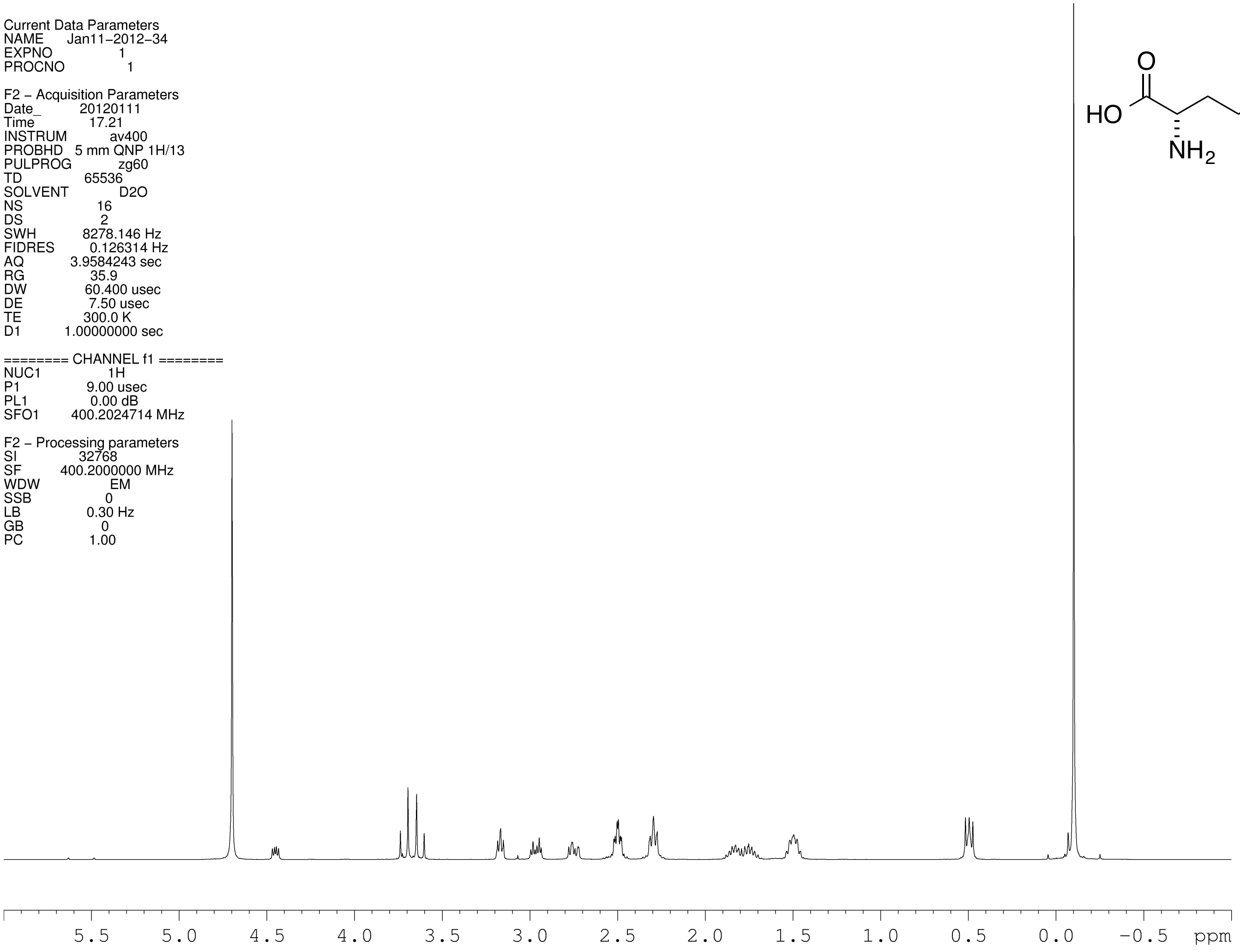

S-Propyltrimethylsilylglutathione (**5f**) – ^{13}C NMR

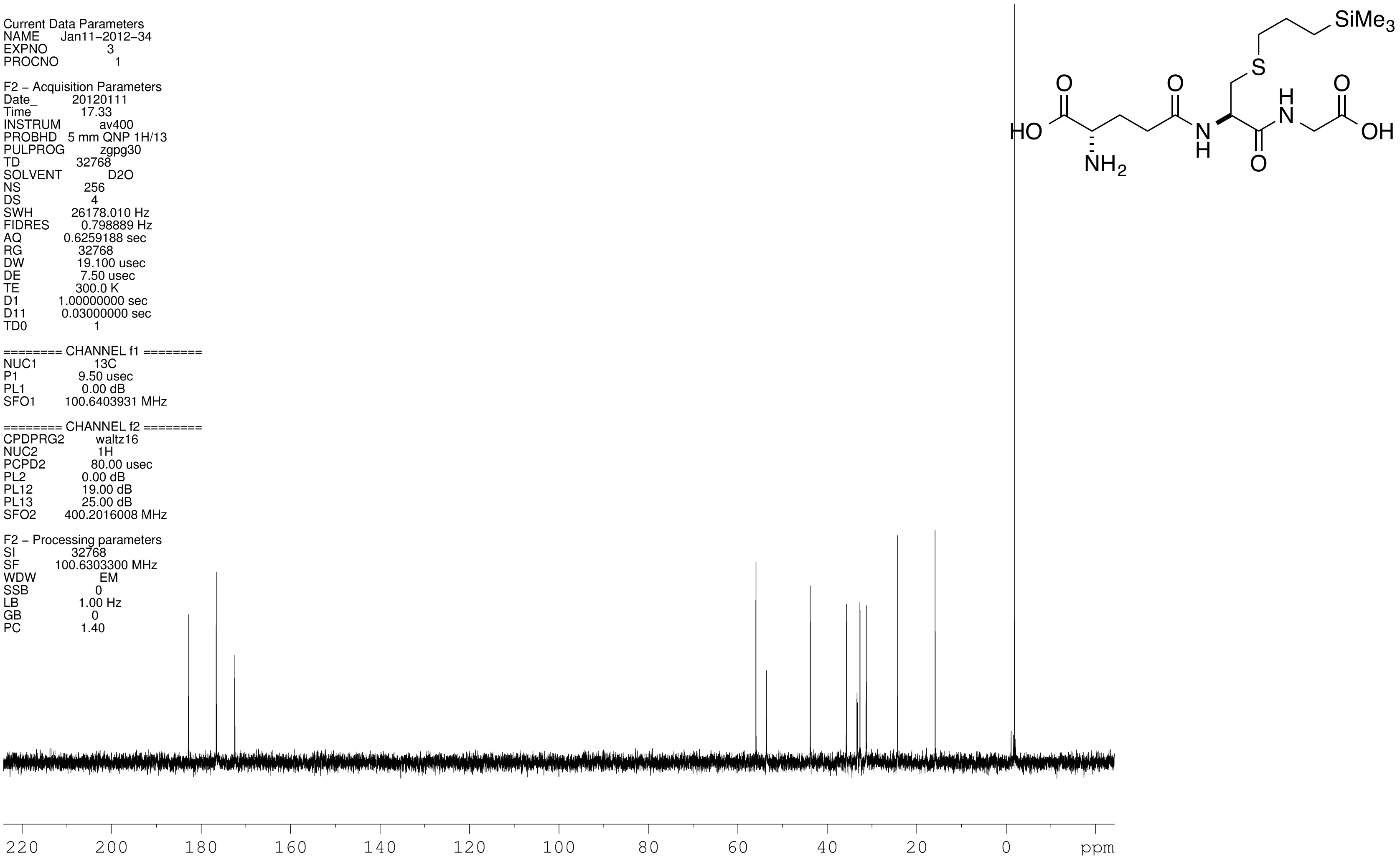

S-3,3-Dimethylbutylglutathione (**5g**) – ^{1}H NMR

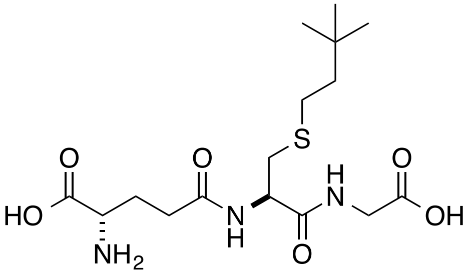

Current Data Parameters
NAME jh74351201
EXPNO 1
PROCNO 1

F2 – Acquisition Parameters
Date_ 20120112
Time 8.56
INSTRUM avc500
PROBHD 5 mm CPDUL 13C
PULPROG zg30
TD 65536
SOLVENT D2O
NS 16
DS 2
SWH 10330.578 Hz
FIDRES 0.157632 Hz
AQ 3.1719923 sec
RG 4
DW 48.400 usec
DE 6.00 usec
TE 298.0 K
D1 1.00000000 sec
TD0 1

======== CHANNEL f1 ========
NUC1 1H
P1 9.60 usec
PL1 –6.00 dB
PL1W 15.19999981 W
SFO1 500.3030896 MHz

F2 – Processing parameters
SI 32768
SF 500.3000000 MHz
WDW EM
SSB 0
LB 0.30 Hz
GB 0
PC 1.00

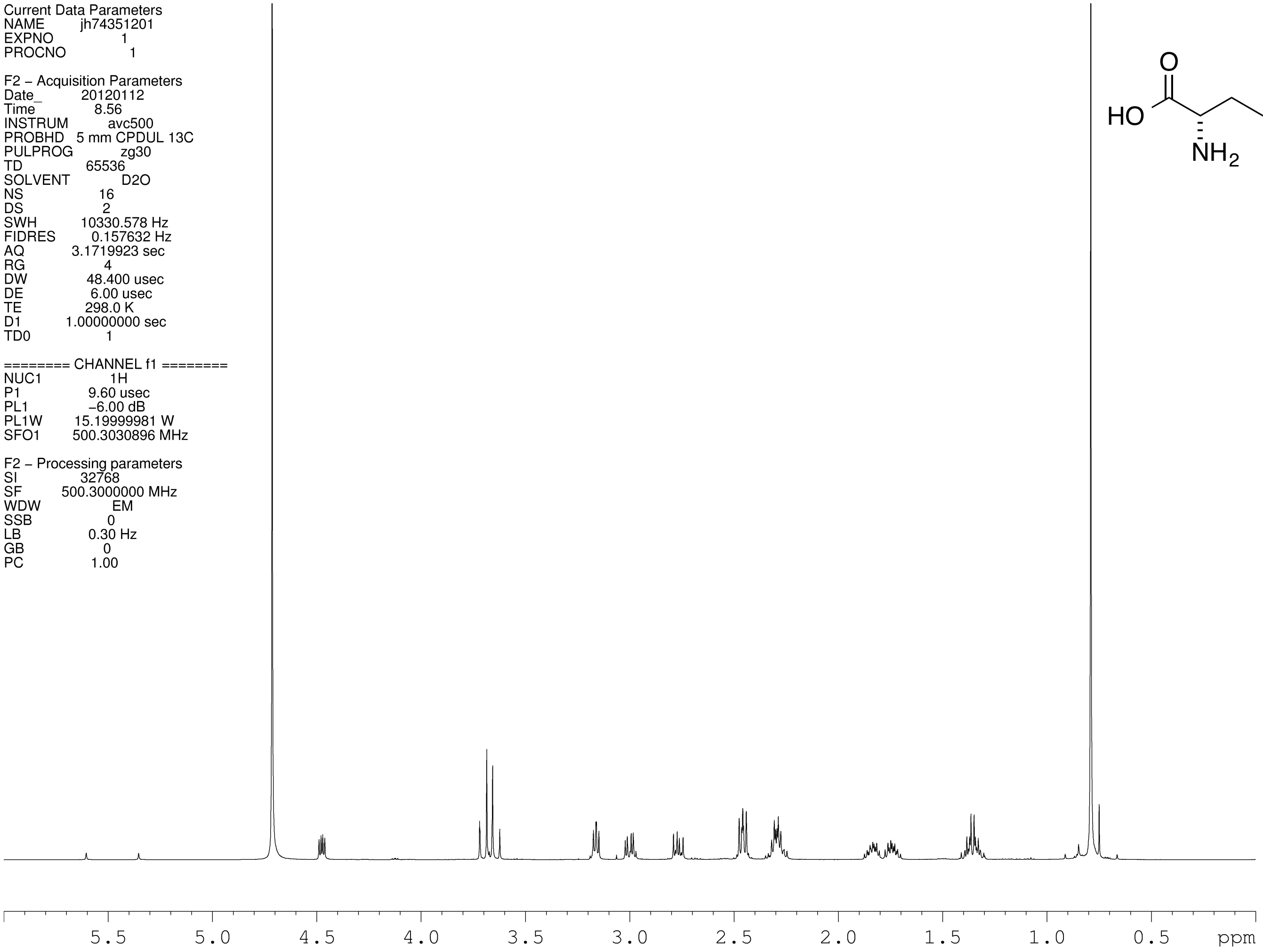

S-3,3-Dimethylbutylglutathione (**5g**) – ^{13}C NMR

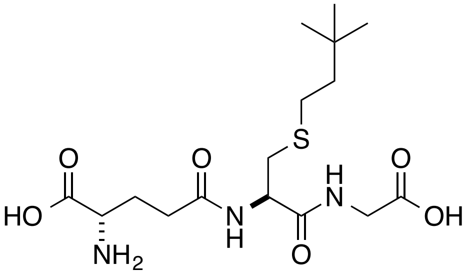

Current Data Parameters
NAME jh74351201
EXPNO 4
PROCNO 1

F2 – Acquisition Parameters
Date_ 20120112
Time 12.10
INSTRUM avc500
PROBHD 5 mm CPDUL 13C
PULPROG zgpg30
TD 65536
SOLVENT D2O
NS 1024
DS 2
SWH 31250.000 Hz
FIDRES 0.476837 Hz
AQ 1.0486259 sec
RG 812
DW 16.000 usec
DE 20.00 usec
TE 298.0 K
D1 2.00000000 sec
D11 0.03000000 sec
TD0 1

======== CHANNEL f1 ========
NUC1 13C
P1 10.00 usec
PL1 –4.40 dB
PL1W 28.15752029 W
SFO1 125.8131151 MHz

======== CHANNEL f2 ========
CPDPRG2 waltz16
NUC2 1H
PCPD2 80.00 usec
PL2 –6.00 dB
PL12 12.42 dB
PL13 18.42 dB
PL2W 15.19999981 W
PL12W 0.21869738 W
PL13W 0.05493430 W
SFO2 500.3020012 MHz

F2 – Processing parameters
SI 32768
SF 125.8005350 MHz
WDW EM
SSB 0
LB 1.00 Hz
GB 0
PC 1.40

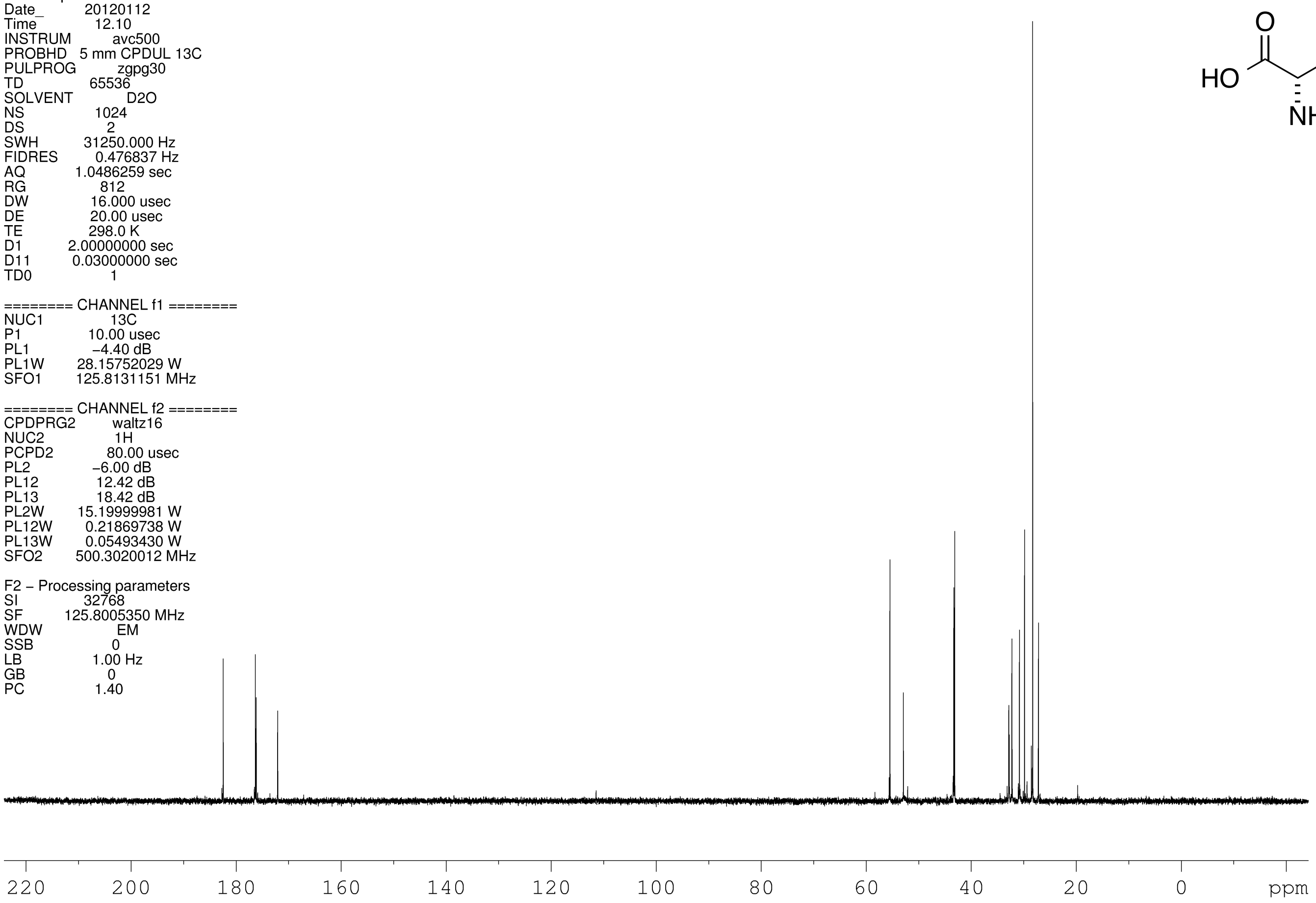

S-Propanol glutathione (**5h**) – ^{1}H HMR

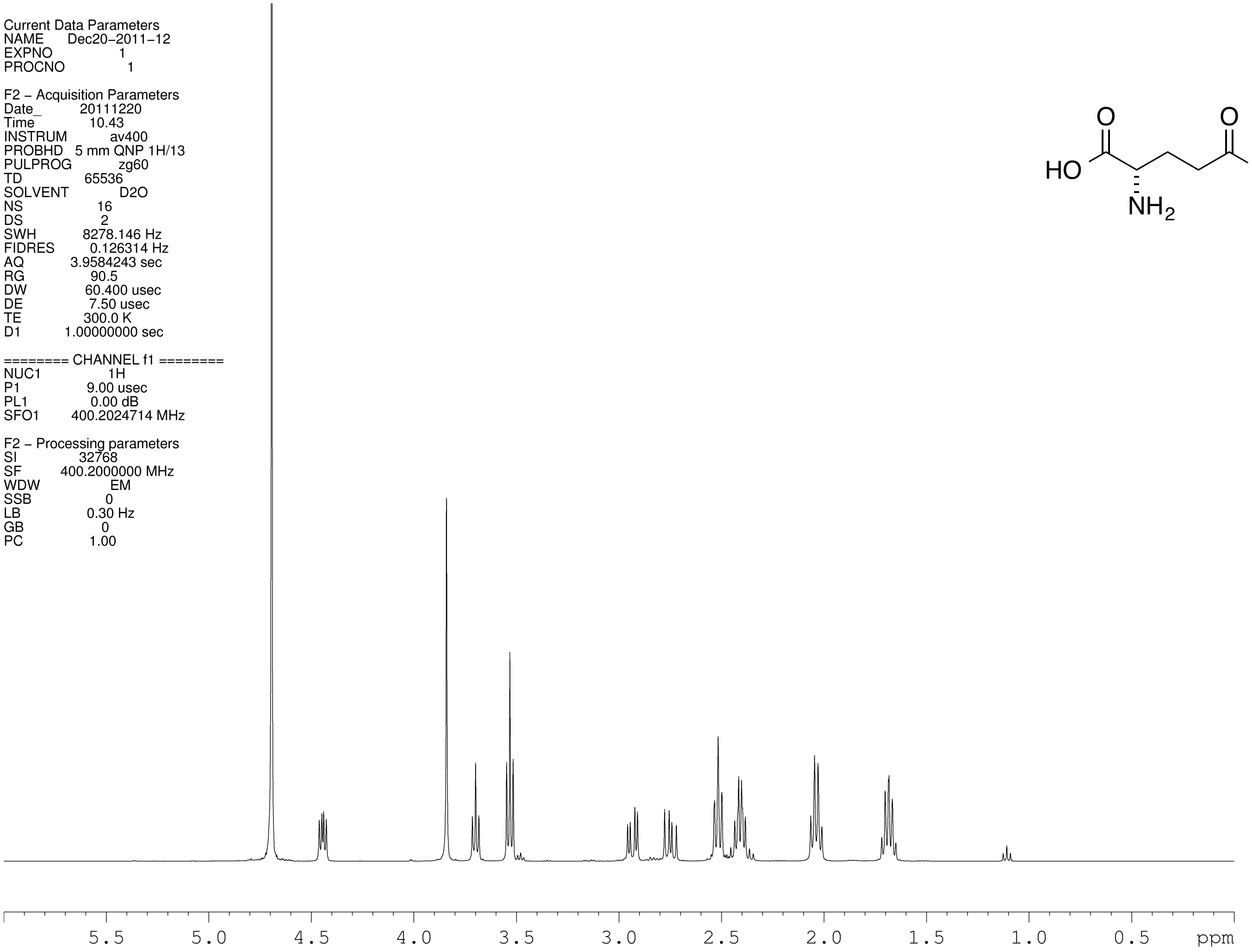

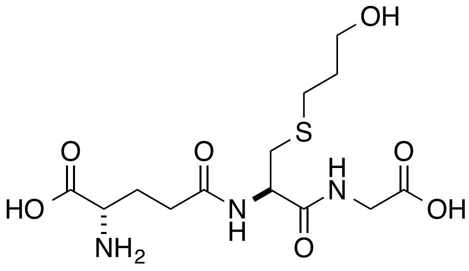

S-Propanol glutathione (**5h**) – ^{13}C HMR

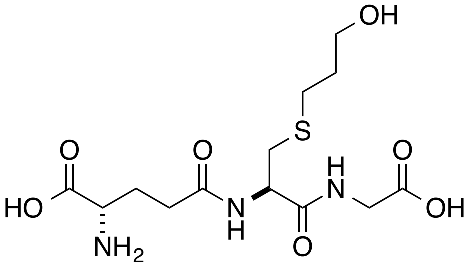

Current Data Parameters
NAME Dec20−2011−12
EXPNO 3
PROCNO 1

F2 − Acquisition Parameters
Date_ 20111220
Time 10.55
INSTRUM av400
PROBHD 5 mm QNP 1H/13
PULPROG zgpg30
TD 32768
SOLVENT D2O
NS 256
DS 4
SWH 26178.010 Hz
FIDRES 0.798889 Hz
AQ 0.6259188 sec
RG 32768
DW 19.100 usec
DE 7.50 usec
TE 300.0 K
D1 1.00000000 sec
D11 0.03000000 sec
TD0 1

======== CHANNEL f1 ========
NUC1 13C
P1 9.50 usec
PL1 0.00 dB
SFO1 100.6403931 MHz

======== CHANNEL f2 ========
CPDPRG2 waltz16
NUC2 1H
PCPD2 80.00 usec
PL2 0.00 dB
PL12 19.00 dB
PL13 25.00 dB
SFO2 400.2016008 MHz

F2 − Processing parameters
SI 32768
SF 100.6303300 MHz
WDW EM
SSB 0
LB 1.00 Hz
GB 0
PC 1.40

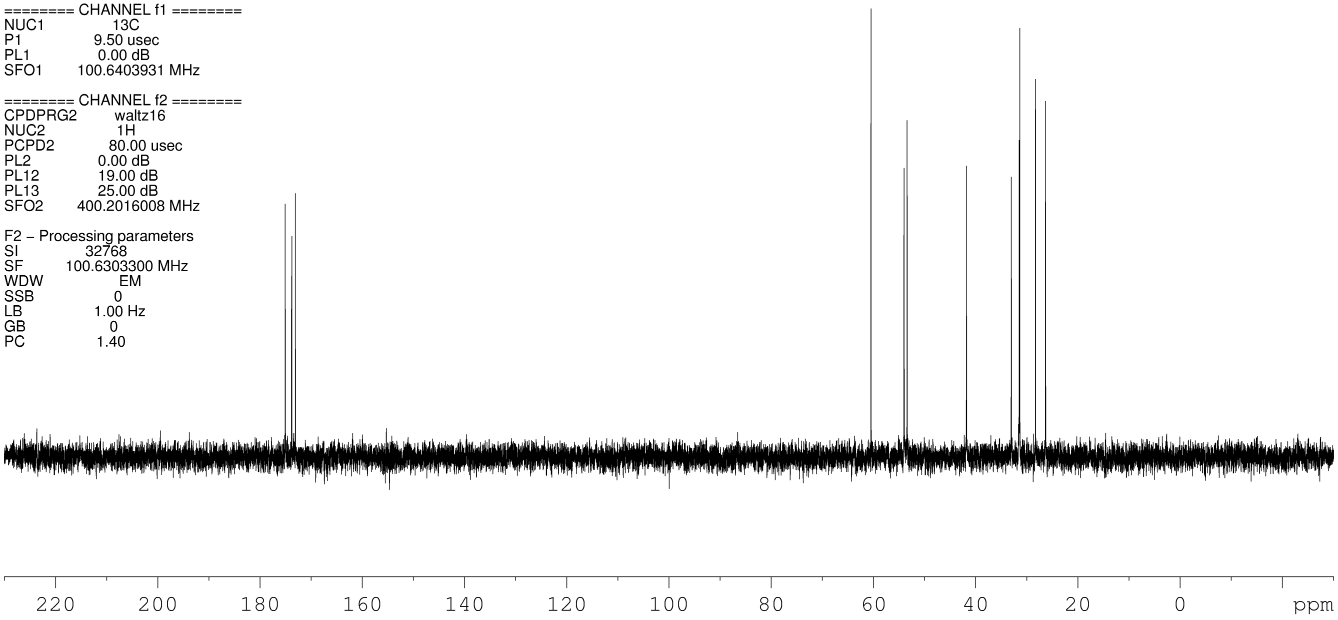

S-Propylamino glutathione (**5i**) – ^{1}H NMR

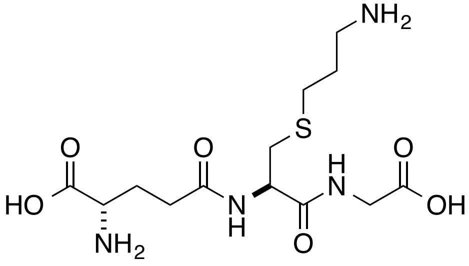

Current Data Parameters
NAME jh80411502
EXPNO 1
PROCNO 1

F2 – Acquisition Parameters
Date_ 20120216
Time 10.31
INSTRUM avc500
PROBHD 5 mm CPDUL 13C
PULPROG zg30
TD 65536
SOLVENT D2O
NS 16
DS 2
SWH 10330.578 Hz
FIDRES 0.157632 Hz
AQ 3.1719923 sec
RG 4
DW 48.400 usec
DE 6.00 usec
TE 298.0 K
D1 1.00000000 sec
TD0 1

======== CHANNEL f1 ========
NUC1 1H
P1 9.60 usec
PL1 –6.00 dB
PL1W 15.19999981 W
SFO1 500.3030896 MHz

F2 – Processing parameters
SI 32768
SF 500.3000000 MHz
WDW EM
SSB 0
LB 0.30 Hz
GB 0
PC 1.00

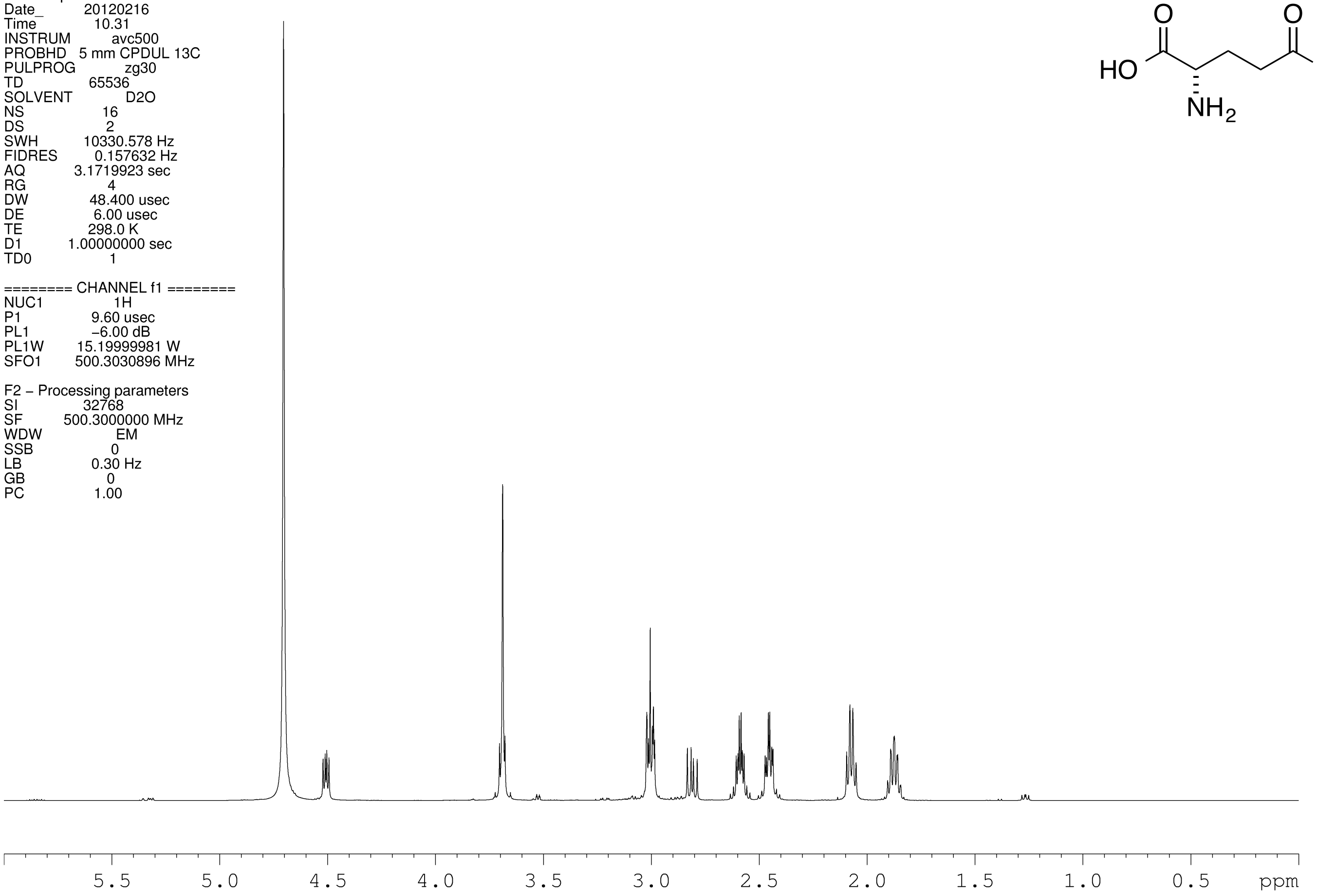

S-Propylamino glutathione (**5i**) – ^{13}C NMR

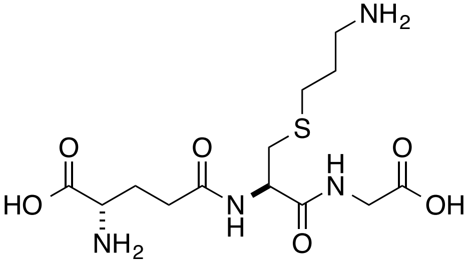

Current Data Parameters
NAME jh80411502
EXPNO 4
PROCNO 1

F2 - Acquisition Parameters
Date_ 20120216
Time 11.46
INSTRUM avc500
PROBHD 5 mm CPDUL 13C
PULPROG zgpg30
TD 65536
SOLVENT D2O
NS 1024
DS 2
SWH 31250.000 Hz
FIDRES 0.476837 Hz
AQ 1.0486259 sec
RG 1440
DW 16.000 usec
DE 20.00 usec
TE 298.0 K
D1 2.00000000 sec
D11 0.03000000 sec
TD0 1

======== CHANNEL f1 ========
NUC1 13C
P1 10.00 usec
PL1 -4.40 dB
PL1W 28.15752029 W
SFO1 125.8131151 MHz

======== CHANNEL f2 ========
CPDPRG2 waltz16
NUC2 1H
PCPD2 80.00 usec
PL2 -6.00 dB
PL12 12.42 dB
PL13 18.42 dB
PL2W 15.19999981 W
PL12W 0.21869738 W
PL13W 0.05493430 W
SFO2 500.3020012 MHz

F2 - Processing parameters
SI 32768
SF 125.8005350 MHz
WDW EM
SSB 0
LB 1.00 Hz
GB 0
PC 1.40

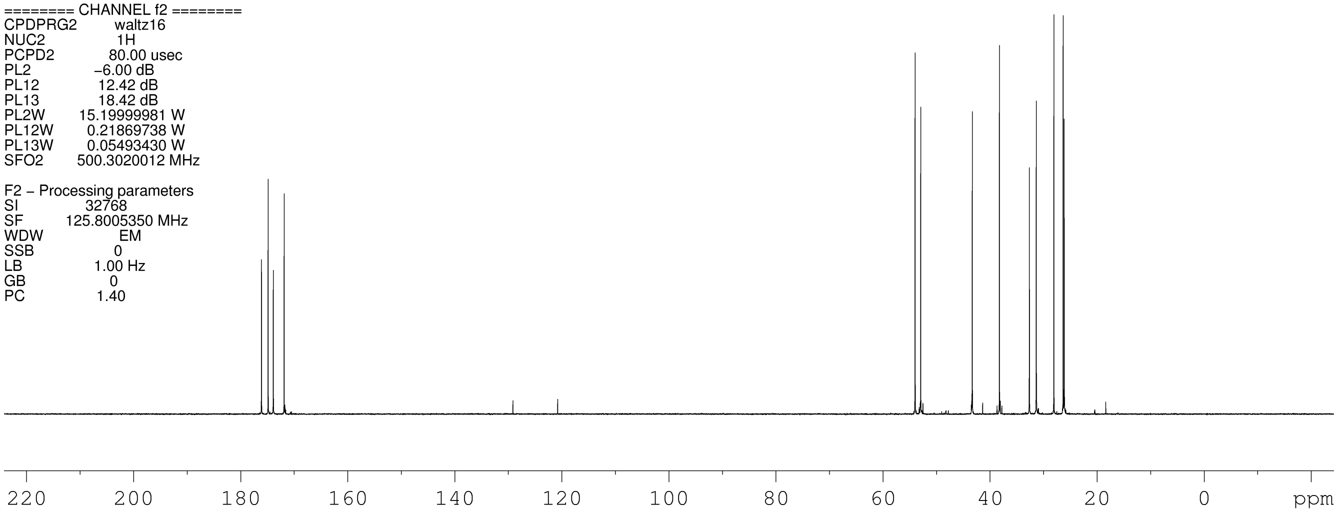

S-1-Ethyl-*N*-pyrrolidinone glutathione (**5j**) – ^{1}H NMR

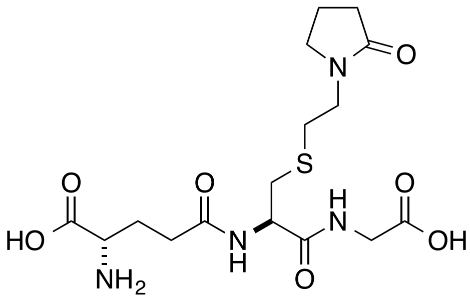

Current Data Parameters
NAME Jan23–2012–15
EXPNO 1
PROCNO 1

F2 – Acquisition Parameters
Date_ 20120123
Time 14.14
INSTRUM av400
PROBHD 5 mm QNP 1H/13
PULPROG zg60
TD 65536
SOLVENT D2O
NS 16
DS 2
SWH 8278.146 Hz
FIDRES 0.126314 Hz
AQ 3.9584243 sec
RG 35.9
DW 60.400 usec
DE 7.50 usec
TE 300.0 K
D1 1.00000000 sec

======== CHANNEL f1 ========
NUC1 1H
P1 9.00 usec
PL1 0.00 dB
SFO1 400.2024714 MHz

F2 – Processing parameters
SI 32768
SF 400.2000000 MHz
WDW EM
SSB 0
LB 0.30 Hz
GB 0
PC 1.00

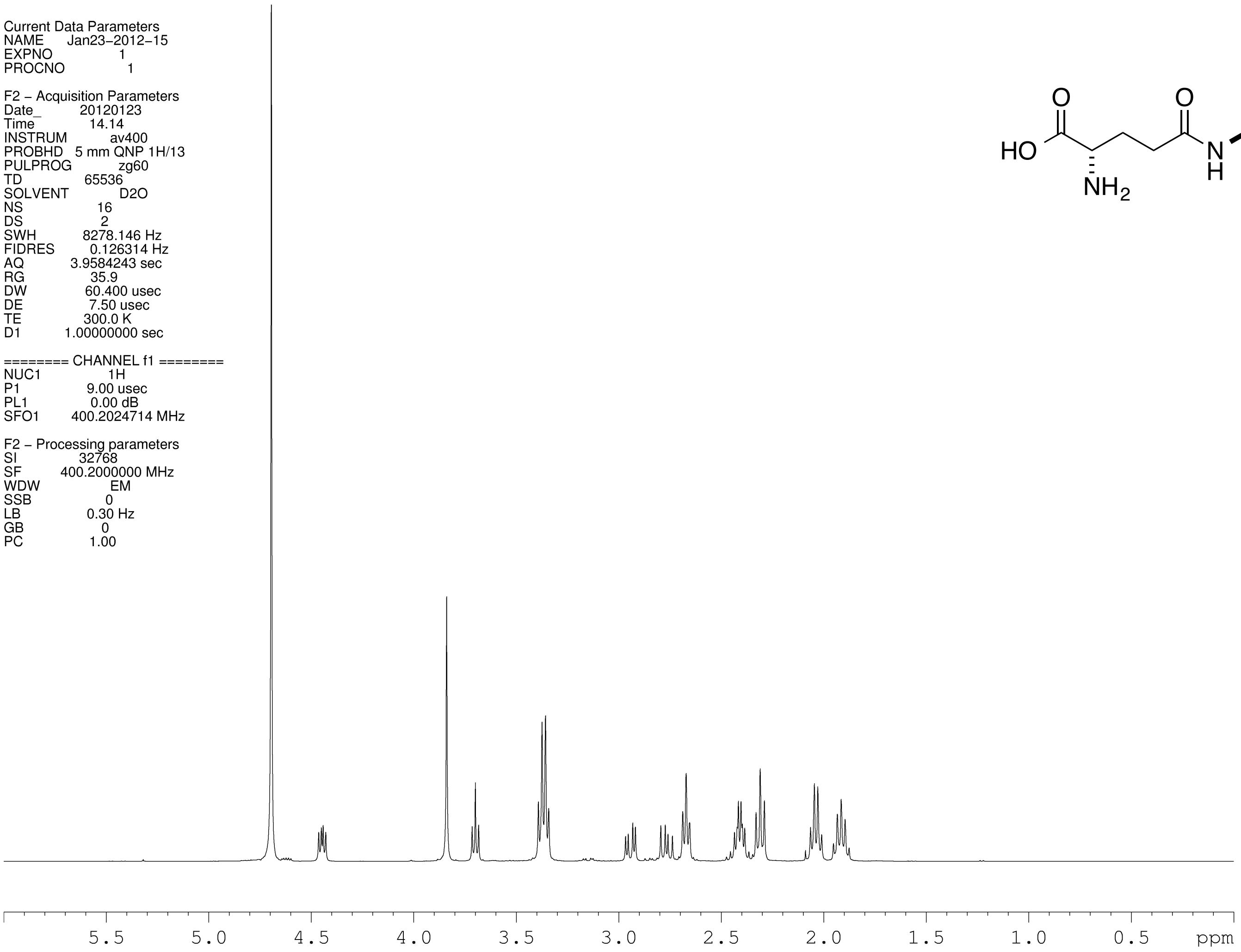

S-1-Ethyl-*N*-pyrrolidinone glutathione (**5j**) – ^{13}CMR

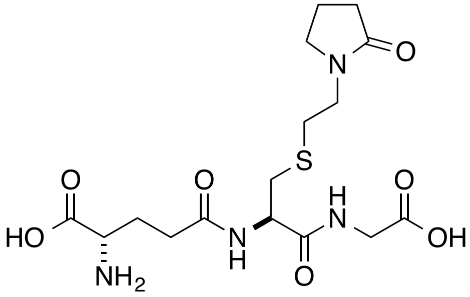

Current Data Parameters
NAME Jan23−2012−15
EXPNO 3
PROCNO 1

F2 − Acquisition Parameters
Date_ 20120123
Time 14.26
INSTRUM av400
PROBHD 5 mm QNP 1H/13
PULPROG zgpg30
TD 32768
SOLVENT D2O
NS 256
DS 4
SWH 26178.010 Hz
FIDRES 0.798889 Hz
AQ 0.6259188 sec
RG 32768
DW 19.100 usec
DE 7.50 usec
TE 300.0 K
D1 1.00000000 sec
D11 0.03000000 sec
TD0 1

======== CHANNEL f1 ========
NUC1 13C
P1 9.50 usec
PL1 0.00 dB
SFO1 100.6403931 MHz

======== CHANNEL f2 ========
CPDPRG2 waltz16
NUC2 1H
PCPD2 80.00 usec
PL2 0.00 dB
PL12 19.00 dB
PL13 25.00 dB
SFO2 400.2016008 MHz

F2 − Processing parameters
SI 32768
SF 100.6303300 MHz
WDW EM
SSB 0
LB 1.00 Hz
GB 0
PC 1.40

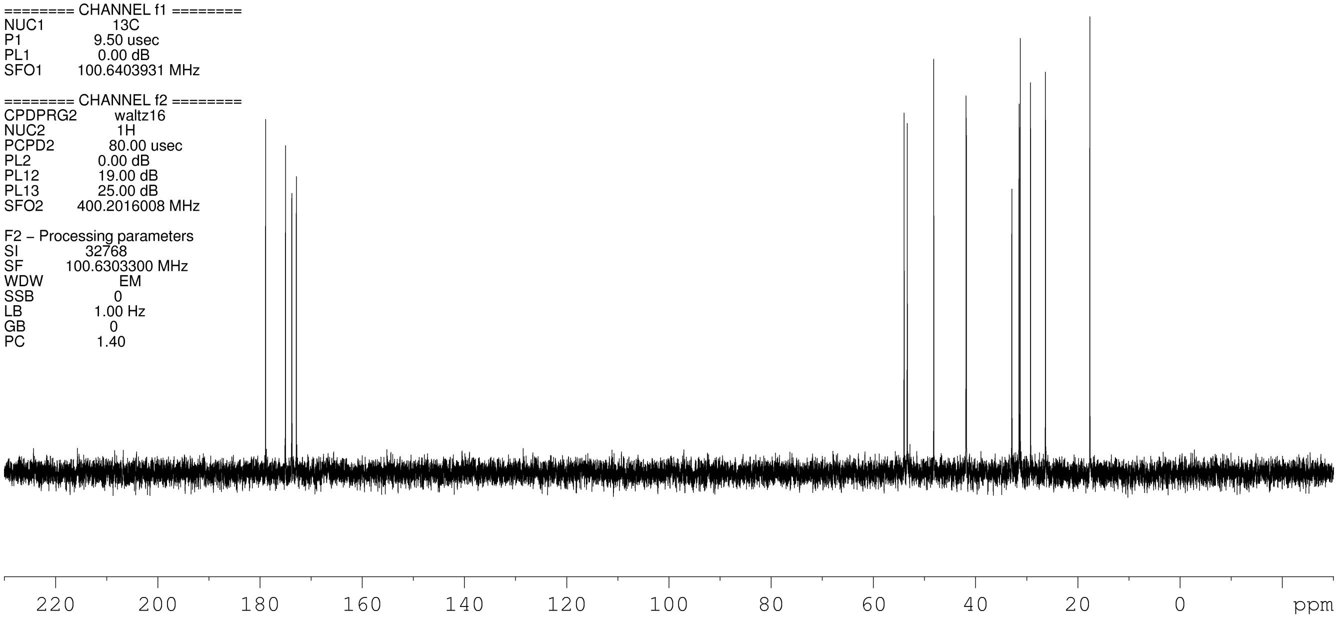

S-(6,6-dimethylbicyclo[3.1.1]heptan-2-yl)methyl glutathione (**5k**) – ^{1}H NMR

Current Data Parameters
NAME Jan30–2012–15
EXPNO 1
PROCNO 1

F2 – Acquisition Parameters
Date_ 20120130
Time 21.28
INSTRUM dpx400
PROBHD 5 mm Dual 1H/1
PULPROG zg60
TD 32768
SOLVENT D2O
NS 16
DS 2
SWH 5592.841 Hz
FIDRES 0.170680 Hz
AQ 2.9295092 sec
RG 645.1
DW 89.400 usec
DE 17.00 usec
TE 300.0 K
D1 1.00000000 sec

======== CHANNEL f1 ========
NUC1 1H
P1 7.30 usec
PL1 0.00 dB
SFO1 400.1320007 MHz

F2 – Processing parameters
SI 32768
SF 400.1300000 MHz
WDW EM
SSB 0
LB 0.30 Hz
GB 0
PC 0.60

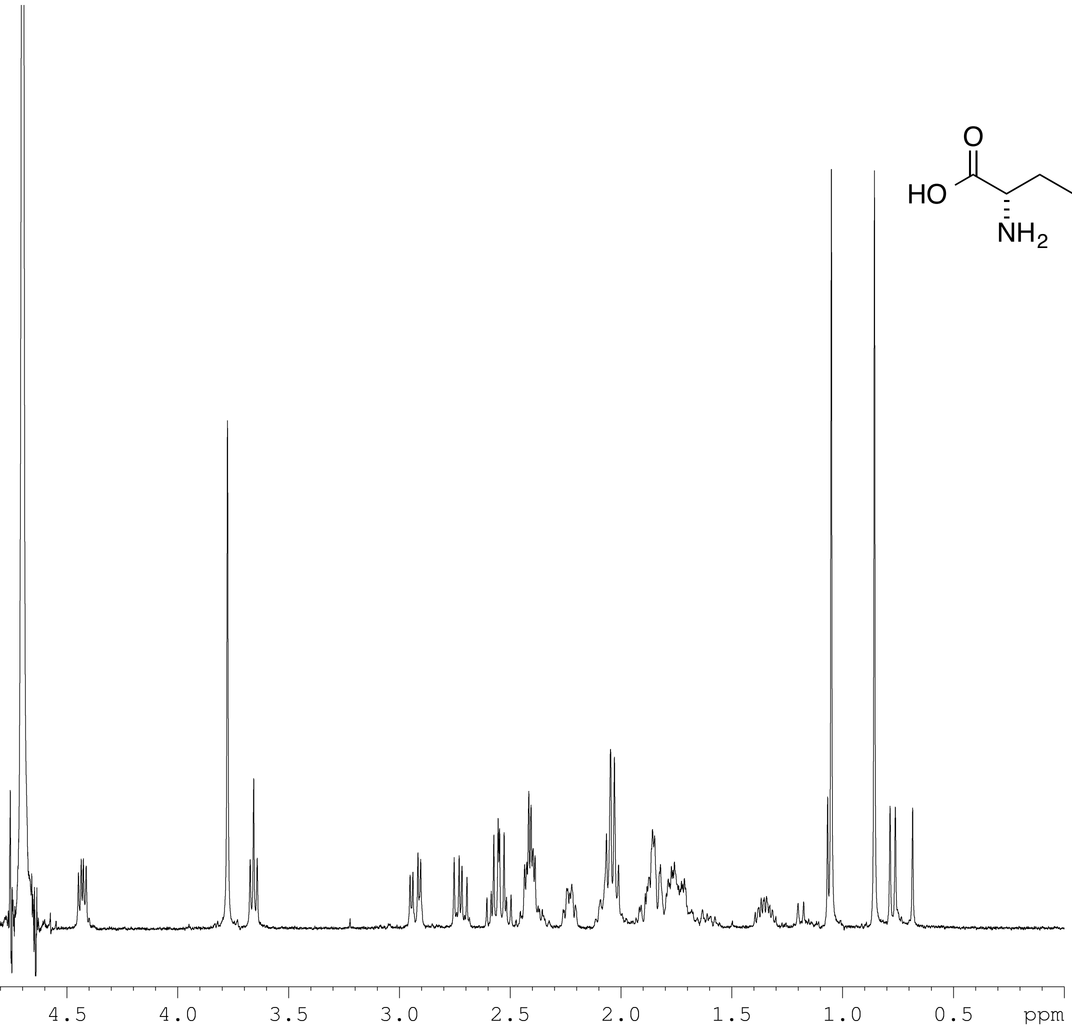

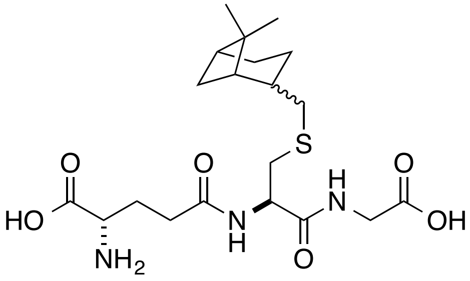

S-Ethyl-4-nitrophenyl glutathione (**6a**) – ^{1}H NMR

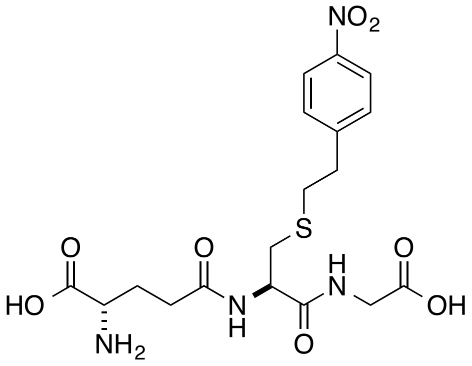

Current Data Parameters
NAME Feb16–2012–59
EXPNO 1
PROCNO 1

F2 – Acquisition Parameters
Date_ 20120216
Time 17.18
INSTRUM dpx400
PROBHD 5 mm Dual 1H/1
PULPROG zg60
TD 32768
SOLVENT D2O
NS 16
DS 2
SWH 5592.841 Hz
FIDRES 0.170680 Hz
AQ 2.9295092 sec
RG 456.1
DW 89.400 usec
DE 17.00 usec
TE 300.0 K
D1 1.00000000 sec

======== CHANNEL f1 ========
NUC1 1H
P1 7.30 usec
PL1 0.00 dB
SFO1 400.1320007 MHz

F2 – Processing parameters
SI 32768
SF 400.1300000 MHz
WDW EM
SSB 0
LB 0.30 Hz
GB 0
PC 0.60

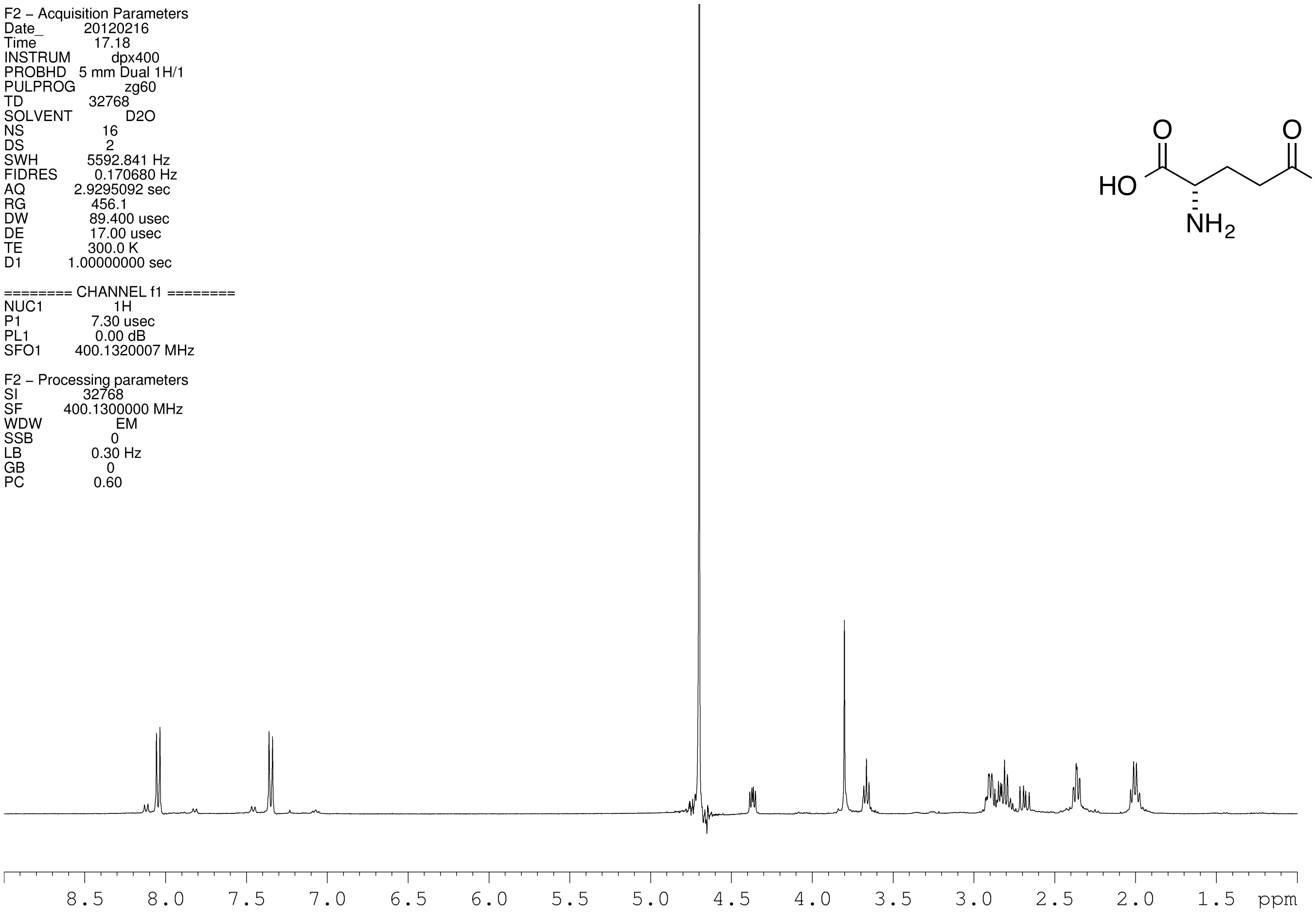

S-Ethyl-2-trifluoromethylphenyl glutathione (**6b**) – ^{1}H NMR

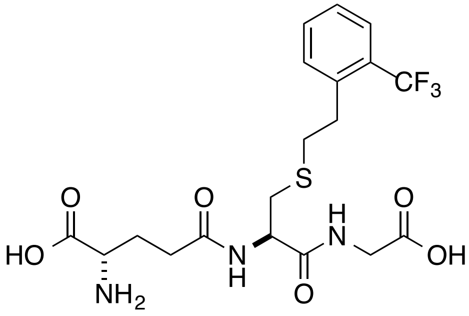

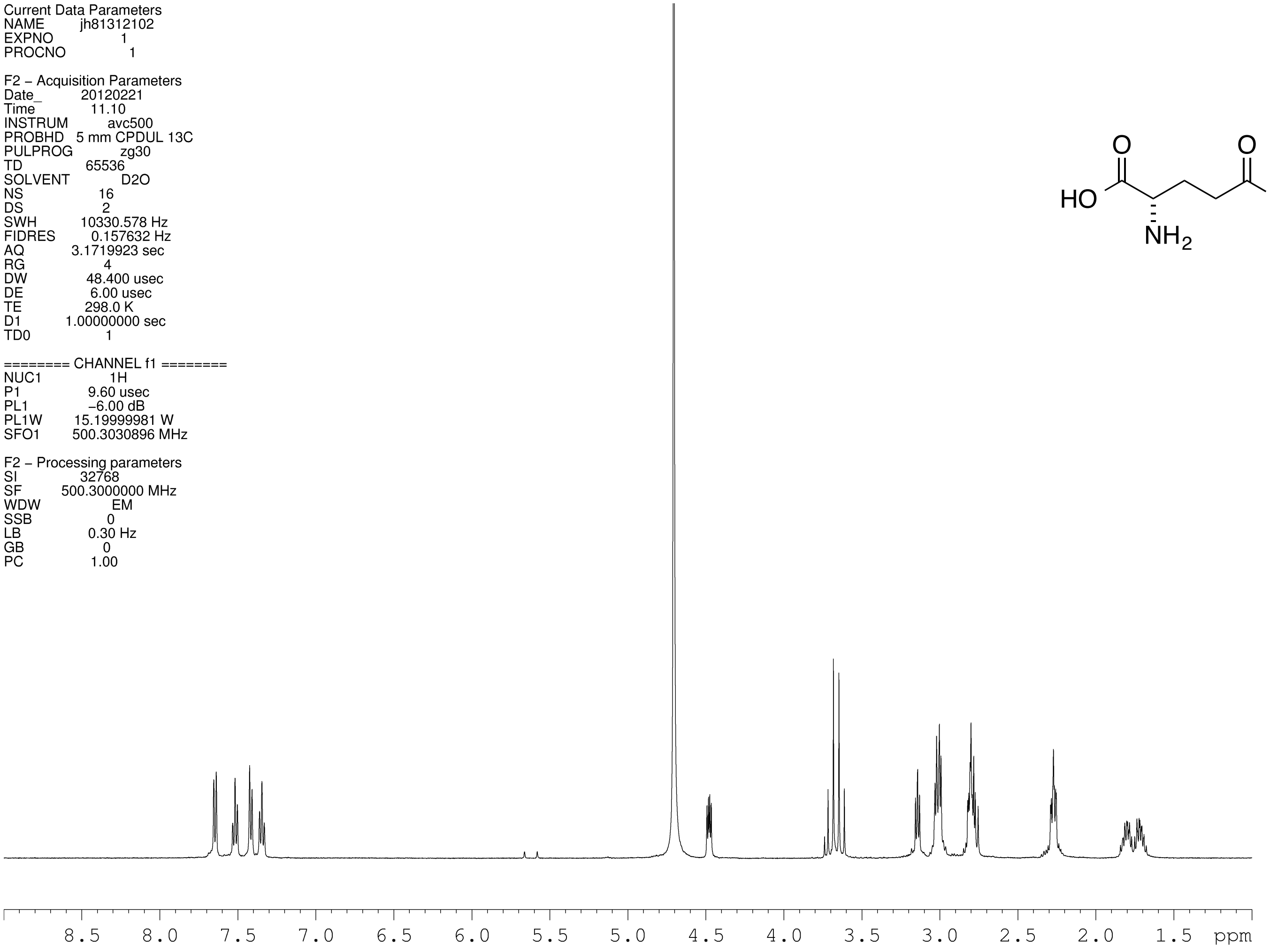

S-Ethyl-2-trifluoromethylphenyl glutathione (**6b**) – ^{13}C NMR

Current Data Parameters
NAME jh81312102
EXPNO 4
PROCNO 1

F2 – Acquisition Parameters
Date_ 20120221
Time 12.06
INSTRUM avc500
PROBHD 5 mm CPDUL 13C
PULPROG zgpg30
TD 65536
SOLVENT D2O
NS 2048
DS 2
SWH 31250.000 Hz
FIDRES 0.476837 Hz
AQ 1.0486259 sec
RG 1820
DW 16.000 usec
DE 20.00 usec
TE 298.0 K
D1 2.00000000 sec
D11 0.03000000 sec
TD0 1

======== CHANNEL f1 ========
NUC1 13C
P1 10.00 usec
PL1 -4.40 dB
PL1W 28.15752029 W
SFO1 125.8131151 MHz

======== CHANNEL f2 ========
CPDPRG2 waltz16
NUC2 1H
PCPD2 80.00 usec
PL2 -6.00 dB
PL12 12.42 dB
PL13 18.42 dB
PL2W 15.19999981 W
PL12W 0.21869738 W
PL13W 0.05493430 W
SFO2 500.3020012 MHz

F2 – Processing parameters
SI 32768
SF 125.8005350 MHz
WDW EM
SSB 0
LB 1.00 Hz
GB 0
PC 1.40

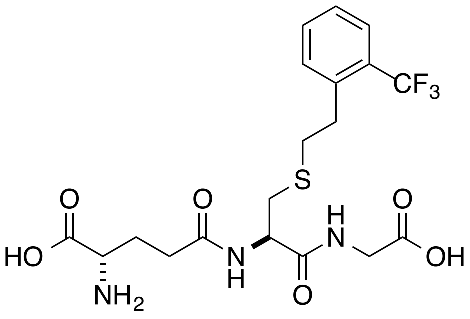

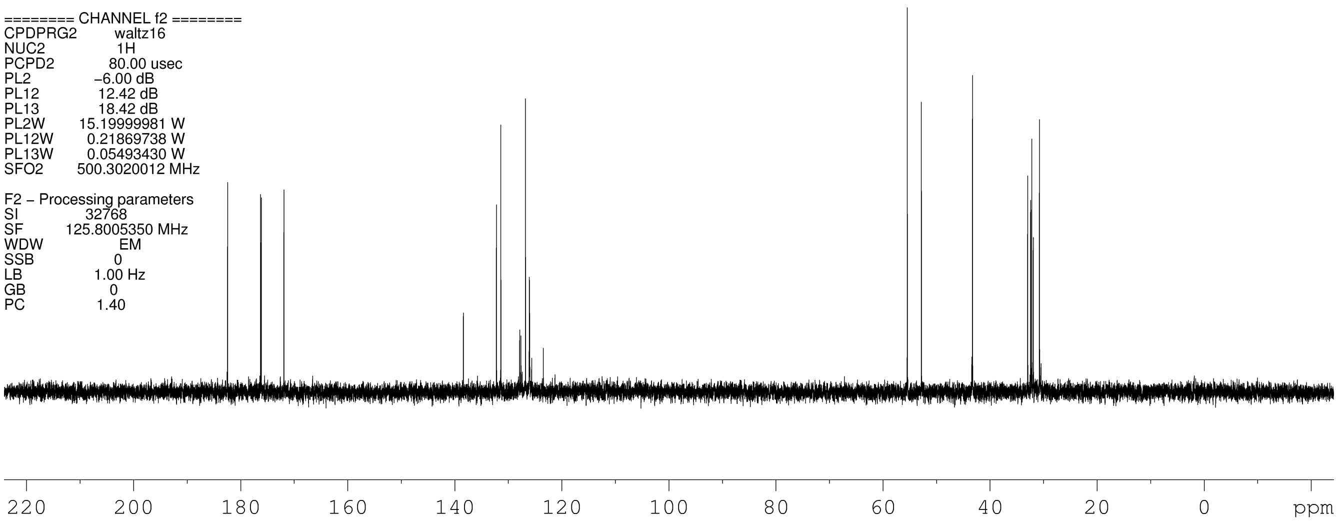

S-Ethyl-4-fluorophenyl glutathione (**6c**) – ^{1}H NMR

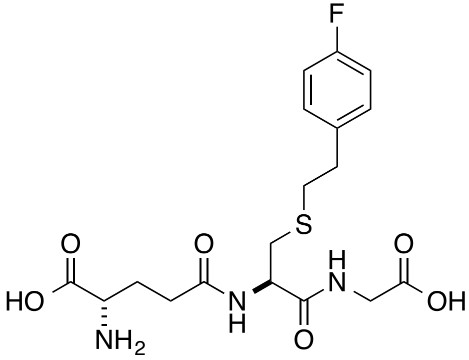

Current Data Parameters
NAME jh77650202
EXPNO 1
PROCNO 1

F2 – Acquisition Parameters
Date_ 20120202
Time 10.59
INSTRUM avc500
PROBHD 5 mm CPDUL 13C
PULPROG zg30
TD 65536
SOLVENT D2O
NS 16
DS 2
SWH 10330.578 Hz
FIDRES 0.157632 Hz
AQ 3.1719923 sec
RG 4
DW 48.400 usec
DE 6.00 usec
TE 298.0 K
D1 1.00000000 sec
TD0 1

======== CHANNEL f1 ========
NUC1 1H
P1 9.60 usec
PL1 –6.00 dB
PL1W 15.19999981 W
SFO1 500.3030896 MHz

F2 – Processing parameters
SI 32768
SF 500.3000000 MHz
WDW EM
SSB 0
LB 0.30 Hz
GB 0
PC 1.00

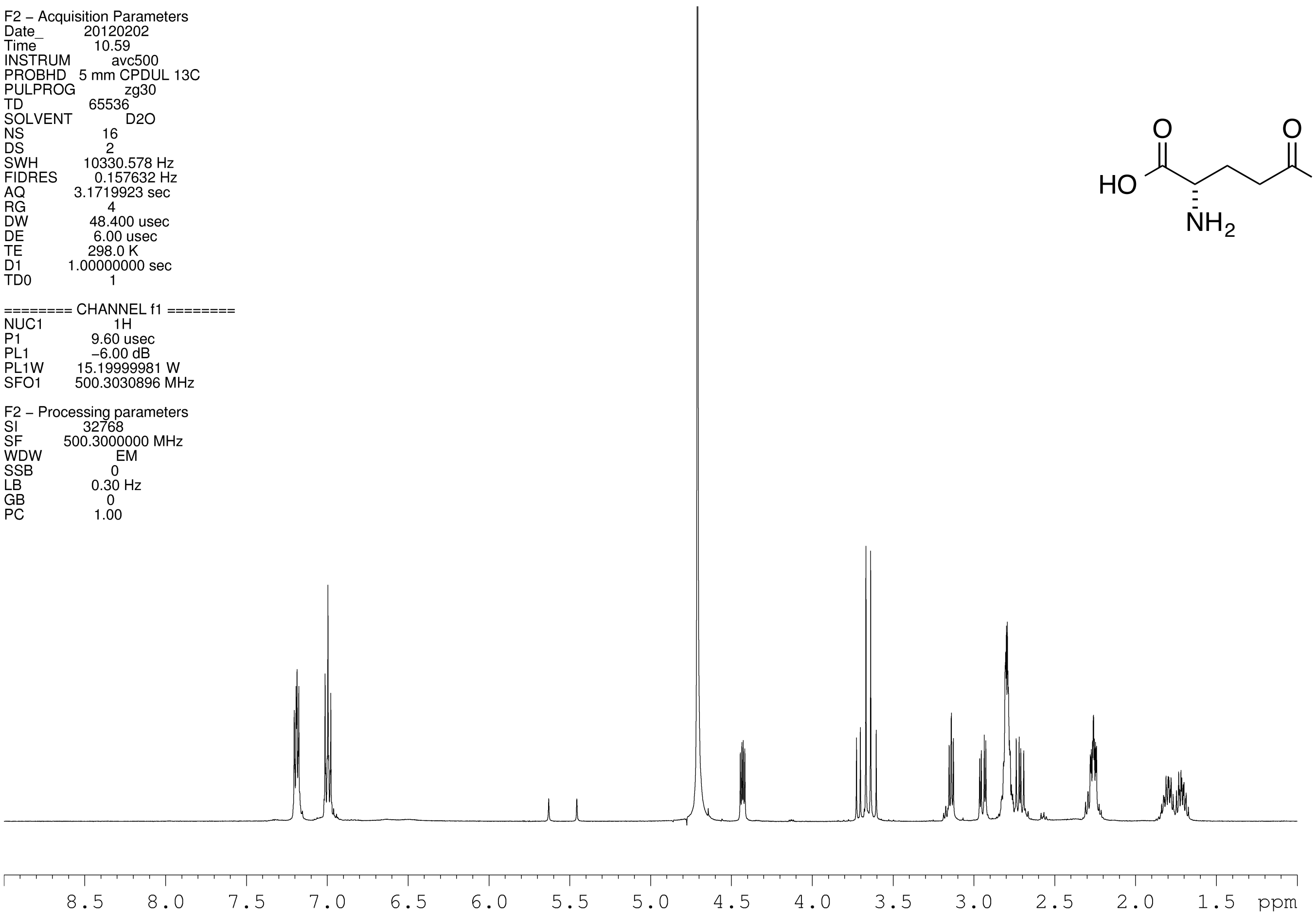

S-Ethyl-4-fluorophenyl glutathione (**6c**) – ^{13}C NMR

Current Data Parameters
NAME jh77650202
EXPNO 3
PROCNO 1

F2 – Acquisition Parameters
Date_ 20120202
Time 11.52
INSTRUM avc500
PROBHD 5 mm CPDUL 13C
PULPROG zgpg30
TD 65536
SOLVENT D2O
NS 2048
DS 2
SWH 31250.000 Hz
FIDRES 0.476837 Hz
AQ 1.0486259 sec
RG 1440
DW 16.000 usec
DE 20.00 usec
TE 298.0 K
D1 2.00000000 sec
D11 0.03000000 sec
TD0 1

======== CHANNEL f1 ========
NUC1 13C
P1 10.00 usec
PL1 −4.40 dB
PL1W 28.15752029 W
SFO1 125.8131151 MHz

======== CHANNEL f2 ========
CPDPRG2 waltz16
NUC2 1H
PCPD2 80.00 usec
PL2 −6.00 dB
PL12 12.42 dB
PL13 18.42 dB
PL2W 15.19999981 W
PL12W 0.21869738 W
PL13W 0.05493430 W
SFO2 500.3020012 MHz

F2 – Processing parameters
SI 32768
SF 125.8005350 MHz
WDW no
SSB 0
LB 0.00 Hz
GB 0
PC 1.40

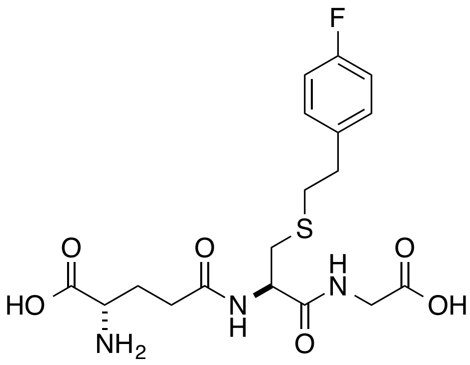

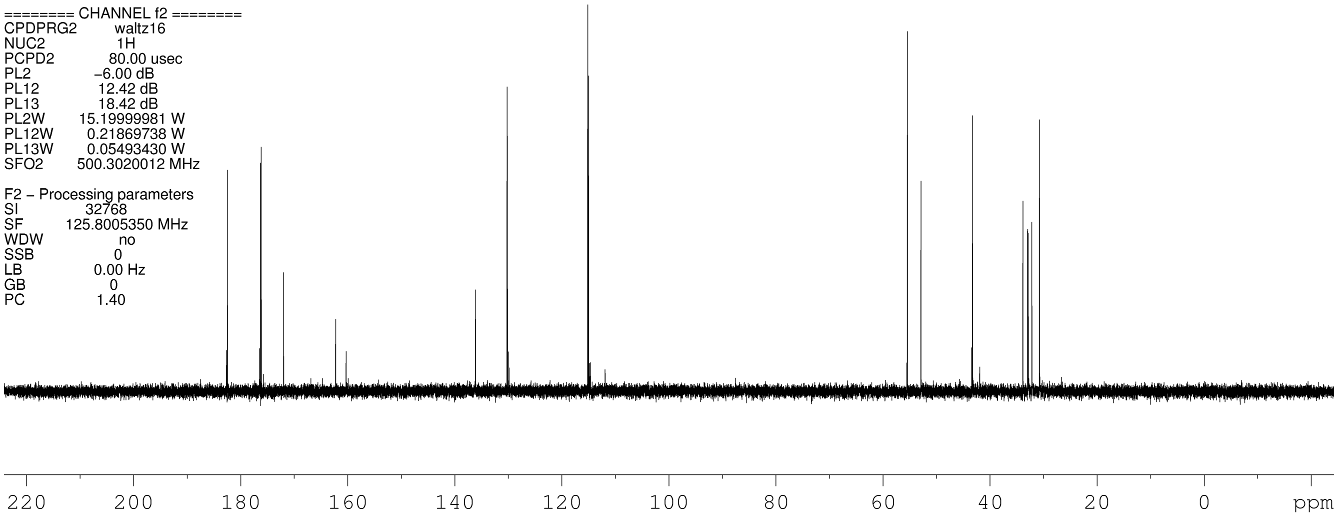

S-Propyl-4-fluorophenyl glutathione (**6d**) – ^{1}H NMR

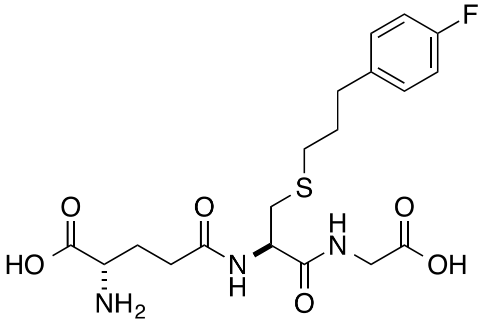

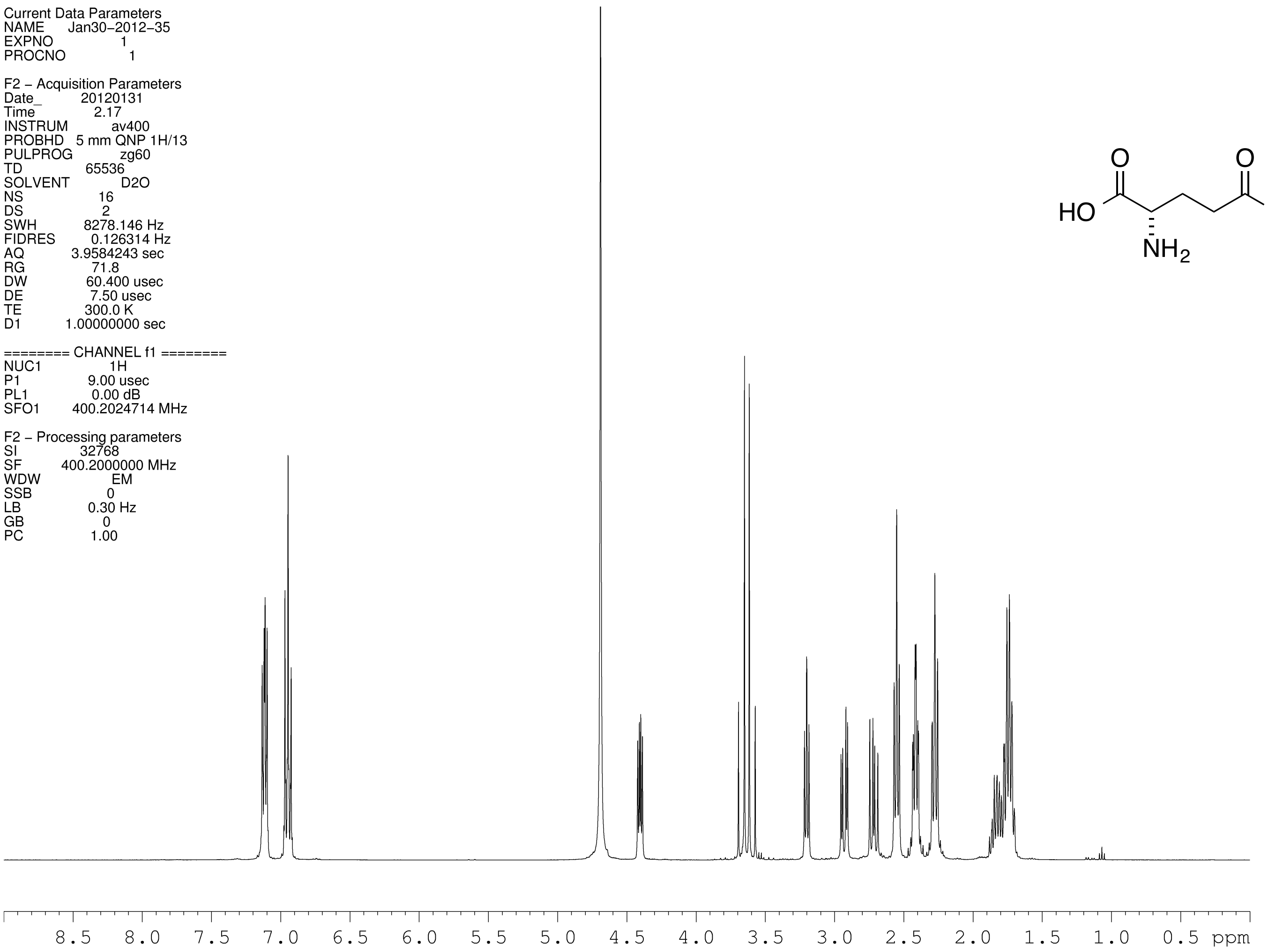

S-Propyl-4-fluorophenyl glutathione (**6d**) – ^{13}C NMR

Current Data Parameters
NAME Jan30–2012–35
EXPNO 4
PROCNO 1

F2 – Acquisition Parameters
Date_ 20120131
Time 2.30
INSTRUM av400
PROBHD 5 mm QNP 1H/13
PULPROG zgpg30
TD 32768
SOLVENT D2O
NS 256
DS 4
SWH 26178.010 Hz
FIDRES 0.798889 Hz
AQ 0.6259188 sec
RG 32768
DW 19.100 usec
DE 7.50 usec
TE 300.0 K
D1 1.00000000 sec
D11 0.03000000 sec
TD0 1

======== CHANNEL f1 ========
NUC1 13C
P1 9.50 usec
PL1 0.00 dB
SFO1 100.6403931 MHz

======== CHANNEL f2 ========
CPDPRG2 waltz16
NUC2 1H
PCPD2 80.00 usec
PL2 0.00 dB
PL12 19.00 dB
PL13 25.00 dB
SFO2 400.2016008 MHz

F2 – Processing parameters
SI 32768
SF 100.6303300 MHz
WDW EM
SSB 0
LB 1.00 Hz
GB 0
PC 1.40

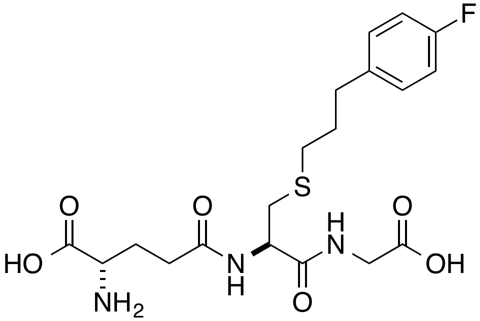

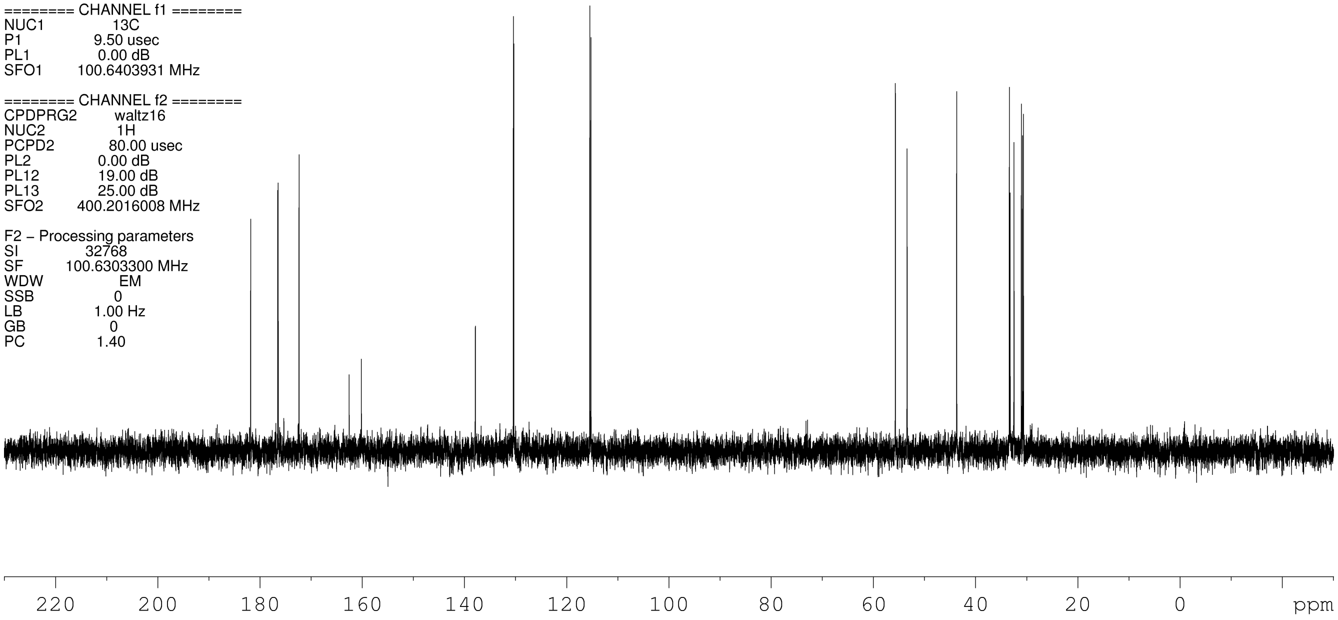

S-Phenylethylsulfone glutathione (**6e**) – ^{1}H NMR

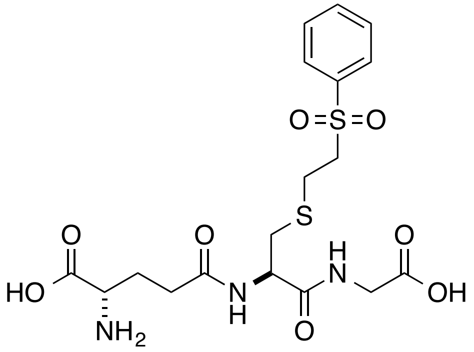

Current Data Parameters
NAME Jan31−2012−51
EXPNO 1
PROCNO 1

F2 − Acquisition Parameters
Date_ 20120131
Time 11.14
INSTRUM dpx400
PROBHD 5 mm Dual 1H/1
PULPROG zg60
TD 32768
SOLVENT D2O
NS 16
DS 2
SWH 5592.841 Hz
FIDRES 0.170680 Hz
AQ 2.9295092 sec
RG 574.7
DW 89.400 usec
DE 17.00 usec
TE 300.0 K
D1 1.00000000 sec

======== CHANNEL f1 ========
NUC1 1H
P1 7.30 usec
PL1 0.00 dB
SFO1 400.1320007 MHz

F2 − Processing parameters
SI 32768
SF 400.1300000 MHz
WDW EM
SSB 0
LB 0.30 Hz
GB 0
PC 0.60

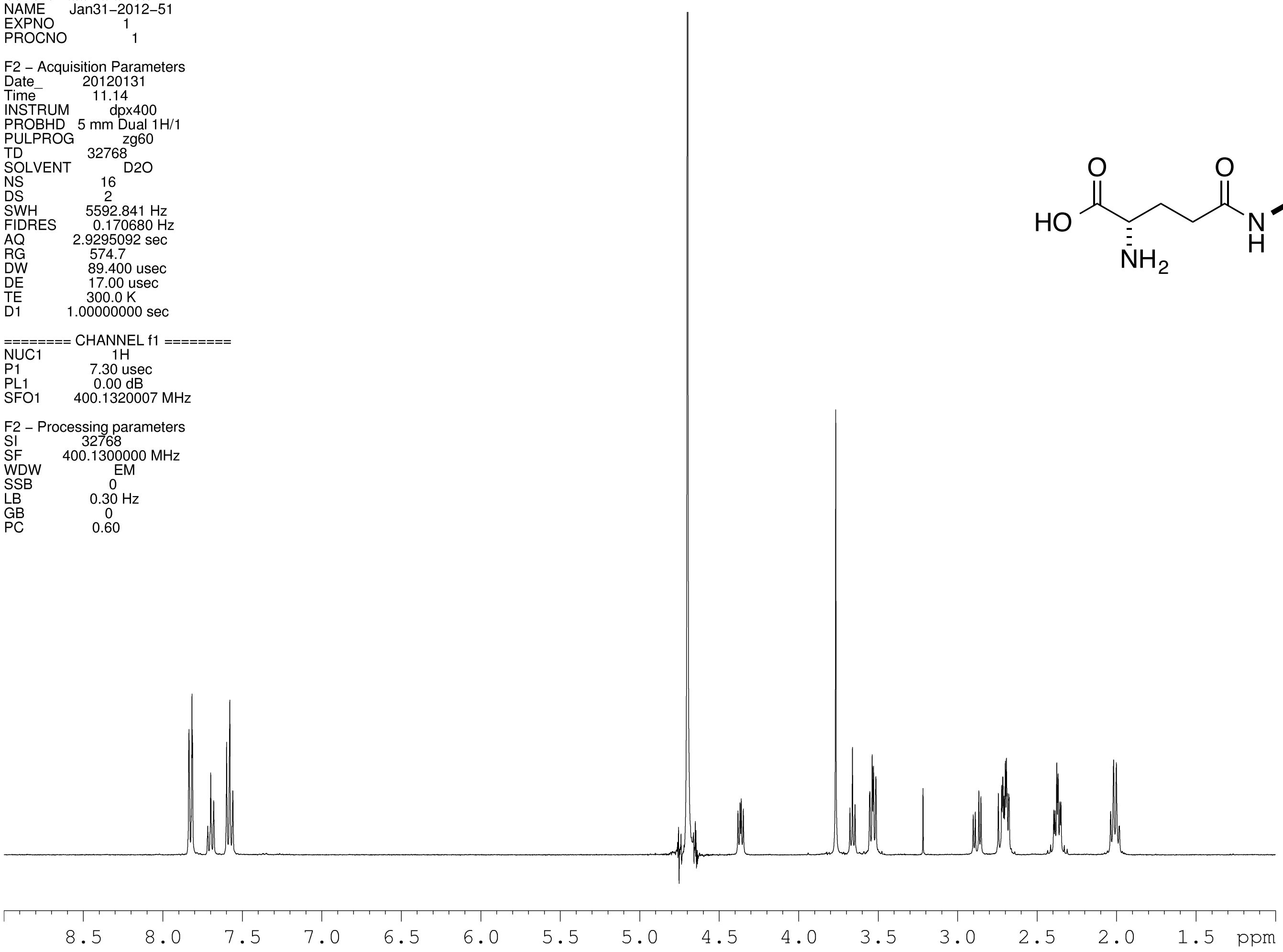

S-Phenylethylsulfone glutathione (**6e**) – ^{1}H NMR

Current Data Parameters
NAME jh77863101
EXPNO 4
PROCNO 1

F2 – Acquisition Parameters
Date_ 20120202
Time 14.55
INSTRUM avc500
PROBHD 5 mm CPDUL 13C
PULPROG zgpg30
TD 65536
SOLVENT D2O
NS 1024
DS 2
SWH 31250.000 Hz
FIDRES 0.476837 Hz
AQ 1.0486259 sec
RG 1820
DW 16.000 usec
DE 20.00 usec
TE 298.0 K
D1 2.00000000 sec
D11 0.03000000 sec
TD0 1

======== CHANNEL f1 ========
NUC1 13C
P1 10.00 usec
PL1 −4.40 dB
PL1W 28.15752029 W
SFO1 125.8131151 MHz

======== CHANNEL f2 ========
CPDPRG2 waltz16
NUC2 1H
PCPD2 80.00 usec
PL2 −6.00 dB
PL12 12.42 dB
PL13 18.42 dB
PL2W 15.19999981 W
PL12W 0.21869738 W
PL13W 0.05493430 W
SFO2 500.3020012 MHz

F2 – Processing parameters
SI 32768
SF 125.8005350 MHz
WDW EM
SSB 0
LB 1.00 Hz
GB 0
PC 1.40

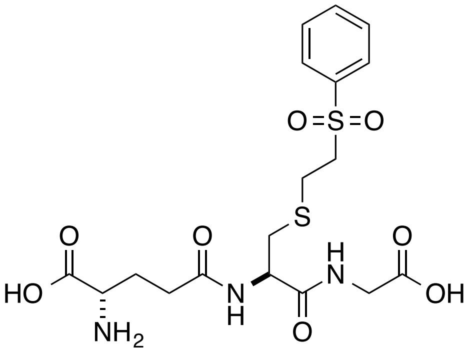

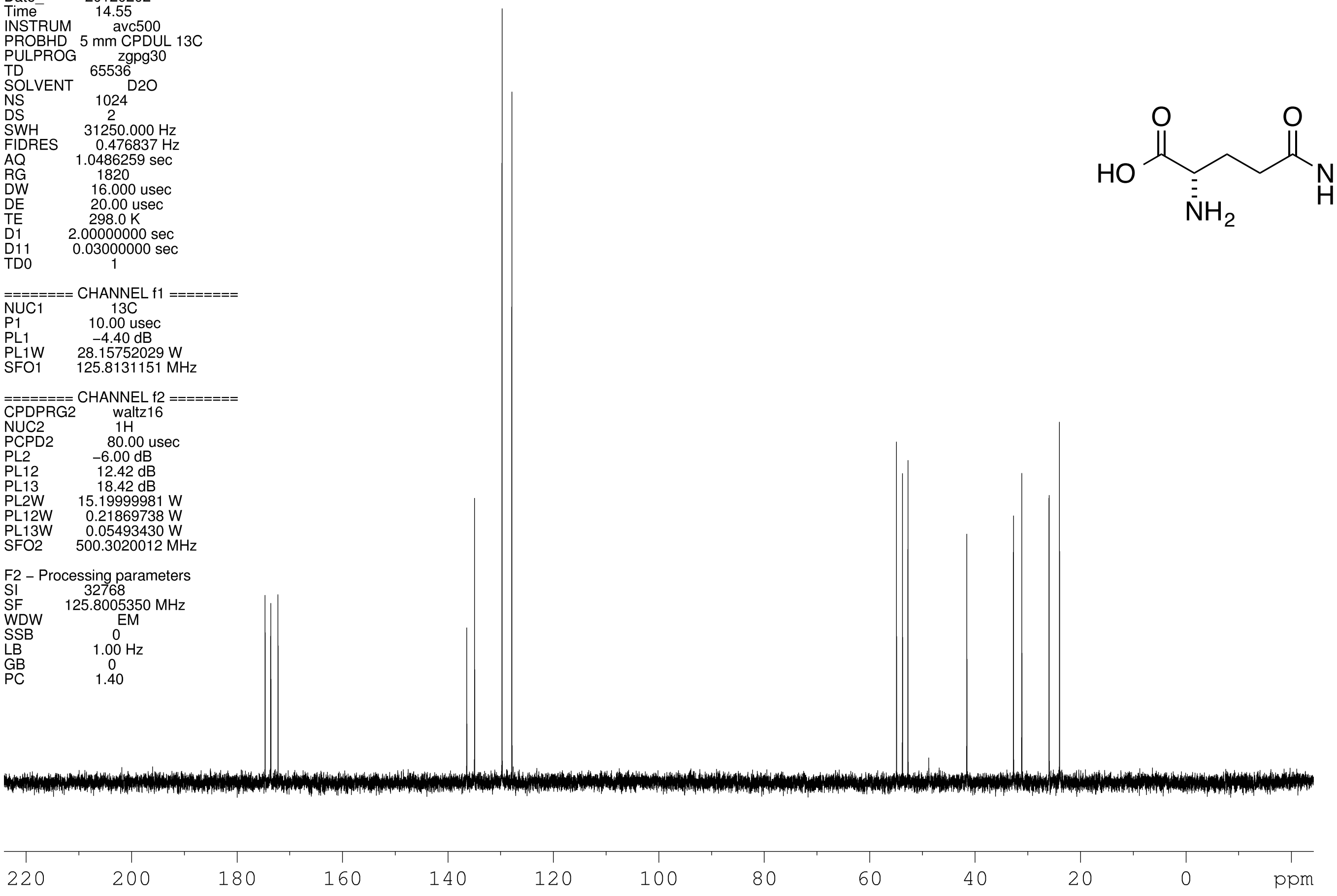

S-Phenylethyl glutathione (**6f**) – ^{1}H NMR

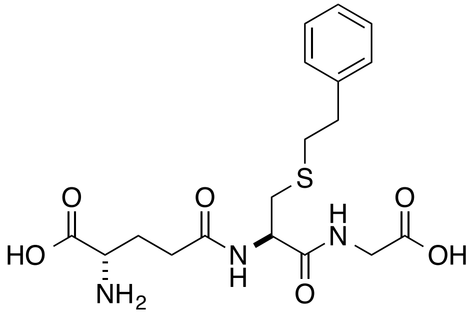

Current Data Parameters
NAME jh77640102
EXPNO 1
PROCNO 1

F2 - Acquisition Parameters
Date_ 20120201
Time 9.40
INSTRUM avc500
PROBHD 5 mm CPDUL 13C
PULPROG zg30
TD 65536
SOLVENT D2O
NS 16
DS 2
SWH 10330.578 Hz
FIDRES 0.157632 Hz
AQ 3.1719923 sec
RG 4
DW 48.400 usec
DE 6.00 usec
TE 298.0 K
D1 1.00000000 sec
TD0 1

======== CHANNEL f1 ========
NUC1 1H
P1 9.60 usec
PL1 -6.00 dB
PL1W 15.19999981 W
SFO1 500.3030896 MHz

F2 - Processing parameters
SI 32768
SF 500.3000000 MHz
WDW EM
SSB 0
LB 0.30 Hz
GB 0
PC 1.00

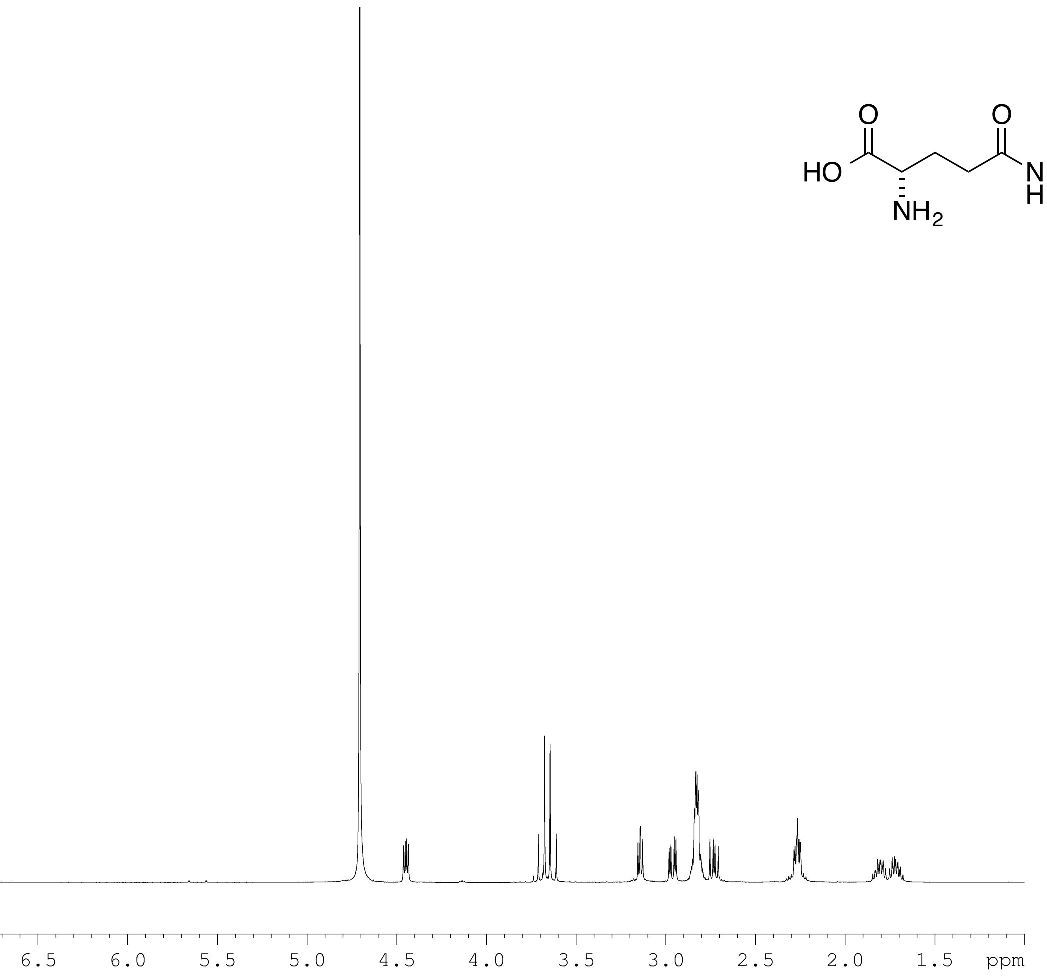

S-Phenylethyl glutathione (**6f**) – ^{13}C NMR

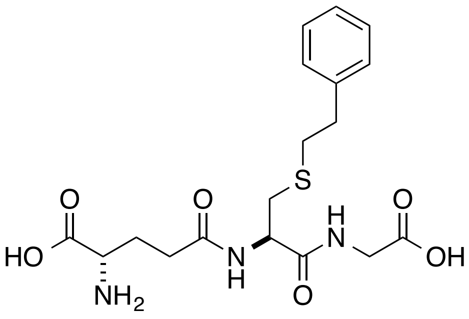

Current Data Parameters
NAME jh77640102
EXPNO 3
PROCNO 1

F2 – Acquisition Parameters
Date_ 20120201
Time 10.23
INSTRUM avc500
PROBHD 5 mm CPDUL 13C
PULPROG zgpg30
TD 65536
SOLVENT D2O
NS 1168
DS 2
SWH 31250.000 Hz
FIDRES 0.476837 Hz
AQ 1.0486259 sec
RG 1440
DW 16.000 usec
DE 20.00 usec
TE 298.0 K
D1 2.00000000 sec
D11 0.03000000 sec
TD0 1

======== CHANNEL f1 ========
NUC1 13C
P1 10.00 usec
PL1 −4.40 dB
PL1W 28.15752029 W
SFO1 125.8131151 MHz

======== CHANNEL f2 ========
CPDPRG2 waltz16
NUC2 1H
PCPD2 80.00 usec
PL2 −6.00 dB
PL12 12.42 dB
PL13 18.42 dB
PL2W 15.19999981 W
PL12W 0.21869738 W
PL13W 0.05493430 W
SFO2 500.3020012 MHz

F2 – Processing parameters
SI 32768
SF 125.8005350 MHz
WDW EM
SSB 0
LB 1.00 Hz
GB 0
PC 1.40

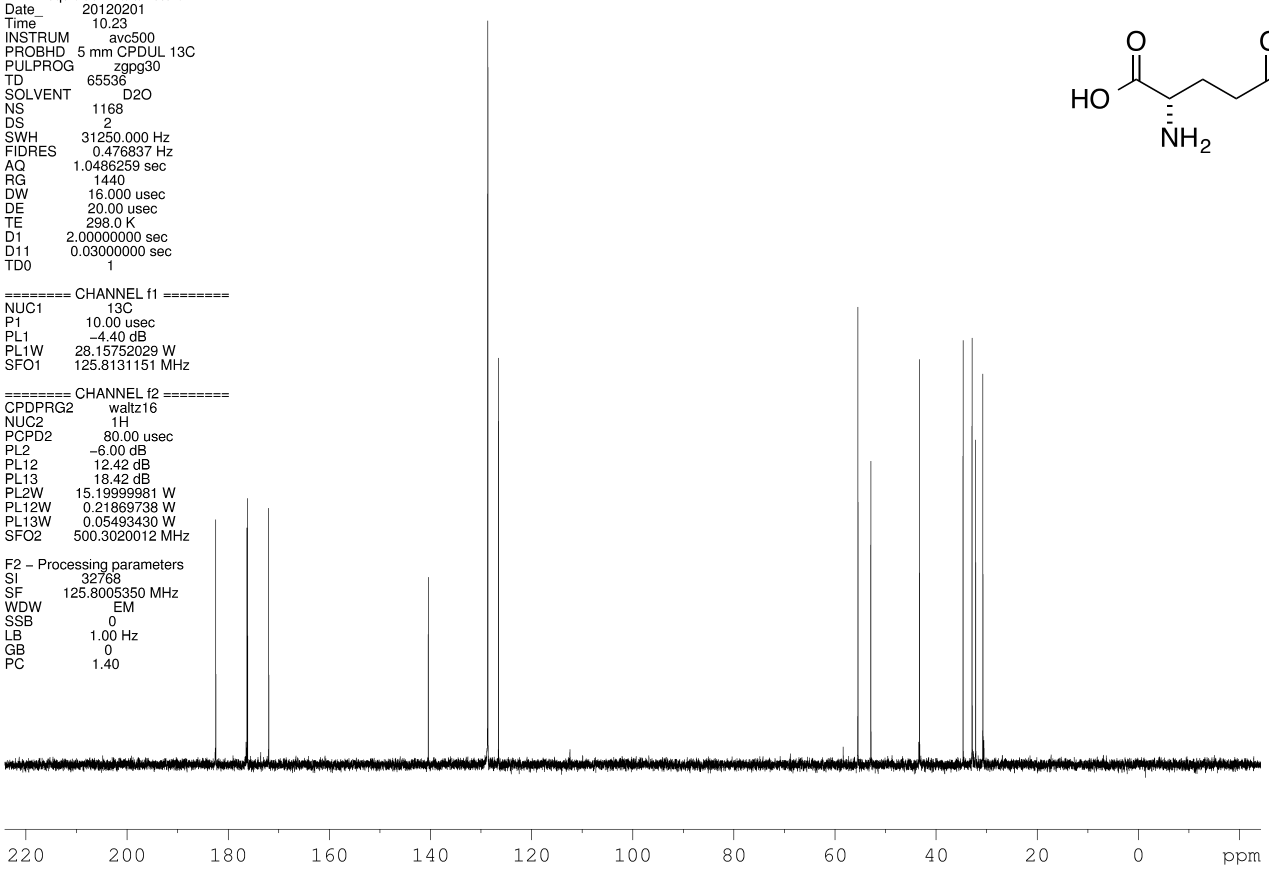

S-Ethyl-4-methoxyphenyl glutathione (**6g**) – ^{1}H NMR

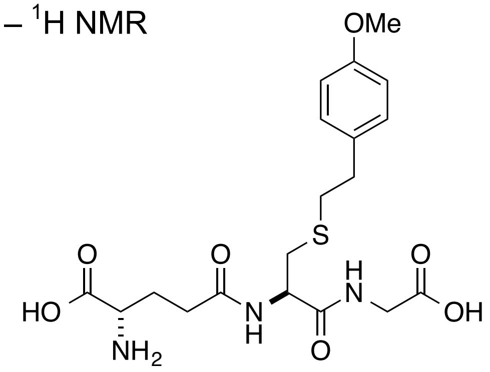

Current Data Parameters
NAME jh81292302
EXPNO 1
PROCNO 1

F2 – Acquisition Parameters
Date_ 20120223
Time 14.16
INSTRUM avc500
PROBHD 5 mm CPDUL 13C
PULPROG zg30
TD 65536
SOLVENT D2O
NS 16
DS 2
SWH 10330.578 Hz
FIDRES 0.157632 Hz
AQ 3.1719923 sec
RG 4
DW 48.400 usec
DE 6.00 usec
TE 298.0 K
D1 1.00000000 sec
TD0 1

======== CHANNEL f1 ========
NUC1 1H
P1 9.60 usec
PL1 –6.00 dB
PL1W 15.19999981 W
SFO1 500.3030896 MHz

F2 – Processing parameters
SI 32768
SF 500.3000000 MHz
WDW EM
SSB 0
LB 0.30 Hz
GB 0
PC 1.00

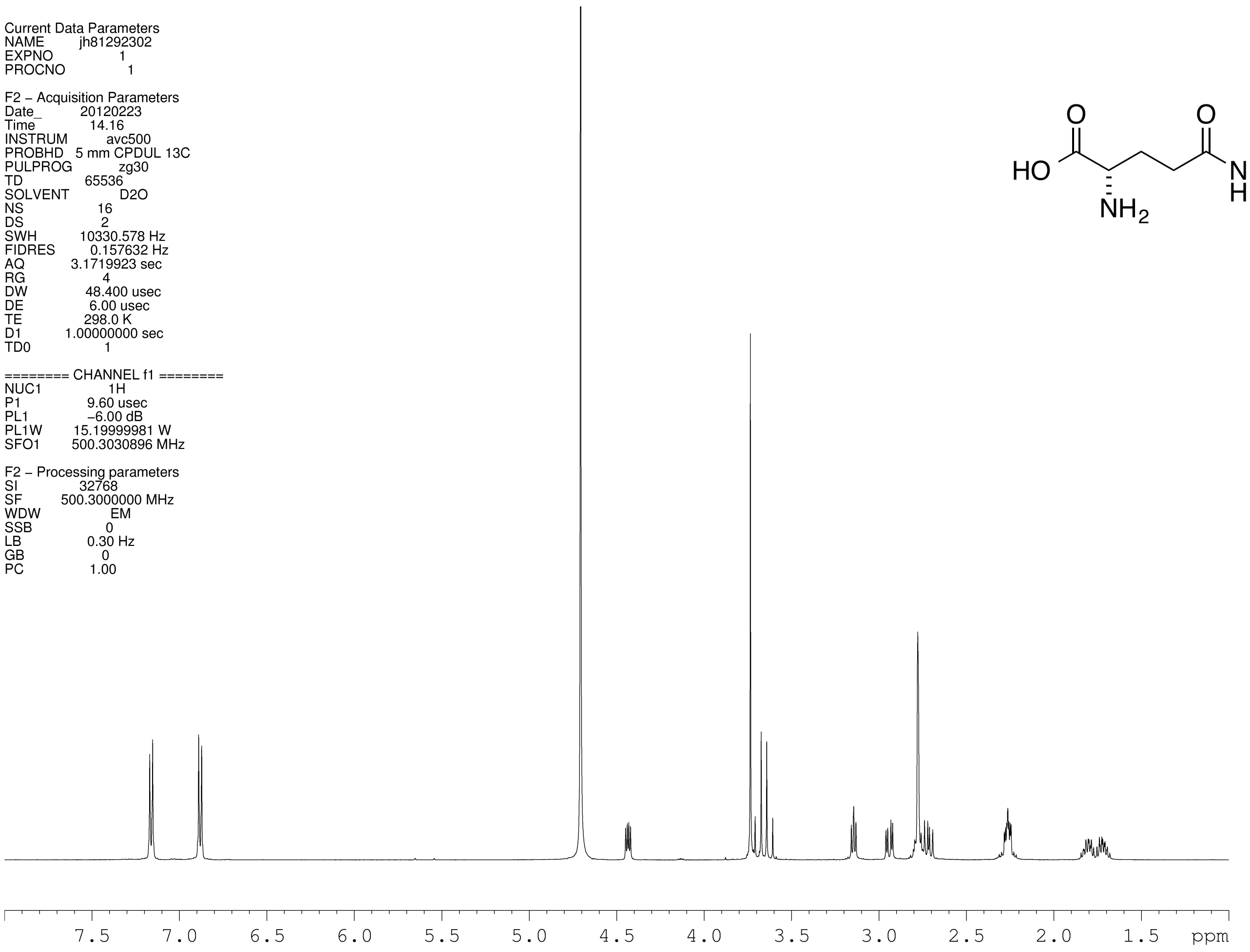

S-Ethyl-4-methoxyphenyl glutathione (**6g**) – ^{13}C NMR

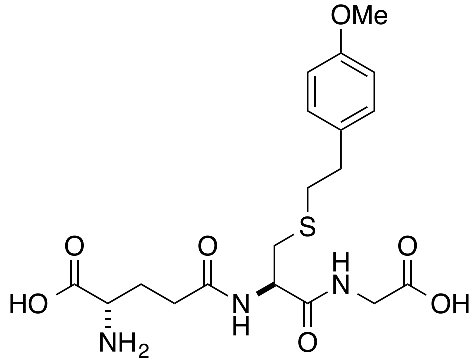

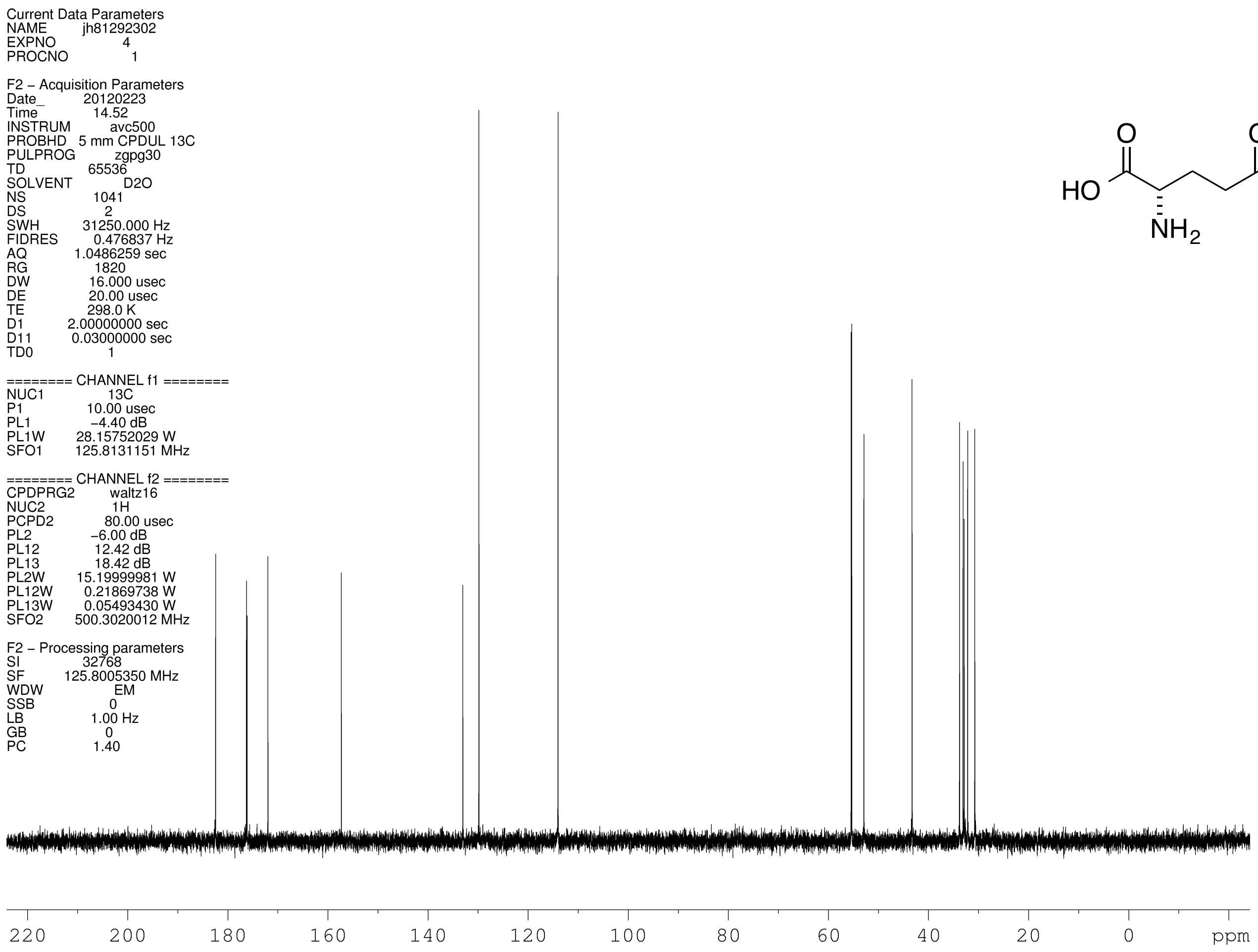

Supplement: Supplementary file 1 [file QO-003-C5QO00436E-s001.pdf]
